# Supplementary material for: Effects of common interest groups on rural women and youth livelihood: A qualitative study from Central Ethiopia
Source: PLoS One. 2023 Oct 20;18(10):e0283532. doi: 10.1371/journal.pone.0283532 (PMC10588890; doi:10.1371/journal.pone.0283532)
Supplement: S1 File — (DOC) [file pone.0283532.s011.doc]

**First order code**

1. An overview of the CIGs
2. Support from the AGP II coordination office
3. The CIGs and the local people other than its members
4. Members engagement in the group activities
5. The purpose of forming CIGs
6. The way forward to benefit from the CIGs
7. Threats the groups encountered
8. Opportunities both for the members and local people
9. Weaknesses of the groups
10. Strengths of the groups
11. A startup capital groups contribute, and their perception
12. The relationship between CIGs and other comparable groups
13. Perception of the members towards working in group
14. Members perspective on the futurity of their group
15. The benefits members gained
16. How the groups gained the working place
17. The situation of market linkage
18. The variation between their expenditure and income
19. The groups expenditure
20. Roles and responsibilities of the members
21. Problems the CIGs have encountered
22. Groups participation on the processes of buying the livestock
23. How members of the groups use money up-on their groups formation
24. Processes of group formation
25. Performance or effectiveness of the CIGs
26. The support from stakeholders

**Second order code**

An overview of the CIGs

1. The purpose of forming CIGs
2. Processes of group formation
3. How the groups gained the working place
4. Members engagement in the group activities
5. Roles and responsibilities of the members
6. Perception of the members towards working in group

Support gained from stakeholders

1. Support from the AGP II coordination office
2. The relationship between CIGs and other comparable groups

How members of the groups use money up-on their groups formation

1. A startup capital groups contribute, and their perception
2. Groups participation on the processes of buying the livestock

The groups expenditure

1. The variation between their expenditure and income

The benefits members gained

1. Performance or effectiveness of the CIGs
2. The CIGs and the local people other than its members

The situation of market linkage

Threats the groups encountered

1. Problems the CIGs have encountered

Opportunities both for the members and local people

Weaknesses of the groups

Strengths of the groups

The way forward to benefit from the CIGs

Members perspective on the futurity of their groups

**NB: The three alike themes: benefits the members gained, groups expenditure and How members of the groups use money up-on their groups formation are merged into one…i.e. performance or effectiveness ….and because of this the twelve themes presented here is reduced into 10 themes in the finding part**

**AND… The finding part twelve subtitles should appear in accordance with the second order coding….And the finding part should go in line with the themes developed during data collection and analysis time.**

- **Perception on the money they have had contributed….is merged with the theme’s regarding the selection criterion**

**Aggregate dimension**

**What are the activities performed by Common Interest Groups at the study area?**

1. An overview of the CIGs
2. Support from the AGP II coordination office
3. Members engagement in the group activities
4. The purpose of forming CIGs
5. A startup capital groups contribute, and their perception
6. The relationship between CIGs and other comparable groups
7. Perception of the members towards working in group
8. The support from stakeholders
9. How members of the groups use money up-on their groups formation
10. Roles and responsibilities of the members
11. Groups participation on the processes of buying the livestock
12. Processes of group formation
13. How the groups gained the working place

**How effective is the implementation of activities of Common Interest Groups at the study area?**

1. The variation between their expenditure and income
2. The groups expenditure
3. The CIGs and the local people other than its members
4. The benefits members gained
5. The situation of market linkage
6. Performance or effectiveness of the CIGs

**What is the SWOT of the Common Interest Groups implementation at the study area?**

1. Threats the groups encountered
2. Opportunities both for the members and local people
3. Weaknesses of the groups
4. Strengths of the groups
5. Problems the CIGs have encountered

**What intervention strategies could be designed to enhance the operation of Common Interest Group in the study area?**

1. The way forward to benefit from the CIGs
2. Members perspective on the futurity of their groups
