# Supplementary material for: Effects of common interest groups on rural women and youth livelihood: A qualitative study from Central Ethiopia
Source: PLoS One. 2023 Oct 20;18(10):e0283532. doi: 10.1371/journal.pone.0283532 (PMC10588890; doi:10.1371/journal.pone.0283532)
Supplement: S2 File — (DOCX) [file pone.0283532.s012.docx]

**What are the activities performed by Common Interest Groups at the study area?**

1. How members of the groups use money up-on their groups formation

The saving from the members was used mainly to construct an abode, a place to keep the cattle (cows and calves). With the financial support from AGP, they bought 8 cattle mostly of which are cows which were meant for the diary production. The group had a total of 17 cattle, and sold two of them in the last three years. (FGD 1_Dairy farm_Lencho Borsu, Pos. 24)

The respondents have said that when they commence the business, they were able to raise 1660 birr from each of the members and supported with 50,000 birr from AGP II with which they bought 55 sheep. (FGD 2_Sheep fattening_Dhaaye Tuti, Pos. 19)

They said the group was able to collect about 22,000 birr and they got 66000 birr from AGP. With that money, they said they constructed abode for the poultry. They also said AGP has given them 1200 small poultry but many of them dead with few surviving.  (FGD 3_Poultry production_Wale Chilalo, Pos. 11)

1. A startup capital groups contribute, and their perception

The other perception area examined by the interviewer is whether the members assume the initial money they had to contribute is expensive. They responded that the contribution was not that demanding for them but of course some members felt a bit of pressure to fulfill the expectations easily. They also said the group members knew that the contribution was meant to initiate the business and it will be saved for the future risk aversion. (FGD 2_Sheep fattening_Dhaaye Tuti, Pos. 25)

The discussants said they were 20 women when they start the business in 2010, and each of them contribute 1200 birr which they had to pay as part of the initial capital which accounts for 25% while the rest is contributed by AGP. They said the group was able to collect about 22,000 birr and they got 66000 birr from AGP. (FGD 3_Poultry production_Wale Chilalo, Pos. 11)

1. Groups participation on the processes of buying the livestock

The discussants said they have participated in the process of buying the cattle particularly after they consulted with the AGP experts that they need to buy the cattle that better adapt to the local weather condition. The members and the technicians from AGP together bought the conducive cow type that can give a better product and adopt better to the local weather condition. From their participation, the discussants said, they benefited as most of the cows bought are mostly of productive if not one of the cows that fail to give good milk but a calf which was sold for about 7000 birr. (FGD 1_Dairy farm_Lencho Borsu, Pos. 26)

The task of oxen fattening was chosen by the interest of the group members and they took training two times with. The members also saved about 24% and 14k in total amount. (FGD 4_Oxen fattening_Abo Yayambana, Pos. 21)

The oxen were bought from the town of Goha-Tsion/Qarre-Goha. However, the group wanted to buy but they can’t, and the woreda level individuals did not allow them to do so. 84000 birr in total bought about 7 but 14000 birr was confiscated at the woreda level. (FGD 4_Oxen fattening_Abo Yayambana, Pos. 22)

When buying these animals, neither the villagers nor the local DAs take part in the buying process. (Zeleke Hailu_Livestock and fishery development office, Pos. 14)

The team is formed and they buy the animals for the CIGs. The local administrators do not participate in buying the animals also. They cannot participate because of the principles that only look for the engagement of animal workers and financial sector. (Teshome Tolossa_Women and youth affair office, Pos. 5)

1. The support from stakeholders

The support comes from all stakeholders like the livestock office, youth and women affairs, and cooperatives agencies. All have stake in the CIG groups. DA also working in cooperatives with the members and they give routine assistance and supports. However, the support rendered is not enough and there are yet to be done in satisfying needs. DAs also support but the main activities of buying the cattle’s and oxen excludes them and accomplished by the AGP, Cooperatives organizations, and the financial agencies of the woreda. The DAs may also consider the AGP activities as extra-work and they may not give as much attention as the activities need. (Bikila Tolossa_AGP coordinator, Pos. 64)

The cooperatives help saving, enhance their income level, and encourage them to join the local cooperatives including the saving and credit cooperatives and multi-purpose cooperatives. However, many problems exist for this implementation. This is because the members are mostly of poor. The CIG is based on the monthly payment which they cannot and the cooperatives also require them buying sharing during the time of joining one them. However, those better off individuals are joining the cooperatives. The poor does not fit these expectations. In addition, the conflicts most often rise among the members and the way they are organized was a problem for the later conflict among the members. (Debebe Zeleke_cooperative development office, Pos. 9)

The WALQO works on the business plan and the members start saving which is about 25% and the rest will be paid of AGP. In the sector, members mostly take part in animal fattening. The sector checks the animal about their health and the possibility of fattening. It is after that they buying the animal takes place and cattle bought by the CIGs are followed up for their best. They work on fattening the cattle to be able to get the product needed with three months and mostly with the support of technologies. (Zeleke Hailu_Livestock and fishery development office, Pos. 13)

The respondent explained that when there is a problem among CIG members, the stakeholders’ team reforms the CIGs. The respondent said they particularly focused on reforming of the CIGs where they are performing better. (Teshome Tolossa_Women and youth affair office, Pos. 5)

1. The relationship between CIGs and other comparable groups

They also said although another group consisting of women exists in the village, but they were not that close to them despite rare information they had to share among themselves. (FGD 2_Sheep fattening_Dhaaye Tuti, Pos. 24)

1. Support from the AGP II coordination office

They work on both households without discriminating against the type of households. They encourage youths to engage in CIG, and they encourage women and men also. They do not exclusively support either of male headed households nor female headed households. They do not have a gender disaggregated also. (Bikila Tolossa_AGP coordinator, Pos. 55)

The main activity of the Women and youth office is creating the awareness about the CIGs and its benefits after which the youths and women learn of joining. However, the respondent has said, there is no uniform knowledge among the stakeholders and the CIG members. (Teshome Tolossa_Women and youth affair office, Pos. 3)

1. An overview of the CIGs

The 24 men youth CIG and 19 women total 43 CIG were supported at the woreda level. They are engaged in various agricultural related activities and they focus more on animal fattening. Dairy Farming, Oxen fattening, Sheep production, Goat Production, sheep and goat fattening, and poultry production are the main types of CIG working areas. (Bikila Tolossa_AGP coordinator, Pos. 57)

In principle, they need to have similar interests and the criterion is that a member of a family can join a CIG and should be credit-free individuals. The age also matters for forming the CIGs. The working age (18-55) are preferred to others. The AGP helps 75% of the initial capital while the members contribute the rest. The money given is‘seal’ money (Seal-may not be the appropriate spelling). If a member leaves a group, its only profits that he/she takes out while leaving. (Bikila Tolossa_AGP coordinator, Pos. 60)

The cooperative office of the woreda takes parts as one of the stakeholders in AGP.  The cooperatives particularly want to encourage the CIG members to enhance their livelihoods and join the cooperatives-the multi-purpose cooperatives. (Debebe Zeleke_cooperative development office, Pos. 3)

There are 44-46 CIGs established during this AGP 2 period. (Debebe Zeleke_cooperative development office, Pos. 7)

CIGs exist in all villages all over the 25 villages. And the respondent said there is 2 CIGs in each kebele one of which is youths’ and the other belongs to women. (Zeleke Hailu_Livestock and fishery development office, Pos. 15)

The respondent has said the CIG was formed because of the awareness raising conducted in the villages found in the woreda mostly by the stakeholders. 50 CIG was planned to be established which is two of them in a village.CIG is established in collaboration with the cooperative offices. (Teshome Tolossa_Women and youth affair office, Pos. 3)

The respondent has said that one of the criteria for selecting the farmers for the CIG are that they had to have common interest and initiation to work together for change. In the village, two CIGs availed and one consisted of 12 men who fatten oxen and the other group which consisted both men and women produce sheep. (Tesfaye Tewabe_DA_Abo Yayambana, Pos. 29)

1. Roles and responsibilities of the members

Each members of the group is responsible to watch after the cattle and feeding them. They said, they allocate days and time for the members who would take care for the cattle once in 19 days since they are totally 19 members. A member on his/her duty day would watch the cattle, clean their abode, and feed them. In addition, the discussant said, the members have obligation to abide the rules and regulation of the group and if there is a failure in that regard, they would face a punishment of 30 birr in the first instance, 50 birr for the second time. But the members shown increasingly important and they genuinely support and abide the rules and regulations. The accountant and monitory body of the group are also obligated to save their financial resource to the local bank on time and withdraw when the group interested to do so, and mostly on time. The monitoring body also follows the members and their activities, the cattle and the financial budget. (FGD 1_Dairy farm_Lencho Borsu, Pos. 34-36)

The members care for the oxen on routine. Some group members are, however, reluctant. The quarrel happened and they informed the woreda level stakeholders and they gave 8 individuals their saving and the group members were minimized to 12. (FGD 4_Oxen fattening_Abo Yayambana, Pos. 21)

1. Perception of the members towards working in group

Being in group entity itself has its own positive sides, say the respondents. Working in group gave them numerous benefits which each of them could not accomplish on their own and at ease. More importantly, it is providing a sustainable means of livelihoods and employment for the members. (FGD 2_Sheep fattening_Dhaaye Tuti, Pos. 22)

Although the business is perfect, the support from the government side was discouraging. They could not get service from the stakeholders; they lacked inputs and reserves where they can keep the oxen. They did not get training on the routine basis also. They assume there would have been more benefits had they followed the training appropriately. (FGD 4_Oxen fattening_Abo Yayambana, Pos. 28)

The group is supposed to have the same interest and resides in the same area. However, as the number of members is increased, the effectiveness reduced because there were many ideas. Had the group build from only 3-5, they could have the same idea and become more effective. But as the number increased, there would be an increasing interest among the members. (FGD 4_Oxen fattening_Abo Yayambana, Pos. 38)

1. Members engagement in the group activities

The 12 members divided in to two having 6 members each. And later they divided in to three individuals and they kept those cattle at the side. They sold three times to the market thus far. They bought 7 more oxen which were of the best quality type, and they sold them within 2 months. In each of them, they got 700-1400birr revenue. The members wanted to buy more of oxen but they faced obstacles from the woreda stakeholders. Nevertheless, they later on bought 9 oxen and each group has 4 and 5 oxen. Although some fatten, others did not. This also created a conflict within the members. (FGD 4_Oxen fattening_Abo Yayambana, Pos. 24)

1. How the groups gained the working place

They rented a place where they can keep the sheep. Since the sheep are many, they shared them among 5 of them also.  (FGD 2_Sheep fattening_Dhaaye Tuti, Pos. 17)

They were told they would be given the place where they can keep their animal. And also materials used for constructing but they failed to do that. Then, the members decide to rent a place recommended by the village leader, and could not build a house for the rest. (FGD 4_Oxen fattening_Abo Yayambana, Pos. 23)

1. The purpose of forming CIGs

The discussants have said that their main motive of forming a CIG was for the purpose of upbringing the poultry and selling their products. (FGD 3_Poultry production_Wale Chilalo, Pos. 13)

1. Processes of group formation

The group was formed from15 men and 4 women totaling 19 members. When they commenced the business, they were given 90,000 ETB by the government and its program of AGP, and on that each of the members saved 1250 birr which totals about 27,800 birr. (FGD 1_Dairy farm_Lencho Borsu, Pos. 24)

The respondent said the group he belongs to which has 10 members started to operate shortly after their formation in 2010 E.C. Though the group whom all are men has no name yet, it is located in Dhaaye Tuti village/kebele. The respondent stated that they came together and form their group basically based on their self-reported interest which of course supported by the awareness raising campaign held in their village and at the woreda level as well. The group also opted to fattening and producing sheep and he reiterated that choosing the sheep related to their interest but did not highlight the specific rationale of choosing the sheep production. (FGD 2_Sheep fattening_Dhaaye Tuti, Pos. 16-17)

The discussants said they were 20 women when they start the business in 2010, and each of them contribute 1200 birr which they had to pay as part of the initial capital which accounts for 25% while the rest is contributed by AGP. They said the group was able to collect about 22,000 birr and they got 66000 birr from AGP. With that money, they said they constructed abode for the poultry. They also said AGP has given them 1200 small poultry but many of them dead with few surviving.  (FGD 3_Poultry production_Wale Chilalo, Pos. 11)

The group was established in 2010. First, the stakeholders from the woreda came with the village leader and talked to them. The AGP II organized individuals who happen to have similar interest and residing in the same area. The village leader gave them the list and all of the listed individuals attended the meetings, and 20 individuals came together and form the group. They also took part in the woreda level training. They were given 70,000 birr. (FGD 4_Oxen fattening_Abo Yayambana, Pos. 20)

Previously the group consists of 20 members but through times they become 12. The group established in 2010. (FGD 4_Oxen fattening_Abo Yayambana, Pos. 18)

**How effective is the implementation of activities of Common Interest Groups at the study area?**

The situation of market linkage

The discussants said there is no market linkage and they sell their product-butter, on the own. They sell 5 kilo of butter one in a week at the local market place. (FGD 1_Dairy farm_Lencho Borsu, Pos. 41)

The respondents have said that they were told by the local government that some kind of market linkage would be created for them during their initial meeting. However, they criticized that there was no such kind that has been facilitated thus far and they depend on the local market for the marketing purpose. The market linkage facilitation didn’t happen yet. This happened, according to the respondent, that because the government entities, AGP II coordination office, and CIG groups by itself are all reluctant to do so regardless of the group’s attempts of producing more sheep as the years pass. (FGD 2_Sheep fattening_Dhaaye Tuti, Pos. 27)

Although during the training, they were told they would get more access to market, that promise never existed. On the second term, none of the members got service form any of the stakeholders and they were discouraged in their business, they say. (FGD 4_Oxen fattening_Abo Yayambana, Pos. 30)

The respondent has said, yes, it is needed but the CIG are not developed enough to that level yet. As CIGs develop, (The office of market development) market linkage is a way important and they need market linking agents in this regard. The respondent said the dairy cooperatives may help, but there is no that great numbers of CIG working in dairy farming and there is limited number of them and they supply low. SO, there is no motive to link them with the organizations found in other areas. But in the future, it is likely that they can arrange in this regard (Debebe Zeleke_cooperative development office, Pos. 23)

1. The benefits members gained

They explained that the group membership was enabled them to work collaboratively, and it was not merely for the purpose of revenue making but meant for sustaining livelihoods and generating incomes for their daily needs. (FGD 2_Sheep fattening_Dhaaye Tuti, Pos. 19)

The business helped the members to get employed which most of them used to dearth and their productivity is increasing through time. They mentioned that when they sold the sheep for 62,000 birr in the first year of their formation and they shared the revenues among themselves and each got 6000 birr. They had to share the revenues at the time basically because, they said, they need money to buy agricultural inputs such as fertilizer. They also said, of course they are selling sheep then on but the 62,000 birr they once got is the highest income recorded. Moreover, although initially they had 55 sheep, at the time of this interview, they have about 80 and more sheep now. Emphasizing their effectiveness, they also added that despite the income sharing they have to conduct when they want, they said they also have unofficial amount of money with the group accountant. (FGD 2_Sheep fattening_Dhaaye Tuti, Pos. 20)

The CIG has sold about 622 hens during their functioning time and able to garner 39500 birr in general and they shared 1975 from this output. However, the income they got did not commensurate the efforts they paid and the requirement for upbringing hens also. It takes three months for small hens to be grown fully, but the group could not feed poultry through these times because the feeding is expensive and can incur more expense than the income they generate from the production. (FGD 3_Poultry production_Wale Chilalo, Pos. 15)

The discussant said regardless of the malfunctioning of the group as it supposed to operate; they garnered benefits in terms of motivation and experience. They also said they were able to create social capital and the group served as a source of information sharing. (FGD 3_Poultry production_Wale Chilalo, Pos. 18)

In general, they sold the oxen three times and in the first round, they got a profit of 700-1400 birr from each, and the second round they got 1200 birr of profit from each. Other time, they got 110-1600 profit. They divided the profit among themselves, and but the stakeholders did not recommend that. (FGD 4_Oxen fattening_Abo Yayambana, Pos. 25)

Due to various problems they had to face including the lack of inputs for fattening purposes, they dissolved the group and share the oxen among themselves. Nevertheless, they were beneficiaries even if the group is dissolved. For instance, the leader bought an ox for 16000 birr and later on sold it for 22,000 within 6 months. Most of them, however, shifted the oxen fattening for another activities like shopping, buying inputs and ploughed lands and etc. (FGD 4_Oxen fattening_Abo Yayambana, Pos. 26)

Few of them are still unified and getting more benefits- dairy production is exemplary in this regard. (Bikila Tolossa_AGP coordinator, Pos. 59)

1. The CIGs and the local people other than its members

They were supported by then local people, but they did not negatively affected. Some of the members even gave the group a grazing place for the member and encouragement. (FGD 4_Oxen fattening_Abo Yayambana, Pos. 40)

The respondents said, the number of group should be reduced and each of the group has to get to know one another and work as such. The people who builds a group should be those who live in the same residence area, know one another and their effectiveness and all of that and they need to select who should join and not. So, he said, when they are formed they should be from the same village and each of the members’ effectiveness and background should be assessed. The government should also monitor and follow the group per week or a month. As of the respondent, there was no such monitoring activity from the government side. The governmental stakeholder did not clearly and carefully control the group’s works. Follow up should be aligned with controlling behaviors and punishments as well. The government should not be reluctant in that regard. (FGD 4_Oxen fattening_Abo Yayambana, Pos. 42-43)

1. Performance or effectiveness of the CIGs

There a high development of CIGs that sold 4 or more times for instance the Jemjem CIGs. The other few also sold their products once or twice and benefited. Others CIGs can be considered as low performing CIGs and such CIGs dissolved. In light with the plans, the respondent considered the CIG performance is mostly of medium. The plan is how to raise the benefits but some are dissolved but they also benefited. So they consider the performance of CIGGs is medium performing in general. (Debebe Zeleke_cooperative development office, Pos. 25-26)

There is difference in their performances among the CIGS. Jemjem-Mela is exemplary in this regard, a Dhaye-Tuti and Lencho-Borso village is considered as better performing CIGs. Conversely, there are villages which are low performing due to the dearth of follow up. For instance, Shenkora-Shesheng which participated in goat fattening and production, and Aware-Golje is the other CIG that failed; and Wale-Chilalo is also another example. Medium performing are Jamo-Berdada, Olantu-Largi, Dambaza-Wole. In areas where stakeholder and members work well, the CIG categorized as better performing ones, but the other way around happen when the stakeholders and the member could not perform their respective responsibilities. (Zeleke Hailu_Livestock and fishery development office, Pos. 16)

The respondent said they plan what they need to do but the effectiveness is not that much and it might fail. It takes much time to effectively reach the CIG teams and effectively inculcate them on board. (Teshome Tolossa_Women and youth affair office, Pos. 7)

The respondent said the applications and implementation of the CIG can be considered as a medium and despite the failures that occur most often, the members are benefiting on one way or another. Once the group members have joined the CIGs, they can engage in other livelihood activities based on the income they garner from the membership. (Teshome Tolossa_Women and youth affair office, Pos. 9)

The respondent has said the CIGs are productive in the area and particularly the improving milk and milk productivity has been enhanced the income of farmers. In the area, the CIGs are notable in milk production; and also the seed multiplication; seed multiplication including Korra and Dagim. (Admasu Kebede_DA_Lencho Borsu, Pos. 21)

However, as the time goes, the productivity of these groups have declined and later the groups were dissolved. Nevertheless, these groups benefited from the CIGs as their income and livelihood increase even of the group was dissolved because they shared the cattle population at their final time. (Tesfaye Tewabe_DA_Abo Yayambana, Pos. 29)

1. The groups expenditure

The discussant explained that the big expenditure of the group is to buy a fodder for the cattle. They say this expenditure is a way great and increasing through time as an example if the grass was 2000, birr it is 50000 birr now. The by-product of a teff which was 200 birr two years ago costs as high as 5000birr now. (FGD 1_Dairy farm_Lencho Borsu, Pos. 38)

1. The variation between their expenditure and income

These expenditure does not commensurate with the income they garner which is from  butter sale only thus far because of the problems mentioned above, particularly transportation, electricity, and fodder related problems. They said the income for the butter can be of 500 birr per month on average, but the expenditure can be 4000 birr per month. However, they said they also benefited from the group in that they were able to buy 7 more cows in the last three years. Nevertheless, the income they garner is lesser of their expectation. (FGD 1_Dairy farm_Lencho Borsu, Pos. 39)

The CIG has sold about 622 hens during their functioning time and able to garner 39500 birr in general and they shared 1975 from this output. However, the income they got did not commensurate the efforts they paid and the requirement for upbringing hens also. It takes three months for small hens to be grown fully, but the group could not feed poultry through these times because the feeding is expensive and can incur more expense than the income they generate from the production. (FGD 3_Poultry production_Wale Chilalo, Pos. 15)

**What is the SWOT of the Common Interest Groups implementation at the study area?**

1. Opportunities both for the members and local people

The discussants said they increased the input for the local market so that the local people can satisfy their demand for sheep for various purposes. They also said the local people also learned that the fattening and producing the sheep is a relevant and feasible business in the area. Furthermore, the local people now a day are forming groups and doing the same after the group owned by the respondent and his team. (FGD 2_Sheep fattening_Dhaaye Tuti, Pos. 33)

They said the nearby communities have learned the benefit poultry production can have since they used to visit the group; and they claimed that most of these visitors also established their own comparable poultry production businesses. (FGD 3_Poultry production_Wale Chilalo, Pos. 24)

Other members of the community learned a lot form the team. The villagers asked for the experience sharing platform as well. (FGD 4_Oxen fattening_Abo Yayambana, Pos. 36)

Opportunities:

1. The area is potential for cattle breeding and fattening. The AGP has proved that.

2. It enables the youth to build on their potentials and get employment opportunities. And the confirmation that anyone who works can get benefit and means of livelihoods.

The CIG has a great potential to create more opportunities if implemented well. And the respondent labels them as generally be a medium. (Zeleke Hailu_Livestock and fishery development office, Pos. 27-29)

Opportunities of CIGs:

⮚  Employment

⮚  Productivity (Teshome Tolossa_Women and youth affair office, Pos. 13-14)

New opportunities:

The respondent has said the CIGs improved the area and helped to build the already existing potentials among farmer but it is hard to count of any ground breaking new development that emerged due to the CIG or AGP. (Admasu Kebede_DA_Lencho Borsu, Pos. 29)

1. Strengths of the groups

The respondents have said that the group membership is beneficial as it enabled them by creating job opportunities as stated above. They added, it created a sort of social networking among the youths which help them build social capital on which they can depend during the hardships. They also mentioned that membership in the group do not consume all of their time and rather it provides opportunities to generate income by participating in other income generating activities. (FGD 2_Sheep fattening_Dhaaye Tuti, Pos. 29)

The discussants said working together and solidarity among the members is what they consider as the strength of the group. AGP’s initiation and the members’ willingness and ability to fill the requirements on times such as availing the saving was also considered as the other strength of the members. (FGD 3_Poultry production_Wale Chilalo, Pos. 20)

The members did not have any source of living before joining the CIG. It helped them secure some livelihoods. It also enabled them diversify their means of living. (FGD 4_Oxen fattening_Abo Yayambana, Pos. 32)

⮚  The way CIGs are organized, based on their interest, formulating proposal, and giving the inputs away is the strength.

⮚  The existing stakeholders who are supposed to help a given activities is also considered as the strength.

⮚  The initiation to develop CIGs and more or less supporting and monitoring is also the strength. (Debebe Zeleke_cooperative development office, Pos. 12-14)

1.     It created job opportunities. In a given CIG, there are 20 members. So, it can create job opportunities for 40 individuals at the kebele level.

2.     The money given by AGP, the money is a support to set an initial capital as of seal money. When they get more money, they pay the money back. (Zeleke Hailu_Livestock and fishery development office, Pos. 18-19)

The respondent has said the strength is that grouping itself helps the farmers creating an opportunity of working together and accessing the market. It helps them easily and proactively access inputs, herbicides and pesticide as well. It also helps them to get more income which they cannot accomplish individually. (Admasu Kebede_DA_Lencho Borsu, Pos. 25)

The respondent has said that the money collected from the members and the money given by the AGP is feasible enough to let them engage in the activities. He said that helps the coordination among the farmers to start the business the sooner. (Tesfaye Tewabe_DA_Abo Yayambana, Pos. 31)

The respondent has said the CIG groups helped farmers to come together and work as such which helped them build a social capital which in turn builds their capacity and togetherness. (Abera Tadesse_DA_Jemjem Mela, Pos. 20)

1. Weaknesses of the groups

It was reported that the sheep did not get that much better satisfied and well arranged protection. They said they cannot endure protecting the health of the sheep due to the conventional feeding practice they have been at it, and they added that failure hurts their effectiveness in terms of income and related benefits. As to them the treatment of the sheep is not scientific yet, and they did not monitor them carefully which is because of the huge number of sheep. (FGD 2_Sheep fattening_Dhaaye Tuti, Pos. 31)

They said the quick nature of pessimism and inability to give a recovery time was considered as a problem. They assume they should have waited for the intervention form the stakeholder before stopping the group. (FGD 3_Poultry production_Wale Chilalo, Pos. 22)

The team lacked all rounded knowledge and proactive means of securing their business. They agreed that they should have worked to build reserving place for the oxen rather than excusing it for their failure and dissolution. They also lacked that cooperation and team-orientation. (FGD 4_Oxen fattening_Abo Yayambana, Pos. 34)

The dissolution of groups is resulted from loss of interest in working together. (Bikila Tolossa_AGP coordinator, Pos. 60)

There is lack of regulation and strict laws to abide the members and it is only the ‘seal’ money that is used to control members. (Bikila Tolossa_AGP coordinator, Pos. 62)

The other problem is lack of evaluation. The activities the CIG undergo do not accompany with the evaluation and their progress is not clear. (Bikila Tolossa_AGP coordinator, Pos. 65)

⮚  CIGs are not equally beneficiaries and the members face drop outs. The failure to enhance the members to join cooperatives.

⮚  Inability to solve conflicts and failure to sustain the CIGs is also another weakness. (Debebe Zeleke_cooperative development office, Pos. 16-17)

1.     Lack of participation of local DA and the people in buying the animals.

2.     Lack of follow up and monitoring. Because the sector has a lot of tasks to do and lack of budge also rationalize the failure in this regard. In a year, two or three times, CIGs can be met and follow up. Jemjem-Mela is the most visited area and followed up most often than others. And it is also used to share experience for other farmers.

3.     Lack of ownership and coordination among the stakeholders. (Zeleke Hailu_Livestock and fishery development office, Pos. 21-23)

The CIGs do not have any controlling and punishment means that affected the effectiveness of the CIGs. (Teshome Tolossa_Women and youth affair office, Pos. 5)

Internally, CIG members fail to provide saving. There is a problem while initiating the business and providing the contributions. Frequently, conflicts rise among themselves also due to absenteeism and neglect which ended most of groups in dissolution. (Teshome Tolossa_Women and youth affair office, Pos. 8)

⮚  Lack of market linkage

⮚  Lack of proper application of plans at the local level (Teshome Tolossa_Women and youth affair office, Pos. 16-17)

The main weakness of CIGs is that they are exposed to the conflicts and quarrels; there can be disagreements and conflicts among the members. (Admasu Kebede_DA_Lencho Borsu, Pos. 27)

He said the continuity is always a problem despite the fact that the livelihoods of the members in one way or another are always improved. (Tesfaye Tewabe_DA_Abo Yayambana, Pos. 33)

The respondent has said the CIG failed to bring farmers who have common interest and objectives together. (Abera Tadesse_DA_Jemjem Mela, Pos. 22)

1. Threats the groups encountered

They said that during the first few months of the business commencement, they had to face problems like losing the sheep due to illness and related factor. And they stated that the disease is likely to even occur in the future unless some scientific interventions take place in the near future. This made them worried about their future and their business in the future. (FGD 2_Sheep fattening_Dhaaye Tuti, Pos. 35)

1. Problems the CIGs have encountered

The discussants illustrated a lot of problems that the encountered in their business:

1. There is no transportation service in their village to be able to transport the milk from their village to the nearby urban place. They said, since there is no transportation service in the village, they cannot transport the milk and this triggered them to depend on only on butter production and its sale as the major business activity.
2. The discussants have also said there is no electricity in the village to use refrigerator and keep the milk healthy for a long time until they sell it.
3. Moreover, the discussant said they have been facing problems with the inputs for the cattle (Cow and calves).They said the forage for the cattle are from the local grass and farm bi-products who costs is increasing through time. There is this type of grass called ‘*Shakke*’, but since it needs fertilizer for its production, it incurs them more expenses.
4. Finally, they said the money they received from AGP was quite small to unlock their potential and benefit from the milk production. Since the money is small, they had to buy less quality of the cows just to satisfy their need to start the business. It did not find them well their potential. They said had it not been the case, they said, they could have bought a more productive cows. (FGD 1_Dairy farm_Lencho Borsu, Pos. 29-32)

They also recall that during the beginning of the business they had lost numerous sheep for the dead. (FGD 2_Sheep fattening_Dhaaye Tuti, Pos. 20)

Besides the benefits side, however, the respondents illustrate that it was difficult to get the enough farming place for their sheep. They stated although the government promised to provide such area, it did not live up to their promise neither did materials used for construction is provided by the governmental entities. (FGD 2_Sheep fattening_Dhaaye Tuti, Pos. 23)

The group does not have enough materials to locate and transport the poultry and their products also. AGP has also given them the poultry during the rainy season but the small poultry product require warm places which the group lacks. The discussant also said they requested the AGP coordinators to provide them the material supports on time, but there was no such assistance back then. The aggregate result of these problems is the dissolution of their group and membership. (FGD 3_Poultry production_Wale Chilalo, Pos. 15)

Due to various problems they had to face including the lack of inputs for fattening purposes, they dissolved the group and share the oxen among themselves. Nevertheless, they were beneficiaries even if the group is dissolved. (FGD 4_Oxen fattening_Abo Yayambana, Pos. 26)

Perception: Although the business is perfect, the support from the government side was discouraging. They could not get service from the stakeholders; they lacked inputs and reserves where they can keep the oxen. They did not get training on the routine basis also. They assume there would have been more benefits had they followed the training appropriately. (FGD 4_Oxen fattening_Abo Yayambana, Pos. 28)

Some groups dissolved their associations. (Bikila Tolossa_AGP coordinator, Pos. 58)

The other problem is that when the farmers and graduate class is assigned to the same group, graduated students can leave in cases when they secure job in another places, hence this create a sort of conflict. The capital is also a problem. For instance by about 20 individuals, if the AGP gives them 100k birr, they can only be able to buy 3 oxen and sharing the profit by about 20 is imminent and can also be a reason for the dissolution of the groups. The budget allotted for groups is low and cannot be satisfactory among all. The buying capacity of money is also changing through time and it cannot fit the needs of the members now in comparison to the earlier times. (Bikila Tolossa_AGP coordinator, Pos. 62)

The support comes from all stakeholders like the livestock office, youth and women affairs, and cooperatives agencies. All have stake in the CIG groups. DA also working in cooperatives with the members and they give routine assistance and supports. However, the support rendered is not enough and there are yet to be done in satisfying needs. DAs also support but the main activities of buying the cattle’s and oxen excludes them and accomplished by the AGP, Cooperatives organizations, and the financial agencies of the woreda. The DAs may also consider the AGP activities as extra-work and they may not give as much attention as the activities need. (Bikila Tolossa_AGP coordinator, Pos. 64)

The other problem is lack of evaluation. The activities the CIG undergo do not accompany with the evaluation and their progress is not clear. (Bikila Tolossa_AGP coordinator, Pos. 65)

The problem is particularly how to handle the members without the dissolution. There is no guidelines that can hold the members together and how to guide if they actually dissolved. The CIG has criteria to select the members, but the one who dissolved the group is not taken in to account for their actions. (Debebe Zeleke_cooperative development office, Pos. 5)

There is a coordination lack among the stakeholders to better off CIGs. The rationale behind this is that the villages are huge and bulky which restrain stakeholder to meet all of these places. The workers at the village level are not considering the village level as their main activities and there is no evaluation in that regard that much and even if some motives exist, it is not effective as the local DAs are not showing that much dedication-they do not meet the CIG groups on the daily basis but they only report the progress merely for on the day of annual reporting and evaluation. This emanates from the fact that the local DAs mostly lack of owning the CIGs and they consider them as outsider and of NGO ownership. They assume they are working but it’s not the ultimate responsibility required from them, but there directions to work on that regard. (Debebe Zeleke_cooperative development office, Pos. 10)

The budget alloted to be given for the CIGs is limited irrespective of the fact that each CIG wants to have their share. Besides to this, each stakeholder which support the CIG has various plans to be executed mainly with money like monitoring and evaluation of these CIGs. Hence the CIGs encounters lack of follow up and monitoring. (Zeleke Hailu_Livestock and fishery development office, Pos. 25)

However, the respondent has said, there is no uniform knowledge among the stakeholders and the CIG members. (Teshome Tolossa_Women and youth affair office, Pos. 3)

The DAs are also reluctant to follow and control since they have not participated while buying the animals. (Teshome Tolossa_Women and youth affair office, Pos. 5)

The experts from the various concerened stakeholders are not providing the support they required to render to the desired level. They are reluctant to reach the CIG members and may not appear in the village and not know the members and the members may not know him also. (Teshome Tolossa_Women and youth affair office, Pos. 8)

**What intervention strategies could be designed to enhance the operation of Common Interest Group in the study area?**

1. Members perspective on the futurity of their groups

They also vowed that they are not going to dissolve the group or there is no such ambition of quitting the membership when and if people leave which did not happen yet. They also said the group will exist regardless of the capital or the risk they might face. (FGD 2_Sheep fattening_Dhaaye Tuti, Pos. 19)

The members said that they actually benefitted when the CIG is dissolved since they shared the hens and their products among themselves and owned them privately. But they do not have a positive attitude for the group membership as they face more of risks and debts than benefits. They also reiterate that there was no quarrel and disagreement among the members and the malfunctioning of the group emanated only from the risky nature of the business. (FGD 3_Poultry production_Wale Chilalo, Pos. 16)

1. The way forward to benefit from the CIGs

The respondents have said that in order to benefit more from such groups as CIG, they mentioned two strategies:

1. The CIG working areas and its components should be broadening.

2. Union should be formed from the groups and facilitate the marketing activities. (FGD 2_Sheep fattening_Dhaaye Tuti, Pos. 37-39)

The discussants have said they wanted to commence the business even now but they need support from the concerned bodies. Hence, there is a need for the prompt intervention from the government side and they said there is a need for the continuous follow up. They think had there been market linkage, they would benefit from the service at least their transportation cost can be reduced. They also underlined that the service related to poultry should be available on time during the dry season. (FGD 3_Poultry production_Wale Chilalo, Pos. 26)

What needs to be done to enhance CIGs:

1. Guideline should be owned and the members should feel they actually own the group.

2. The guide lines should be in line with time and needs to be updated, and strictly followed.

3. The guidelines should also be developed by the team members themselves. When, why, and on what precondition should the CIG group members’ leave should be clearly stated. Whether and if the profit should be shared or not also needs to considered.

4. Livestock agency, cooperatives organization, youth, women and children and agricultural office all are stakeholders that need to be taken in to account, but there is no accountability and evaluation of the progress of whatsoever. There is no budgetary limitation in this regard, if not the limitation in women, youth and children’s affairs as they depend on other organizations particularly the Cooperative agencies. The prime problem is lack of evaluation.

5.  DAs are also the sixth stakeholders and they routinely engage with the local farmers.

6. The cooperatives, finance, and youth affairs buy the animals. The DA should be involved though since they follow up the activities and they know more about the needs of the local farmers.

7. The involvement of the local village level is not considered either ways. (Bikila Tolossa_AGP coordinator, Pos. 71-77)

It needs the stakeholders to work together and they need to sustainably work with the CIGs to enhance their status and group performance. (Debebe Zeleke_cooperative development office, Pos. 19)

There is a need to involve the administrators at the woreda level. The local village administrative bodies are already doing their job but to include the local village level needs the job to be done at the woreda level. So mainstreaming the issue at the woreda level helps achieving a further development at the village level and involving the woreda administrative organs help to push the local administrations and normalize the trend of working with the local CIGs. (Debebe Zeleke_cooperative development office, Pos. 21)

What needs to be improved for better success:

1.     The guidelines related to CIG like how to buy the cattle is limited. Various stakeholder need to be involved. After the cattle is bought, the way it is followed up and the perception of member not to sell ahead of achieving the objectives is not targeted.

2.     During buying the animals’ scientists assess the type of oxen, sheep, and goats and their probability of success. The problem mostly, however, lies related to fodder which most of the CIGs fails to gather. For that reason, the fattening period can be extended to four or more months. Similarly, the poultry needs much more care.

3.     The local level DAs and village administrators monitoring and follow up is needed to achieve the better and reduce the problem by high as 50%.

4.     There is benefit with reducing the members’ size for the management purpose. The productivity also rises. When the number increases, the productivity and the profit also decline. The basic reason to have20 individual is to pool the 25% money which mostly lacked in the village. There is no intention of creating employment by increasing the number of members since there are other projects working on creating opportunities.

5.     There is a need to review the CIG practice starting from the initial stage of selecting the beneficiaries to the profitability. Every stakeholder and the members should be followed up and their level of implementing the objectives should be assessed.

6.     CIG should improve a clear working pattern, about who should be included. The roles and responsibility should be clearly put and addressed in that way.

7.     AGP helped the supply of improved seeds and increased the productivity in that regard. (Zeleke Hailu_Livestock and fishery development office, Pos. 31-37)

Ways to improve:

The respondent argues that there is a need for continues support from the experts, particularly the fodder types that can enhance the productivity of the oxen. There is need to formulate regulations as to what to happen to individuals who happen to break rule and regulations. The existing the stakeholders are enough, but particularly the local DA needs to involve better. The rural DAs should consider the AGP as their responsibility also. They lack a separate budgetary allocation for the DAs but for the woreda level stakeholders, they are getting benefits and salary. There is a need to market linkage. (Teshome Tolossa_Women and youth affair office, Pos. 11)

He recommended that the place where the animal are about to be kept, their feeding system and labor forces should be adequately prepared before forming the CIG group. (Tesfaye Tewabe_DA_Abo Yayambana, Pos. 40)
