# Supplementary material for: Effects of common interest groups on rural women and youth livelihood: A qualitative study from Central Ethiopia
Source: PLoS One. 2023 Oct 20;18(10):e0283532. doi: 10.1371/journal.pone.0283532 (PMC10588890; doi:10.1371/journal.pone.0283532)
Supplement: S5 File — (DOC) [file pone.0283532.s015.doc]

Results/Findings of the study

In this chapter, the study result is summarized into various themes developed during the data collection and analysis.

1. **An overview of the CIGs**

According to the district’s AGP II coordinator, 45 CIGs were established at the district level, of which 24 were men youth and 19 were women (Bikila Tolossa_AGP coordinator). The district’s cooperative development officer also stated that 44-46 CIGs were established during the AGP 2 period (Debebe Zeleke_cooperative development office). Zeleke, from the district’s Livestock and fishery development office, added that the intension was to establish two CIGs in every 25 rural kebeles of the district, of which one belongs to the youth men and the other belongs to the women group (Zeleke Hailu_Livestock and fishery development office). Consistent with Zeleke’s idea, Teshome, an officer from the district’s women and youth affairs office has said that 50 CIGs were planned to be established mainly in collaboration with the district’s cooperative development office, which is two in each village (Teshome Tolossa_Women and youth affair office). Bikila noted that these CIGs are engaged in various agricultural-related activities in which they predominantly engage in animal fattening like dairy farming, oxen fattening, sheep and goat rearing and fattening, and poultry production (Bikila Tolossa_AGP coordinator). Teshome also asserted that the CIGs were formed after the awareness-raising activities conducted in the villages by various stakeholders like AGP II coordination office, women and youth affair office, livestock and fishery development office, and cooperative development office (Teshome Tolossa_Women and youth affair office).

With regards to criteria of members selection into the group, the district’s AGP II coordinator has stated that in principle, would-be members need to have similar interests and should be credit-free individuals. The age also matters for forming the CIGs, the working-age (18-55) is preferred to others. Individuals from the same family members can also form a CIG together. He added, the AGP helps 75% of the initial capital while the members contribute the rest. The money given is a seed. If a member leaves a group, it's only profits that he/she takes out while leaving (Bikila Tolossa_AGP coordinator). Consistent with the coordinator’s idea, Tesfaye has said that one of the criteria for selecting the farmers for the CIG is that they had to have a common interest and initiation to work together for change. In their village, for instance, two CIGs availed and one consisted of 12 men who fatten oxen, and the other group which consisted of both men and women produce sheep (Tesfaye Tewabe_DA_Abo Yayambana kebele).

- 1. **The purpose of forming CIGs**

The purpose of forming CIGs is stated as putting together an association of individuals with a common interest who agree to work together toward a common goal with a great assumption that working together toward common goals can help people break down barriers. For instance, the study discussants have said that their main motive of forming a CIG was for upbringing the poultry and selling their products in a group arrangement so that they do it fruitfully by pooling various resources from members of their group (FGD 3_Poultry production_Wale Chilalo). Likewise, it is asserted that the CIGs are established to encourage members to enhance their livelihood which otherwise they couldn’t afford individually (Debebe Zeleke_cooperative development office).

- 1. **Processes of group formation**

With regards to the processes of group formation, discussants from FGD 1 stated that their group was formed from 15 men and 4 women totaling 19 members. When they commenced the business, they were given 90,000 ETB by the AGP, and on that, each of the members saved 1250 ETB which totals about 23,750 ETB (FGD 1_Dairy farm_Lencho Borsu). The FGD 2 discussants have also reported that the group they belong to which has 10 members started to operate shortly after their formation in 2010 E.C. It is located in Dhaaye Tuti kebele. The respondents stated that they came together and form their group based on their self-reported interest which of course supported by the awareness-raising campaign held in their village and at the woreda level as well. The group also opted to fattening and producing sheep and they reiterated that they chose the sheep-related task because of the conducive environment they have for it (FGD 2_Sheep fattening_Dhaaye Tuti).

Besides, as to the FGD 4, the group was established in 2010. First, the stakeholders from the woreda came with the village leader and talked to them. The AGP II organized individuals who happen to have similar interests and residing in the same area. The village leader gave them the list and all of the listed individuals attended the meetings, and 20 individuals came together and form the group. However, as time goes the number of a group member is minimized to 12 from 20. The discussants added that the task of oxen fattening was chosen by the interest of the group members and they took training two times. They were given 70,000 ETB after they took part in the woreda level training; and their group was established after they pool the mentioned amount of money together with the members own contribution of about 14000 ETB (FGD 4_Oxen fattening_Abo Yayambana).

The other perception area examined by the interviewer is a startup capital groups contribute and their perception on it; whether the members assume the initial money they had to contribute is expensive. Discussants of the FGD 2 asserted that the contribution was not that demanding for them but of course some members felt a bit of pressure to fulfill the expectations easily. They also said the group members knew that the contribution was meant to initiate the business and it will be saved for the future risk aversion (FGD 2_Sheep fattening_Dhaaye Tuti). The FGD 3 discussants have also said they were 20 women when they start the business in 2010, and each of them contribute 1200 ETB which they had to pay as part of the initial capital which accounts for 25% while the rest is contributed by AGP. They said the group was able to collect about 22,000 ETB and they got 66000 ETB from AGP (FGD 3_Poultry production_Wale Chilalo). Members of the FGD 4 also saved about 24% which was 14000 ETB from the total amount they invested as a startup capital for their business, and affirmed that the money they have had contributed doesn’t cost them as such and didn’t require them to exert much effort to contribute the mentioned amount of money (FGD 4_Oxen fattening_Abo Yayambana).

- 1. **How members of the groups use money up-on their groups’ formation**

On the usage of money, the discussants from FGD 1 stated that the saving from the members was used mainly to construct an abode, a place to keep the cattle (cows and calves). With the financial support from AGP, they bought 8 cattle mostly of which are cows which were meant for the diary production. The group had a total of 17 cattle, and sold two of them in the last three years (FGD 1_Dairy farm_Lencho Borsu). The respondents from FGD 2 have also said that when they commence the business, they were able to raise 1660 ETB from each of the members and supported with 50,000 ETB from AGP with which they bought 55 sheep (FGD 2_Sheep fattening_Dhaaye Tuti). Members from the FGD 3 added that their group was able to collect about 22,000 ETB and they got 66000 ETB from AGP. With that money, they said they constructed an abode for the poultry. They also said AGP has given them 1200 small poultry but many of them have died with few surviving (FGD 3_Poultry production_Wale Chilalo).

- 1. **How the groups gained the working place**

All groups the researcher have had interviewed were rented and/or use the members’ garden or land as a working place. For instance, the discussants of FGD 2 mentioned that they rented a place where they can keep their sheep. Since the sheep are many, they shared them among 5 of them as well (FGD 2_Sheep fattening_Dhaaye Tuti). Respondents of the FGD 4 were also stated that even though the program told them that they would be given the place where they can keep their animal and materials used for constructing an abode, they failed to do that. Then, the members decide to rent a place recommended by the village leader, and could not build a house for the rest (FGD 4_Oxen fattening_Abo Yayambana, Pos. 23). The district’s AGP II coordinator also avowed that even though they promised to give work places for the CIGs beneficiaries, they couldn’t able to make it real because of budget related constraints they have encountered (Bikila Tolossa_AGP coordinator).

- 1. **Groups’ participation on the processes of buying the livestock**

Only the discussants of the FGD 1 have reported that they have participated on the processes of buying the cattle they own as a group later. They said, they have participated after they had a consultation with the AGP experts on the issue that they need to buy the cattle that better adapt to the local weather condition. Then, members of the CIG and the technicians from woreda level AGP II team together bought the improved cow breed that can give a better product and adopt better to the local weather condition. This is against the indigenous cow breeds used by many farmers in the area, added the respondents. From their participation, the discussants said, they benefited as most of the cows bought are mostly of productive if not one of the cows that fail to give good milk but a calf which was sold for about 7000 birr (FGD 1_Dairy farm_Lencho Borsu).

Contrary to this, from the discussions I had with other CIGs, I came to realize that beneficiaries other than of the CIG at Lencho Borsu didn’t participate on the processes of buying their livestock. Oxen owned by the discussants of the FGD 4, for instance, were bought by the experts only from the town of Goha-Tsion, recently changed to its previous name Qarre-Goha. It was reported that merely the AGP coordinator and other few stakeholders from the woreda have had participated on the buying processes of their oxen 84000 ETB in total bought about 7 but 14000 ETB was confiscated at the woreda level, said the discussants (FGD 4_Oxen fattening_Abo Yayambana). Consistently Zeleke, an officer at woreda level, stated that when buying these animals, neither the villagers nor the local DAs take part (Zeleke Hailu_Livestock and fishery development office). Bikila stated that the process of buying the livestock excludes the respective DAs. As to him, irrespective of the support rendered by the DAs through the program’s activities in general and CIG related activities in particular, the main activities of buying the cattle’s and oxen excludes them and accomplished by the AGP coordinator, officers from the woreda’s cooperative development office and finance office (Bikila Tolossa_AGP coordinator). Teshome also affirmed that the team was formed to buy the livestock for the CIGs; and it was through this team that the livestock were bought for the CIGs. He added that the local administrators do not participate in buying these livestock because of the team’s norm which allow only the engagement of livestock and financial sector workers and/or professionals in the processes of livestock buying (Teshome Tolossa_Women and youth affair office). Nonetheless, the beneficiaries wanted to participate in the processes of buying the oxen. For instance, discussants of the FGD 4 have said that had the woreda officials allow them and their respective local administrators to participate on the livestock buying processes it would have been good (FGD 4_Oxen fattening_Abo Yayambana).

- 1. **Roles and responsibilities of the members**

With regards to members’ engagement in the groups’ activities, it is reported that each members of the groups are responsible for the groups’ activities with various degrees of engagement. Discussants from the FGD 1 avowed that each member of their group is responsible to watch after the cattle and feeding them. They said, they allocate days and time for the members who would take care for the cattle once in 19 days since they are totally 19 members. A member on his/her duty day would watch the cattle, clean their abode, and feed them. In addition, the discussants said, they have an obligation to abide the rules and regulation of their group and if there is a failure in that regard, they would face a punishment of 30 birr at the very first time, 50 birr for the second time. But it’s shown that the members genuinely adherent to their group’s norm and abide the rules and regulations. The accountant and monitory body of the group are also obligated to save their financial resource to the local bank on time and withdraw when the group interested to do so, and mostly on time. The monitoring body also follows the members and their activities, the cattle and the financial budget (FGD 1_Dairy farm_Lencho Borsu).

Likewise, as to the discussants of FGD 4, the members care for the oxen on a routine basis. Some group members are, however, reported as reluctant. The quarrel happened and they informed the woreda level stakeholders and they kicked out eight individuals from the group membership by giving their saving and the group members were minimized to twelve. Soon the members divided in to two having six members each. And later on the twelve members divided in to three groups, each group having three individuals and they kept those with themselves (FGD 4_Oxen fattening_Abo Yayambana, Pos. 24).

- 1. **Perception of the members towards working in group**

The researcher investigated the CIGs members’ perception of working in group or their membership in CIGs. Being in group entity itself has its own positive sides, say the discussants of FGD 4. Working in group gave them numerous benefits which each of them could not accomplish on their own and at ease. More importantly, it is providing a sustainable means of livelihoods and employment for the members (FGD 2_Sheep fattening_Dhaaye Tuti). On contrary to this notion, discussants of the FGD 4 avowed that it is good that in the CIG grouping members should supposed to have the same interest and resides in the same area which is good for the group’s effectiveness; however, as the number of members is increased, the effectiveness reduced because there were many ideas. As to them, had the group build from only 3-5, they could have alike idea and become more effective. But as the number increased, there would be an increasing interest among the members and the probability of having a divergent idea is wide (FGD 4_Oxen fattening_Abo Yayambana, Pos. 38).

1. **The support from stakeholders**

For the question from the researcher regarding whom they consider as stakeholders, the district’s AGP coordinator stated that in the context of their program in general and activities related to CIG in particular, stakeholders are all the individuals or groups interest in the progress and results of their overall activities. They could be from the government’s line offices, the group of young people they are working with, the wider community, the donor [even though they don’t have a close contact with them]. In view of this, he added that the support comes from all woreda level governmental stakeholders like the offices of livestock and fishery development, youth and women affairs, and cooperative development. All have stake in the operation of CIG. Development Agents (DAs) of each kebele are also working in collaboration with members of the CIGs as they give routine assistance and supports, in spite of the fact that the support rendered is not enough and there are yet to be done in satisfying needs (Bikila Tolossa_AGP coordinator).

Debebe further elucidated that the woreda’s cooperative development office takes parts as one of the stakeholders in AGP. It particularly wants to encourage the CIG members to enhance their livelihood by helping them to save, enhance their income level, and encourage them to join the local cooperatives including the saving and credit cooperatives and multi-purpose cooperatives. However, many problems exist for its implementation at its fullest sense (Debebe Zeleke_cooperative development office). Besides Zeleke mentioned WALQO [Oromia saving and credit micro-finance institution] as another stakeholder in which it works on the business plan and the members start saving which is about 25% and the rest is paid by AGP. As the CIGs engage in the livestock related activities, the role of their office [Livestock and fishery development office] as a stakeholder is also reported as of paramount. The CIGs work on fattening the cattle in order to get the product needed within three months and mostly with the support of technologies. Thus, their office checks the respective livestock’s health status and their potential to get fattened. It is after these procedures that the livestock were bought and then the CIGs are followed up for their best possible performance (Zeleke Hailu_Livestock and fishery development office). As to Teshome, the main activity of the women and youth office is creating awareness about the CIGs and its benefits so that youths and women well equipped of the benefit they would gain from forming a group and working in the same. He also added that the women and youth affair office as a stakeholder helps for the smooth communication among members of the CIGs like when there is a problem among CIG members they solve it in collaboration with other stakeholders (Teshome Tolossa_Women and youth affair office).

Concerning the support from the AGP II coordination office, the district’s AGP coordinator stated that it’s their office that coordinate the work of the mentioned stakeholders. As to him, they work on both women and youths without discerning against their gender; they encourage youths to engage in CIG, and they encourage women and men also. They do not exclusively support either of male headed households nor female headed households. They do not have a gender disaggregated support package (Bikila Tolossa_AGP coordinator). As part of a stake in the operation of CIGs, the relationship between CIGs and other comparable groups is also explained. For instance, discussants of the FGD 2 have said, although another group consisting of women exists in the village, they were not that close to them despite a rare information they had to share among themselves merely (FGD 2_Sheep fattening_Dhaaye Tuti).

1. **The situation of market linkage**

With regards to the market linkage, since the AGP aims to change the buying and selling culture from the ‘occasional’ and ‘opportunistic’ sales transactions to a more consistent sales approach that builds relationships between groups of organized farmers selling to known trading partners, the researcher intended to examine the actual market linkage situation. In this regard the discussants of FGD 1 said there is no market linkage and they sell their product-butter, on the own. They have generally sold five kilo of butter one in a week at the local market place through the usual means of selling (FGD 1_Dairy farm_Lencho Borsu). Discussants of the FGD 4 also avowed that they were told by the local government that some kind of market linkage would be created for them during their initial meeting. However, they carped that there was no such kind that has been facilitated thus far and they depend on the local market for the selling purpose. The market linkage facilitation didn’t happen yet. This happened, according to the respondents, that because the government entities, AGP II coordination office, and other CIG groups by themselves are all reluctant to do so regardless of the group’s attempts of producing more sheep as the years pass (FGD 2_Sheep fattening_Dhaaye Tuti). Likewise discussants of the FGD 4 stated that although during the training, they were told they would get more access to market, that promise never existed. And during the operation time, none of the members got service from any of the stakeholders and they were discouraged in their business (FGD 4_Oxen fattening_Abo Yayambana).

The market linkage issue has risen for the concerned stakeholders at offices in an attempt to triangulate what the beneficiaries have said. Accordingly, an interviewee from the district’s cooperative development office has stated, yes, the market linkage is needed but the CIGs are not developed enough to that level yet. As CIGs strengthened, market linkage is a very important and they need market linking agents in this regard. The respondent said the dairy cooperatives may be beneficial in this regard, but there is no that great numbers of CIGs working in dairy farming and there is limited number of them and they supply low. So, there is no motive to link them with the concerned business organizations found in other areas. But in the future, it is likely that they can arrange market linkage (Debebe Zeleke_cooperative development office).

1. **Performances of the CIGs**

The study examined the performance or effectiveness of the CIGs at the study area. In relation with this, Bikila asserted that only few of the CIGs are still operating and getting benefits; and mentioned merely a group of dairy production as an exemplary in this regard (Bikila Tolossa_AGP coordinator). Debebe also averred for the lopsided performances of the CIGs. As to him, for instance, there a high development of CIGs that sold 4 or more times like the CIG located in Jemjem-Mela kebele. The other few also sold their products once or twice and benefited from it. Other CIGs can be considered as low performing and some others were dissolved because of various reasons. In light with their plans, he considered the CIGs performance is generally of medium level or at average performance (Debebe Zeleke_cooperative development office).

Zeleke, by the same tone, avowed that there is difference in the performances among the CIGs. The CIG at Jemjem-Mela is exemplary in this regard; Dhaye-Tuti and Lencho-Borso villages are considered as better performing CIGs. Conversely, there are villages which are low performing due to the dearth of follow up. For instance, Shenkora-Shesheng and Aware-Golje CIGs which participated in goat fattening and production are low performing CIGs due to various reasons, and Wale-Chilalo CIG is the ones that reported as a failed CIG. Medium performing CIGs are Jamo-Berdada, Olantu-Largi, Dambaza-Wole. In areas where stakeholder and members work well, the CIG categorized as better performing ones, but the other way around happen when the stakeholders and the member could not perform their respective responsibilities (Zeleke Hailu_Livestock and fishery development office). Teshome also said that the applications and implementation of the CIGs in their district could be considered as a medium and despite the failures that occur most often, the members are benefiting on one way or another. Once the group members have joined the CIGs, they can engage in other livelihood activities based on the income they garner from the membership. He added that even though they attempted to execute what they plan, their effectiveness is not that much and it might sometimes fail as well. It takes much time to effectively reach the CIG teams and effectively inculcate them on board (Teshome Tolossa_Women and youth affair office). Admasu, on the other hand, has noted that CIG located at their village is notable in milk production, and stated that it is productive in their locality, and activities related to milk and milk productivity has been augmenting income of farmers in there (Admasu Kebede_DA_Lencho Borsu). Another DA, Tesfaye, commented on the performance of the Oxen fattening CIG located at his locality. As to him, the group’s was productivity was going good and remains hopeful. However, as the time goes, the productivity of these groups has declined and the group was dissolved. Nevertheless, the members benefited from the CIG as their income and livelihood increase even of the group was dissolved because they shared the cattle population up on dissolution (Tesfaye Tewabe_DA_Abo Yayambana).

Discussants of the FGDs explained their groups’ performance by raising their expenditure and income they have garnered. For instance, respondents of the FGD 1 explained that the big expenditure of the group is to buy a fodder for the cattle. They said this expenditure is a way great and increasing through time; as an example if the grass was 2000 ETB it is 5000 ETB now. The by-product of a *teff* which was 200 ETB two years ago costs as high as 500 ETB now. These expenditure does not commensurate with the income they garner which is from butter sale only thus far because of the problems mentioned above, particularly transportation, electricity, and fodder related. They added the income for the butter can be of 500 ETB per month on average, but the expenditure can be 4000 birr per month. However, they said they also benefited from the group in that they were able to buy 7 more cows in the last three years. As a result the income they supposed to have from their group’s task is highly reduced and the benefit they earn is lesser of their expectation (FGD 1_Dairy farm_Lencho Borsu).

The FGD 2 discussants have also explained that the group membership was enabled them to work collaboratively, and it was not merely for the purpose of revenue making but meant for sustaining livelihoods and generating incomes for their daily needs. They added that the business helped the members to get employed which most of them used to lack, and makes their productivity to increase through time. They mentioned that when they sold the sheep for 62,000 ETB in the first year of their formation and they shared the revenues among themselves and each member got 6000 ETB. They had to share the revenues at the time basically because, they said, they need money to buy agricultural inputs such as fertilizer. They also said, of course they are selling sheep then on but the 62,000 ETB they once got is the highest income recorded. Moreover, although initially they had 55 sheep, at the time of this interview, they have about 80 and more sheep now. Emphasizing their effectiveness, they also added that despite the income sharing they have to accomplish when they want, they said they also have unofficial amount of money with the group accountant (FGD 2_Sheep fattening_Dhaaye Tuti).

The poultry production group has sold about 622 hens during their functioning time and able to garner 39500 ETB in general and they shared 1975 ETB from this profit. However, the income they got did not commensurate the efforts they paid and the requirement for upbringing hens also. It takes three months for small hens to be grown fully, but the group could not feed poultry through these times because the feeding is expensive and can incur more expense than the income they generate from the production. Nonetheless, the discussants have affirmed that regardless of the malfunctioning of the group as it supposed to operate; they garnered benefits in terms of motivation and experience. They also said they were able to create social capital and the group served as a source of information sharing (FGD 3_Poultry production_Wale Chilalo).

The last group, oxen fattening group, wanted to participate on the buying processes at the very beginning of their group’s activity to buy more of oxen with the money they pooled together and they received from the AGP but they faced obstacles from the woreda stakeholders as they didn’t allow them to participate. The woreda people then bought 9 oxen for them. They later on divided their group in to two in which each group has 4 and 5 oxen. Although some fatten, others did not. This also created a conflict within the members. Yet, they have sold their products [the oxen] three times to the market and in the first round, they got a profit of 700-1400 ETB from each, and the second round they got 1200 ETB of profit from each. Other time, they got 1100-1600 ETB of profit. They bought 7 more oxen which were of the best quality type, and they sold them within 2 months at the then time. They divided the profit among themselves, and but the stakeholders did not recommend that. In spite of the stakeholders’ suggestion, the discussants justified that due to various problems they had to face including the lack of inputs for fattening purposes, they dissolved the group and share the oxen among themselves individually. Nevertheless, some of them were reported as beneficiaries even if the group is dissolved. For instance, chairman of the group bought an ox for 16000 ETB and later on sold it for 22,000 ETB within 6 months. Most of them, however, shifted the oxen fattening for another activities like shopping, buying inputs and ploughed lands and etc (FGD 4_Oxen fattening_Abo Yayambana).

- 1. **Strengths of the groups**

In explaining the strengths of their group, discussants of the FGD 2 have said that their group is strong because of the fact that it remains beneficial as it enabled them by creating job opportunities as stated above. They added that it is strong since it created a sort of social networking among the youths which help them build social capital on which they can depend during the hardships. The fact that being membership in the group does not consume all of their time and rather it provides opportunities to generate income by participating in other income generating activities is also mentioned as strength (FGD 2_Sheep fattening_Dhaaye Tuti). Besides, discussants of the FGD 3 have said working together and solidarity among the members is what they consider as the strength of the group. AGP’s initiation and the members’ willingness and ability to fill the requirements on times such as availing the saving was also considered as the other strength of the members (FGD 3_Poultry production_Wale Chilalo). Discussants of the FGD 4 added that beneficiaries of their group did not have any source of living before joining the CIG. It helped them secure some livelihoods. It also enabled them diversify their means of living (FGD 4_Oxen fattening_Abo Yayambana).

The way CIGs are organized [the initiation to develop CIGs]; based on their interest, formulating proposal, and making them to pool resources and inputs so as to develop sense of belongingness, the presence of relevant stakeholders that are supposed to help a given activities and the support and monitoring offered [though minimal] are all mentioned as the strengths (Debebe Zeleke_cooperative development office). In addition, Zeleke elaborated that arrangement of the CIG has created job opportunities. As to him, for instance, in a given CIG, there are 20 members. So, it can create job opportunities for 40 individuals at the kebele level. He added that the money given by the AGP as seed money to set an initial capital is one of the arrangement’s strength because when they get more money they can start what they want to (Zeleke Hailu_Livestock and fishery development office). Tefaye added on the seed money that the money collected from the members and the money given by the AGP is feasible enough to let them engage in the activity which in turn enables the coordination among the farmers to start the business the sooner (Tesfaye Tewabe_DA_Abo Yayambana).

Admassu has stated that the strength is that working in group helps the farmers creating an opportunity of working together and accessing the market. It helps them easily and proactively access inputs, herbicides and pesticide as well. It also helps them to get more income which they cannot accomplish individually (Admasu Kebede_DA_Lencho Borsu). Abera further explained that the CIG groups helped farmers to come together and work as such which helped them build a social capital which in turn builds their capacity and togetherness (Abera Tadesse_DA_Jemjem Mela).

- 1. **Weaknesses of the groups**

With regards to the weaknesses of the CIG, discussants of the FGD 2 reported that the sheep did not get that much better satisfied and well arranged protection. They said they cannot endure protecting the health of the sheep due to the conventional feeding practice they have been at it, and they added that failure hurts their effectiveness in terms of income and related benefits. As to them the treatment of the sheep is not scientific yet, and they did not monitor them carefully which is because of the huge number of sheep (FGD 2_Sheep fattening_Dhaaye Tuti). Discussants of the FGD 3 also mentioned the prevalent pessimism among the group members and inability to give a recovery time among the stakeholders as weaknesses. (FGD 3_Poultry production_Wale Chilalo). Besides, respondents of the FGD 4 stated that their group lacked all rounded knowledge and proactive means of securing their business. They asserted that they should have worked to build reserving place for the oxen rather than excusing it for their failure and dissolution. They also lacked that cooperation and team-orientation. Although the business is perfect, the support from the government side was reported as discouraging. They could not get service from the stakeholders; they lacked inputs and reserves where they can keep the oxen. They did not get training on the routine basis also. They assume there would have been more benefits had they followed the training appropriately (FGD 4_Oxen fattening_Abo Yayambana).

The district’s AGP coordinator affirmed that the dissolution of groups is resulted from loss of interest in working together. He added that there is lack of regulation and strict laws to abide the members and it is only the ‘seal’ money that is used to control members. As to him another weakness of the CIGs is lack of evaluation; in which it is indicated with the fact that the activities the CIG undergo do not accompany with the evaluation and their progress is not clear He further explained that even though DAs are in charge of facilitating the main activities of the CIGs, buying the cattle’s and oxen excludes them, to be accomplished by the AGP coordinator [i.e. by the respondent himself], an officer from the district’s cooperative development office and an officer from the same district’s finance office. This in turn, as to him, makes the DAs to feel and consider the AGP activities as extra-work and they may not give as much attention as the activities need (Bikila Tolossa_AGP coordinator).

Debebe also avowed that members of the CIGs are not equally responsible and beneficiaries which in turn remains a fertile ground for members drop outs. He also mentioned inability to solve conflicts and failure to sustain the CIGs as another weakness; and further elucidated that the woreda’s cooperative development office help saving, enhance their income level, and encourage them to join the local cooperatives including the saving and credit cooperatives and multi-purpose cooperatives. However, many problems exist for its implementation at its fullest sense. This is because the members are mostly of poor. The CIG is based on the monthly payment which they cannot and the cooperatives also require them buying sharing during the time of joining one them. However, those better off individuals are joining the cooperatives. The poor does not fit these expectations. In addition, the conflicts most often rise among the members and the way they are organized was a problem for the later conflict among the members (Debebe Zeleke_cooperative development office).

Zeleke on the other hand mentioned lack of participation of local DA and the beneficiaries in buying of the livestock, lack of sense of ownership and coordination among the stakeholders and lack of follow up and monitoring as weaknesses. Stakeholders of the CIGs merely meet up to do a follow up two or three times per year. Jemjem-Mela is the most visited area and followed up most often than others. And it was also used to share experience for other farmers. For the failure related to follow up and monitoring, the respondent has rationalized that their office has a lot of tasks which couldn’t allowed them to engaged in monitoring related stuffs (Zeleke Hailu_Livestock and fishery development office). In consistent with Zeleke’s notion, Teshome stated that the CIGs do not have any controlling and punishment means that negatively affected the effectiveness of the CIGs. As to him, internally, CIG members fail to provide saving. There is a problem while initiating the business and providing the contributions. Frequently, conflicts rise among themselves also due to absenteeism and neglect which ended most of groups in dissolution. He also added lack of market linkage and lack of proper application of plans at the local level as the weaknesses that the CIGs operating at their district encounters. The respondent also affirmed for the non-existent of uniform knowledge among the stakeholders and the CIG members (Teshome Tolossa_Women and youth affair office). Besides, Admasu asserted that the main weakness of CIGs is that they are exposed to the conflicts and quarrels; there can be disagreements and conflicts among the members (Admasu Kebede_DA_Lencho Borsu). Tesfaye has also avowed that the continuity is always a problem despite the fact that the livelihoods of the members in one way or another are always improved (Tesfaye Tewabe_DA_Abo Yayambana). Abera added that the CIG failed to bring farmers who have common interest and objectives together (Abera Tadesse_DA_Jemjem Mela).

- 1. **Opportunities both for the members and local people**

In addition to the benefits the CIGs renders for the immediate beneficiaries (i.e. their members), the respondents stated for the scheme’s advantages or opportunities for the local people where it operates. Zeleke, for instance, stated that it [the CIG scheme] has proved that the area is potential for cattle breeding and fattening. Besides, it enables the youth to build on their potentials and get employment opportunities. It also remains the confirmation that anyone who works can get benefit and means of livelihoods. He added that the CIG has a great potential to create more opportunities if implemented well. Thus, the respondent labels the implementation of CIG scheme as generally be at an average level (Zeleke Hailu_Livestock and fishery development office). Teshome mentioned employment and productivity as well as Opportunities that brought by the CIG arrangement (Teshome Tolossa_Women and youth affair office). Consistently Likewise Admasu has said the CIG scheme improved the area and helped to build the already existing potentials among farmers and improved their livelihood somehow; however stated as it is hard to count of any ground breaking new development that come due to the CIG scheme (Admasu Kebede_DA_Lencho Borsu).

In consistent with what the officers mentioned the FGD 2discussants stated that they increased the accessibility sheep produces in the local market so that the local people can satisfy their demand for sheep for various purposes. They also said the local people also learned that the fattening and producing the sheep is a relevant and feasible business in the area. Furthermore, the local people now a day are forming groups and doing the same after the group owned by the respondent and his team (FGD 2_Sheep fattening_Dhaaye Tuti). Discussants of the FGD 3 also said that the nearby communities have learned the benefit poultry production can have since they used to visit the group; and they claimed that most of these visitors also established their own comparable poultry production businesses (FGD 3_Poultry production_Wale Chilalo). Discussants of the FGD 4 asserted other members of the community learned a lot form the team. They added that villagers asked for the experience sharing platform as well. In response to the role they played for their community, they were supported by then local people; some villagers even gave the group a grazing place for the member and encouragement, said the discussants (FGD 4_Oxen fattening_Abo Yayambana).

- 1. **Threats the CIGs have encountered**

With regards to the problems/threats the CIG have encountered, Bikila underscored that the support to be rendered from all the CIG scheme stakes like the livestock office, youth and women affairs, and cooperatives agencies and DAs working in the respective kebeles is not enough and there are yet to be done in satisfying needs. For instance, the main activities of buying the cattle’s and oxen are accomplished by the district’s AGP coordinator, finance and cooperative development office; and excludes the concerning DAs. Some DAs also consider the AGP activities as extra-work and they don’t give as much attention as the activities need. Another problem mentioned by the respondent is lack of evaluation. The activities the CIG undergo do not accompany with the evaluation and their progress is not clear (Bikila Tolossa_AGP coordinator, Pos. 40). Teshome added that the DAs are also reluctant to follow and control since they have not participated while buying the animals (Teshome Tolossa_Women and youth affair office, Pos. 5). Debebe further elucidated the absence of coordinator among the stakeholders to better off CIGs. The rationale behind this is that the villages are huge and bulky which restrain stakeholder to meet all of these places. The workers at the village level are not considering the village level as their main activities and there is no evaluation in that regard that much and even if some motives exist, it is not effective as the local DAs are not showing that much dedication-they do not meet the CIG groups on the daily basis but they only report the progress merely for on the day of annual reporting and evaluation. This emanates from the fact that the local DAs mostly lack of owning the CIGs and they consider them as outsider and of NGO ownership. They assume they are working but it’s not the ultimate responsibility required from them, but there directions to work on that regard (Debebe Zeleke_cooperative development office). Teshome also added the non-existence of uniform knowledge among the stakeholders and the CIG members. As to him, the experts from the various concerned stakeholders are not providing the support they required to render to the desired level. They are reluctant to reach the CIG members and may not appear in the village and not know the members and the members may not know him also (Teshome Tolossa_Women and youth affair office).

Bikila added that lack of homogeneity of the group members as another problem. As an indicator, when the farmers and graduate youths is assigned to the same group, graduate youths can leave in cases when they secure job in another places, hence this create a sort of conflict. Because of this some CIGs dissolved their groups. Lack of adequate startup capital is also mentioned as a problem. The budget allotted for groups is low and cannot be satisfactory among all. For instance, the program gave 100 thousand for about 20 individuals, in which they can only able to buy three oxen; and sharing the profit by about 20 is imminent and also be a reason for the dissolution of some groups. The problem is exacerbated as the buying capacity of money is changing through time and it cannot fit the needs of the members now in comparison to the earlier times (Bikila Tolossa_AGP coordinator). Likewise Zeleke stated that the budget allotted to be given for the CIGs is limited irrespective of the fact that each CIG wants to have their share. Besides to this, each stakeholder which supports the CIG has various plans to be executed mainly with money like monitoring and evaluation of these CIGs. Hence the CIGs encounters lack of follow up and monitoring as well (Zeleke Hailu_Livestock and fishery development office).

Debebe on the other hand mentioned absence of a guideline as a main threat/problem of the CIG’s scheme. As to him, there is no guideline that can hold the members together and how to operate, even what to do at times of dissolution. The CIG has criteria to select the members, but the one who dissolved the group is not taken in to account for their actions (Debebe Zeleke_cooperative development office).

Likewise the aforementioned officers, discussants stated a lot of problems that they encountered in their CIG based businesses. For instance, FGD 1 discussants mentioned the non-existence of transportation service in their village to be able to transport the milk from their village to the nearby urban place. They said, since there is no transportation service in the village, they cannot transport the milk and this triggered them to depend on only on butter production and its sale as the major business activity. They have also affirmed that there is no electricity in the village to use refrigerator and keep the milk healthy for a long time until they sell it. Moreover, the discussant said they have been facing problems with the inputs for the cattle (cows and calves). They said the forage for the cattle are from the local grass and farm bi-products who costs is increasing through time. There is this type of grass called ‘*Shakke*’, but since it needs fertilizer for its production, it incurs them more expenses. They added that the money they received from AGP was quite small to unlock their potential and benefit from the milk production. Since the money is small, they had to buy less quality of the cows just to satisfy their need to start the business. It did not find them well their potential. Had it not been the case, they said, they could have bought a more productive cows (FGD 1_Dairy farm_Lencho Borsu). The FGD 2 discussants also recall that during the beginning of the business they had lost numerous sheep for the dead. As to them it was difficult to get the enough farming place for their sheep. They stated although the government promised to provide such area, it did not live up to their promise neither did materials used for construction is provided by the governmental entities. The added that during the first few months of the business commencement, they had to face problems like losing the sheep due to illness and related factor. And they stated that the disease is likely to even occur in the future unless some scientific interventions take place in the near future. This made them worried about their future and their business in the future (FGD 2_Sheep fattening_Dhaaye Tuti).

Besides the FGD discussants stated that their group does not have enough materials to locate and transport the poultry and their products also. AGP has also given them the poultry during the rainy season but the small poultry product require warm places which the group lacks. The discussant also said they requested the AGP coordinators to provide them the material supports on time, but there was no such assistance back then. The aggregate result of these problems is the dissolution of their group and membership (FGD 3_Poultry production_Wale Chilalo). Due to various problems they had to face including the lacks of inputs for fattening purposes, discussants of the FGD 4 have said that they dissolved the group and share the oxen among themselves. As to them, although the business seems perfect in that they would have remain beneficiaries had the problems they encountered were solved and had they followed the training appropriately, the support from the government side was discouraging, they could not get service from the stakeholders, they lacked inputs and reserves where they can keep the oxen and they did not get training on the routine basis as well. Nevertheless, it is asserted that they were beneficiaries even if the group is dissolved (FGD 4_Oxen fattening_Abo Yayambana).

1. **The way forward to benefit from the CIGs**

With regards to what needs to be done to enhance performances of the CIGs, Bikila, the district’s AGP coordinator, have first mentioned that guideline of the group should be developed by the members and the members should feel they actually own the group. As to him, would be the guide lines should be in line with time and needs to be updated, and strictly followed. The guidelines should also be developed by the team members themselves. When, why, and on what precondition should the CIG group members’ leave should be clearly stated. Whether and if the profit should be shared or not also needs to considered. Livestock agency, cooperatives organization, youth, women and children and agricultural office all are stakeholders that need to be taken in to account, but there is no accountability and evaluation of the progress of whatsoever. There is no budgetary limitation in this regard, if not the limitation in women, youth and children’s affairs as they depend on other organizations particularly the cooperative development office. The DA should be involved in the processes of buying livestock since they follow up the activities and they know more about the needs of the local farmers. The involvement of the local village level is not considered either ways. Monitoring and evaluation should also be strengthened by these stakeholders (Bikila Tolossa_AGP coordinator).

Debebe also asserted that it needs the stakeholders to work together and they need to sustainably work with the CIGs to enhance their status and group performance. He added there is a need to involve the administrators at the woreda level. The local village administrative bodies are already doing their job but to include the local village level needs the job to be done at the woreda level. So mainstreaming the issue at the woreda level helps achieving a further development at the village level and involving the woreda administrative organs help to push the local administrations and normalize the trend of working with the local CIGs (Debebe Zeleke_cooperative development office).

Furthermore Zeleke mentioned what needs to be improved for better success of the CIGs at their locality like guidelines on how the CIGs operate like how to buy the cattle should set out by the respective members. After the cattle and their fodder are bought various stakeholders need to be participating and involved on how they should be monitored. In relation to this, it is stated that during buying the livestock the concerned professionals assess the type of oxen, sheep, and goats and their probability of success. The problem mostly, however, lies related to fodder which most of the CIGs fails to gather. For that reason, the fattening period can be extended to four or more months. Similarly, the poultry needs much more care. He added that the local level DAs and village administrators monitoring and follow up is needed to achieve the better and reduce the problem by high as 50%. There is benefit with reducing the members’ size for the management purpose. The productivity also rises. When the number increases, the productivity and the profit also decline. The basic reason to have 20 individual is to pool the 25% money which mostly lacked in the village. There is no intention of creating employment by increasing the number of members since there are other projects working on creating opportunities. There is a need to review the CIGs practice starting from the initial stage of selecting the beneficiaries to the profitability. Every stakeholder and the members should be followed up and their level of implementing the objectives should be assessed. CIG should improve a clear working pattern, about who should be included. The roles and responsibility should be clearly put and addressed in that way. AGP helped the supply of improved seeds and increased the productivity in that regard (Zeleke Hailu_Livestock and fishery development office).

Again on ways to improve the performance of CIGs at the study area, Teshome avowed that there is a need for continues support from the experts, particularly the fodder types that can enhance the productivity of the oxen. There is need to formulate regulations as to what to happen to individuals who happen to break rule and regulations. The existing the stakeholders are enough, but particularly the local DA needs to involve better. The rural DAs should consider the AGP as their responsibility also. They lack a separate budgetary allocation for the DAs but for the woreda level stakeholders, they are getting benefits and salary. There is a need to market linkage (Teshome Tolossa_Women and youth affair office). Consistently, Tesfaye recommended that the place where the animal are about to be kept, their feeding system and labor forces should be adequately prepared before forming the CIG group (Tesfaye Tewabe_DA_Abo Yayambana).

Discussants of the FGD 2 have also said that in order to benefit more from such groups as CIG, they mentioned two strategies: one is the CIGs working areas and its components should be broadening, and the second is union should be formed from the groups and facilitate the marketing activities (FGD 2_Sheep fattening_Dhaaye Tuti). Discussant of the FGD 3 have also asserted that wanted to commence the business even now but they need support from the concerned bodies. Hence, there is a need for the prompt intervention from the government side and they said there is a need for the continuous follow up. They think had there been market linkage, they would benefit from the service at least their transportation cost can be reduced. They also underlined that the service related to poultry should be available on time during the dry season (FGD 3_Poultry production_Wale Chilalo).

- 1. **Members’ perspective on the futurity of their groups**

Discussants from the FGD 2 affirmed that they are not going to dissolve the group or there is no such ambition of quitting the membership when and if people leave which did not happen yet. They also said the group will exist regardless of the capital or the risk they might face (FGD 2_Sheep fattening_Dhaaye Tuti). Discussants of the FGD 3 added that they actually benefitted when the CIG is dissolved since they shared the hens and their products among themselves and owned them privately. But they do not have a positive attitude for the group membership as they face more of risks and debts than benefits. They also reiterate that there was no quarrel and disagreement among the members and the malfunctioning of the group emanated only from the risky nature of the business (FGD 3_Poultry production_Wale Chilalo). Consistently respondents of the FGD 4 said the number of group should be reduced and each of the group has to get to know one another and work as such. The people who builds a group should be those who live in the same residence area, know one another and their effectiveness and all of that and they need to select who should join and not. So, he said, when they are formed they should be from the same village and each of the members’ effectiveness and background should be assessed. The government should also monitor and follow the group per week or a month. As of the respondent, there was no such monitoring activity from the government side. The governmental stakeholder did not clearly and carefully control the group’s works. Follow up should be aligned with controlling behaviors and punishments as well. The government should not be reluctant in that regard (FGD 4_Oxen fattening_Abo Yayambana).
