# Supplementary material for: Effects of common interest groups on rural women and youth livelihood: A qualitative study from Central Ethiopia
Source: PLoS One. 2023 Oct 20;18(10):e0283532. doi: 10.1371/journal.pone.0283532 (PMC10588890; doi:10.1371/journal.pone.0283532)
Supplement: S6 File — (DOCX) [file pone.0283532.s016.docx]

# **FINDINGS OF THE STUDY**

## Characteristics of the study participants

### KII participants profile

As indicated in table 1, a total of eleven participants were involved in the study as KIIs. The first four were stakeholders from the study area at the district level (i.e. one coordinator and three officers); the next four were agricultural professionals of the four study kebeles where the actual data was gathered; and the last three participants were from the concerned federal, regional and zonal offices. It is indicated that to maintain the principle of beneficence, the names of the study participants should remain anonymous (R. Allen & Wiles, 2016; Saunders et al., 2015). Thus, Pseudonyms are used in this particular study, to effectively portray the participants' story, maintain the human element, and make the data more reflective of real life.

Table 1: Description of the study participants; KII’s profile

| **Serial number** | **Pseudonym** | **Sex** | **Their office** |
| --- | --- | --- | --- |
| 1 | Bekele Tolossa | M | Wara-Jarso district’s AGP II coordination office |
| 2 | Fitsum Tsegaye | M | Wara-Jarso district’s Women and youth empowerment office |
| 3 | Seyoum Mekonnen | M | Wara-Jarso district’s cooperative establishment and development office |
| 4 | Zerihun Hailu | M | Wara-Jarso district’s Livestock and fishery development office |
| 5 | Fasil Kebede | M | DA at Lencho-Borsu kebele |
| 6 | Bereket Mitiku | M | DA at Dhaye-Tuti kebele |
| 7 | Firehiwot Leta | M | DA at Wale-Chilalo kebele |
| 8 | Tiruneh Awugichew | M | DA at Abo-Yayambana kebele |
| 9 | Mohammed Seid | M | Technical Advisor of the AGP II at the Ministry of Agriculture |
| 10 | Edosa Chimdi | M | AGP II monitoring expert at Oromia regional government |
| 11 | Ayelech Nigatu | F | AGP II facilitator at North Shoa zone, Oromia regional government |

### FGD participants profile

Table 2 shows the general profile of the FGDs participants; their groups' number, the types of activities they engaged in, the actual number of their members, and the number of discussants who participated during the discussion.

Table 2: Description of the study participants; FGDs profile

| **S. N^o^** | **Group number** | **Business type** | **Location**  **[***Kebele***]** | **Members number** | **FGD participants** |
| --- | --- | --- | --- | --- | --- |
| 1 | FGD 1 | *Dairy farm* | *Lencho-Borsu* | 19 | 12 |
| 2 | FGD 2 | *Sheep fattening* | *Dhaye-Tuti* | 12 | 10 |
| 3 | FGD 3 | *Poultry production* | *Wale-Chilalo* | 16 | 7 |
| 4 | FGD 4 | *Oxen fattening* | *Abo-Yayambana* | 12 | 8 |

Having described the participants and discussants, the study's findings are summarized into various themes developed during the data collection and analysis. Given that, the subsequent data structure model displayed in table 3 illustrates the way data was developed from interviews to initial codes, to second-order categories, and the aggregate dimension. The aggregated dimensions are then framed into themes to be presented in the finding part as follows:

Table 3: Flow of data from first-order categories to aggregate dimensions

| **First-order categories** | **Second-order themes** | **Aggregate dimensions** |
| --- | --- | --- |
| An overview of the CIGs | An overview of the CIGs | **An overview of the CIGs** |
| Support from the AGP II coordination office | The purpose of forming CIGs | The purpose of forming CIGs |
| The CIGs and the local people other than its members | Processes of group formation | Processes of group formation |
| Members' engagement in the group activities | How the groups gained the working place | How members use the money of their groups |
| The purpose of forming CIGs | Members' engagement in the group activities | How the groups gained the working place |
| The way forward to benefit from the CIGs | Roles and responsibilities of the members | Groups' participation in the livestock procurement processes |
| Threats the groups encountered | Perception of the members towards working in a group | Roles and responsibilities of the members |
| Opportunities both for the members and local people | Support gained from stakeholders | Perception of the members towards working in  group |
| Weaknesses of the groups | Support from the AGP II coordination office | **The support gained from stakeholders** |
| Strengths of the groups | The relationship between CIGs and other comparable groups | **The situation of market linkage** |
| Startup capital groups contribute, and their perception | Groups' use of money up-on their formation | **Performance of the CIGs** |
| The relationship between CIGs and other comparable groups | Startup capital groups contribute, and their perception | Strengths of the groups |
| Perception of the members towards working in a group | Groups' participation in the livestock procurement | Weaknesses of the groups |
| Members' perspectives on the futurity of their group | The groups' expenditure | Opportunities both for the members and local people |
| The benefits members gained | The variation between their expenditure and income | Threats the groups encountered |
| How the groups gained the working place | The benefits members gained | **The way forward to benefit from the CIGs** |
| The situation of market linkage | Performance of the CIGs | Members' perspectives on the futurity of their groups |
| The variation between their expenditure and income | The CIGs and the local people other than its members |  |
| The groups' expenditure | The situation of market linkage |  |
| Roles and responsibilities of the members | Threats the groups encountered |  |
| Problems the CIGs have encountered | Problems the CIGs have encountered |  |
| Groups' participation in the procurement of the livestock | Opportunities both for the members and local people |  |
| How members of the groups use money up-on their groups' formation | Weaknesses of the groups |  |
| Processes of group formation | Strengths of the groups |  |
| Performance or effectiveness of the CIGs | The way forward to benefit from the CIGs |  |
| The support from stakeholders | Members' perspectives on the futurity of their groups |  |

## An overview of the CIGs

Out of the 44 CIGs established in the study area, 24 were run by youth men and 19 were run by women (Wara-Jarso woreda’s AGP II coordination office, 2017). The district’s cooperative development officer also stated that 44-46 CIGs were established during the time of AGP II (M. Seyoum, personal communication, October 14, 2020). Zerihun, from the district’s Livestock and fishery development office, added that their very intention was to establish two CIGs in every 25 rural kebeles of the district, of which one belongs to the youth men and the other belongs to the women (H. Zerihun, personal communication, October 15, 2020). Consistent with Zerihun’s idea, Fitsum, an officer at the district’s women and youth affairs office has stated that 50 CIGs were planned to be established largely in collaboration with the district’s cooperative development office, which is two in each kebele (T. Fitsum, personal communication, October 13, 2020). Bikila, the district’s AGP II coordinator, also noted that these CIGs are engaged in various agriculture-related activities in which they predominantly engage in animal fattening like dairy farming, oxen fattening, sheep and goat rearing and fattening, and poultry production (T. Bikila, personal communication, October 11, 2020). Concerning their establishment, Fitsum further asserted that:

*“…the CIGs were formed after the awareness-raising activities were conducted in our kebeles by the district level stakeholders including AGP II coordination office, women and youth affair office, livestock and fishery development office, and cooperative development office…”* (T. Fitsum, personal communication, October 13, 2020).

With regards to membership criteria, Bikila has stated that in principle, would-be members need to have similar interests and should be credit-free individuals; the age also matters for forming the CIGs, the working-age (18-55) is preferred to others; and individuals from the same family members can also form a CIG together. He added that the AGP helps 75% of the initial capital while the members contribute the rest. The money given is a seed. If a member leaves a group, it's only profits that he/she takes out (T. Bikila, personal communication, October 11, 2020). Consistent with Bikila’s idea, Firehiwot, Bereket, and Truneh have stated that one of the criteria for selecting the farmers for the CIG is that they should have a common interest and initiation to work together for change (M. Bereket, personal communication, October 22, 2020; L. Frehiwet, personal communication, October 26, 2020; A. Truneh, personal communication, October 28, 2020). As an example, Truneh stated that:

*“…basing the interest they have shown in common, two CIGs were established in our kebele, one consisted of 12 men who fatten oxen, and the other group which consisted of both men and women engaged in sheep production…”* (A. Truneh, personal communication, October 28, 2020).

### The purpose of forming CIGs

The study has found that the purpose of forming CIGs is putting together an association of individuals with a common interest who agree to work together toward a common goal with a conjecture that working together toward common goals can help people break down barriers (N. Ayelech, personal communication, October 17, 2020; T. Bikila, personal communication, October 11, 2020; C. Edosa, personal communication, October 11, 2020; M. Seid, personal communication, October 2, 2020). This is consistent with the notion of the FGD participants. For instance, one (F) of the FGD 3 discussants has stated:

*“…our main motive of forming a group was to do poultry production and sell both chicken and eggs. We opted to engage in such activity through a group arrangement to pool various resources from members and to enable members to contribute what they are good at. This would in turn positively contribute to the fruitfulness of our business…”*

(FGD 3, personal communication, October 29, 2020)

(FGD 3, personal communication, October 29, 2020).

Likewise, it is affirmed that the CIGs are established to encourage members to enhance their livelihood which otherwise they couldn’t afford individually (Seyoum, personal communication, October 14, 2020).

### Processes of group formation

With regards to the processes of group formation, discussants from FGD 1 stated that their group was formed of 15 men and 4 women members. The start-up capital of this CIG was a 90,000 ETB support from AGP and 23,750 ETB members contribution (each member saving 1250 ETB) (FGD 1, personal communication, October 27, 2020) Participants of the FGD 2 have also reported that their CIG was established in 2010 E.C with ten members. It is located in Dhaye Tuti kebele. They stated that the awareness creation campaign conducted in their kebele and woreda has helped them to establish CIGs based on their full consent. The type of farm activities they pursue was also chosen by the members as their priority livelihood. They reportedly came together to pursue fattening as a livelihood source due to the presence of a conducive environment for it (FGD 2, personal communication, October 28, 2020). As to the FGD 4 discussants, their group was established in 2010. First, the stakeholders from the woreda came with the kebele leader and talked to them. The AGP II mobilized individuals who reside in the same area and have similar interests. The kebele leader then gave the list of individuals who have attended the meetings for the AGP personnel. It was in this way that 20 individuals came together and form the group. However, as time goes the number of group members is minimized to 12 from 20 because of various reasons (both individual and institutional). The discussants added that they took training twice at their woreda and the job of oxen fattening was chosen by themselves. Upon the establishment of their group, they were given 70,000 ETB and pooled an additional 14000 ETB from members (FGD 4, personal communication, October 30, 2020).

The other perception area examined by the interviewer is startup capital beneficiaries contribute and their perception of it; whether the members assume the initial money they had to contribute is expensive. One (M) of the FGD 2 discussants has stated that:

*“…the contribution was not that demanding for us but of course, some members felt a bit of pressure to fulfill the expectations easily. Members of our group knew that the contribution was meant to initiate the business and it will be saved for the future risk aversion…”* (FGD 2, personal communication, October 28, 2020).

The FGD 3 discussants have also said they were 20 women when they start the business in 2010, and each of them contributed 1200 ETB as part of the start-up capital which accounts for 25% of the required while the rest is contributed by AGP (FGD 3, personal communication, October 29, 2020). Besides, participants of the FGD 4 stated that they have saved about 24% of the total amount they invested as startup capital for their business, which was 14,000 ETB; and averred that the money they have had contributed doesn’t cost them as such and it didn’t require them to exert much effort (FGD 4, personal communication, October 30, 2020).

### How do CIGs use the money they collected upon their establishment?

On the usage of money, FGD 1 participants have stated that the saving from the members was used mainly to construct a barn, a place to keep the cattle (cows and calves). With the financial support from AGP, they bought 8 cattle mostly of which are cows that were meant for dairy production. The group had a total of 17 cattle and sold two of them in the last three years

(FGD 1, personal communication, October 27, 2020). FGD 2 discussants have also asserted that when they commence the business, they were able to raise 1660 ETB from each member and support 50,000 ETB from AGP with which they bought 55 sheep (FGD 2, personal communication, October 28, 2020, p. 2). FGD 3 participants added that they were able to pool about 22,000 ETB and 66,000 ETB from AGP; it was with this money that they constructed a barn for the poultry. They added that AGP has given them 1200 small poultry but many of them have died with few surviving (FGD 3, personal communication, October 29, 2020).

### How the groups gained the working place

The interviewed groups rented and/or use the members’ garden or land as a working place. For instance, the discussants of FGD 2 stated that they rented a place where they keep their sheep. Since the sheep are many, they shared them (FGD 2, personal communication, October 28, 2020). Participants of the FGD 4 have also stated that even though the program told them that they would be given a place where they can keep their oxen and materials used for constructing an abode, they failed to do that. Basing this the group has decided to rent a working place as per the recommendation by their kebele leader (FGD 4, personal communication, October 30, 2020). Bikila also affirmed that:

*"…even though we promised to give workplaces for the CIGs, we couldn't able to realize because of budget-related constraints we have encountered…”* (T. Bikila, personal communication, October 11, 2020).

### Groups' participation in the procurement of the livestock

Only the discussants of the FGD 1 have reported that they have participated in the processes of buying the cattle they own as a group. They participated after they had a consultation with the AGP experts as they need to buy the cattle that better adapt to the local weather condition. It was after this requirement that members of the CIG and together with a team of concerned professionals from woreda level AGP II bought the improved cow breed which can give a better product and adapt better to their local environment. They did this in a fear of not buying the indigenous cow breeds they used to own. From their participation, the discussants stated, they benefited as most of the cows bought are productive, if not one of the cows that fail to give good milk but a calf which was sold for about 7000 ETB (FGD 1, personal communication, October 27, 2020).

Contrary to the stated notion, it was found that the group other than the CIG at Lencho-Borsu didn't participate in the processes of buying their livestock. Oxen owned by the discussants of the FGD 4, for instance, was merely bought by a team of experts from the nearby market. With this, the AGP coordinator and other few stakeholders from the woreda have participated in the buying processes of their oxen. 84000 ETB in total bought about 7 oxen but 14000 ETB was seized at the woreda level, said the discussants (FGD 4, personal communication, October 30, 2020). Consistent with what they have said, Zerihun, stated that when buying these animals, neither the villagers nor the local DAs take part (H. Zerihun, personal communication, October 15, 2020). Bikila narrates his view on the processes of buying the livestock as follows:

*“…the process of buying the livestock excludes the respective DAs. Irrespective of the support rendered by the DAs through the program’s activities in general and CIG related activities in particular, the main activities of procuring the cattle and oxen exclude them and conducted by me as an AGP coordinator, officers from our woreda’s cooperative development office and finance office…”* (T. Bikila, personal communication, October 11, 2020).

The other officer, Fitsum, added:

*"…the team was formed to buy the livestock for the CIGs; it was through this team that the livestock was bought for the CIGs. The local administrators do not participate in the procurement of this livestock because of the team's norm which allows only the engagement of livestock and financial sector workers and/or livestock experts…”* (T. Fitsum, personal communication, October 13, 2020).

Even so, the CIG members have shown their interest to participate in the procurement of the oxen. For instance, discussants of the FGD 4 have said that had the woreda officials allowed them and their local administrators to participate in the procurement processes it would have been good (FGD 4, personal communication, October 30, 2020).

### Roles and responsibilities of the members

With regards to members' engagement in the group's activities, it is reported that each member of the group is responsible for the group's activities with various degrees of engagement. FGD 1 discussants avowed that each member of their group is responsible to feed and watch after the cattle. One (F) of the FGD 1 participants has mentioned that:

*“…we allocate days and time for the members to take care of the cattle once in 19 days since they are 19 members. A member on his/her duty day watches the cattle, clean their abode, and feeds them. We must abide by the rules and regulations of our group; and if there is a failure in this regard, they would face a punishment of 30 ETB the very first time, and 50 ETB for the second time. But it's shown that the members don't genuinely adhere to their group's norms and abide by the rules and regulations. The accountant and monitory body of the group are also obligated to save their financial resource to the local bank on time and withdraw at time need arise. The monitoring committee of our group also attempts to follow our activities, statuses of our cattle, and the group’s finance…”* (FGD 1, personal communication, October 27, 2020).

Likewise, as to the discussants of FGD 4, the members care for the oxen on a routine basis. Some group members are, however, reported as reluctant. The quarrel happened and they informed the woreda level stakeholders. Basing this, they expelled eight individuals from the group membership by giving them their savings. Members were then minimized to twelve. Soon the members were divided into two having six members each. And later on, the twelve members were divided into three groups, each group having three individuals. The same happened to their oxen; they were shared among the three groups (FGD 4, personal communication, October 30, 2020).

### Perception of the members towards working in a group

The researchers investigated the CIGs members’ perception of working in a group and/or their membership in CIGs. *“...being in group entity it-self has its positive sides…"* said one (M) of the discussants of FGD 2. Others added that working in a group gave them numerous benefits which each of them could not accomplish on their own and at ease. More importantly, it provides a sustainable means of livelihood and employment for the members (FGD 2, personal communication, October 28, 2020). Contrary to this opinion, discussants of the FGD 4 reported that it is good that members are supposed to have the same interest and reside in the same area which is commendable for effectiveness; however, as members’ number increases, their effectiveness diminishes as sundry unmanageable ideas emerge. As to one (M) of the discussants from the same group:

*“…had our group build from only 3-5, we could have a relatively comparable idea and become more effective. But as our number increased, we would have diverse interests, and the probability of having conflicting ideas would be wide…"* (FGD 4, personal communication, October 30, 2020).

## The support from stakeholders

The word 'stakeholder' defines individuals, groups, organizations, or political entities with a specific stake in the outcome of a decision, or impacted by a policy, project, or proposition (Economic and Social Commission for Asia and the Pacific (ESCAP)), 2018). For the question regarding whom they consider as stakeholders, the district’s AGP coordinator replied that:

*“…in the context of our program in general and activities related to CIG in particular, stakeholders are all the individuals or groups interested in the progress and results of our overall activities. They could be from the government’s line offices, the group of young people we are working with, the wider community, the donor [even though we don't have close contact with them]. In light of this, the support comes from all woreda level governmental stakeholders like the offices of livestock and fishery development, youth and women affairs, and cooperative development. All have a stake in the operation of CIG. Development Agents of each kebele are also working in collaboration with members of the CIGs as they give routine assistances, although the support rendered is not enough and there is yet to be done in satisfying needs…"* (T. Bikila, personal communication, October 11, 2020)*.*

Seyoum further explained that the woreda’s cooperative development office takes part as one of the stakeholders in AGP II. To him, the office particularly wants to encourage the CIG members to enhance their livelihood by helping them to save, improve their income, and encourage them to join the local cooperatives including the saving and credit cooperatives and other multi-purpose cooperatives. However, they encounter various challenges in the implementation in its fullest sense (M. Seyoum, personal communication, October 14, 2020). Besides Zerihun mentioned Oromia Saving and Credit Institution as another stakeholder in which it works on the business plan and members saving requirement, which is about 25%, to get startup capital. As the CIGs engage in livestock-related activities, the role of their office as a stakeholder is reported as paramount. For instance, the CIG which works on oxen fattening needs to get technology-related support as they are required to avail their product within three months. Thus, their office checks the livestock’s health status and their potential to get fattened. It is after these procedures that the livestock were bought and then the CIGs are followed up for their best possible performance (H. Zerihun, personal communication, October 15, 2020). The main activity of the women and youth affairs office, as a stakeholder, is creating awareness about the CIGs and their benefits so that youths and women are well equipped with the benefit they would gain from working in a group. It is this office that helps for the smooth communication among members of the CIGs like when there is a problem among CIG members they solve it in collaboration with other stakeholders (T. Fitsum, personal communication, October 13, 2020).

Concerning the support from the AGP II coordination office, Bikila stated that it's their office that coordinates the work of the mentioned stakeholders. As to him, they work on both women and youths without discerning against their gender; they encourage youths to engage in CIG, and they encourage women and men also. They do not exclusively support either male-headed or female-headed households. They do not have a gender-disaggregated support package (T. Bikila, personal communication, October 11, 2020). As part of a stake in the operation of CIGs, the relationship between CIGs and other comparable groups is also explained. For instance, one (M) of the discussants of the FGD 2 said:

*“…although another group consisting of women exists in the kebele, we are not as such close to them. What we do is we merely share information among ourselves…”* (FGD 2, personal communication, October 28, 2020).

## The situation of market linkage

Since the AGP II aims to change the buying and selling culture from the ‘occasional’ and ‘opportunistic’ sales transactions to a more consistent sales approach that builds relationships between groups of organized farmers selling to known trading partners, the researcher intended to examine the actual market linkage situation. In this regard, the discussants of FGD 1 stated that they don’t have exclusive market linkage as per the program’s aim, rather they sell their product-butter, on their own. They sell their product at the local market through the usual sale channel directly to end customers (FGD 1, personal communication, October 27, 2020). Discussants of FGD 2 and FGD 4 reported that they were told by the local government that some kind of market linkage would be created for them. However, they carped that no market linkage has been facilitated thus far, and they merely depend on the local market (FGD 2, personal communication, October 28, 2020; FGD 4, personal communication, October 30, 2020). This happened, according to Truneh:

*"…the government entities, AGP II coordination office, and other concerned stakeholders are all reluctant to do activities related to the market linkage, regardless of the group's (I mean at least the group in our kebele) attempts of producing more sheep as the years pass…"* (A. Truneh, personal communication, October 28, 2020).

Likewise, discussants of the FGD 3 stated that although they were told during the training that they would get more access to the market, the promise was not fulfilled. This in turn discouraged them, stated discontentedly (FGD 3, personal communication, October 29, 2020).

The market linkage issue has risen for the concerned stakeholders at offices to triangulate what the CIG members have said. Accordingly, Seyoum admitted:

*“…yes, the market linkage is needed but the CIGs are not yet developed enough to that level. Market linkage is very important when CIGs are strengthened. The dairy-based groups could be beneficial in this regard, but they are limited in number which made the supply of their product negligible. So, there is no motive to link them with the concerned business organizations found in other areas. But in the future, we would likely arrange market linkage…"* (Seyoum, personal communication, October 14, 2020).

## The performances of the CIGs

The study examined the performance or effectiveness of the CIGs in the study area. In light of this, Bikila asserted that only a few of the CIGs are still operating and getting benefits. He mentioned a group of dairy products as an exemplary, among the ones which get benefit this time (T. Bikila, personal communication, October 11, 2020). Seyoum also stated for the lopsided performances of the CIGs. As to him, there is a relatively successful CIG that sold their products four or more times, like the CIG located in Jemjem-Mela kebele. To him, only a few of them sold their products once or twice and benefited from it; whereas other CIGs can be considered as low performing, and some were dissolved because of various reasons. He rated the CIGs' performance as generally at a medium level or at the average performance (Seyoum, personal communication, October 14, 2020). Zerihun, in the same tone, asserted that:

*“…there is a difference in the performances among the CIGs. The CIG at Jemjem-Mela is exemplary in its performance. CIGs in Dhaye-Tuti and Lencho-Borso kebeles are considered better-performing ones. Conversely, some kebeles have low-performing CIGs due to the dearth of follow-up. For instance, CIGs in Shenkora-Shesheng and Aware-Golje kebeles which participated in goat production and fattening are low performing CIGs due to various reasons; and CIG at Wale-Chilalo kebele is the failed CIG one. Medium performing CIGs are located at Jamo-Berdada, Olantu-Largi, and Dambaza-Wole kebeles. In areas where stakeholders and members work well, the CIG are rated as better performing ones, but the other way-round happens when the stakeholders and the member could not discharge their responsibilities…”* (H. Zerihun, personal communication, October 15, 2020).

Fitsum also said that the applications and implementation of the CIGs in their woreda could be considered as a medium; and despite the failures that occur most often, members are benefiting in one way or another. As to him, once the group members have joined the CIGs, they can engage in other livelihood activities, it doesn’t hamper their participation in other IGAs. But they couldn’t able to go per their execution plan because of various challenges they have been encountering (T. Fitsum, personal communication, October 13, 2020). Fasil, on the other hand, noted that CIG located at their kebele is productive and notable in their milk production. In this way, it augments the income of its members (Fasil, personal communication, October 19, 2020). Another DA, Truneh, remarked on the performance of oxen fattening CIG at his locality as follows:

*“…the group’s productivity was going good and remained hopeful. However, as time goes, their productivity declined and the group was dissolved. Yet, the members benefited from the CIG as their income and livelihood were improved because they shared the cattle population up on dissolution…”* (Truneh, personal communication, October 28, 2020).

Study participants explained the performance of their group by mentioning the expenditure and income they have garnered. For instance, discussants of FGD 1 stated that the big expenditure of the group is buying fodder for the cattle. This expenditure is away great and increasing through time; as an example, if the grass was 2000 ETB it is 5000 ETB now. The by-product of *teff* which was 200 ETB two years ago costs as high as 500 ETB now. With this, one (M) of the participants from the same group described:

*“…these expenditures do not commensurate with the income we garner from butter sale thus far because of the problems mentioned by my friends, particularly transportation, electricity, and fodder related. The income from the butter can be 500 ETB per month on average, but the expenditure would stretch up to 4000 ETB per month. Yet we somehow benefited from the group because we were able to buy 7 more cows in the last three years. But the income we are supposed to have from our group's job is highly reduced and the benefit we earn is lesser of our expectation…”* (FGD 1, October 27, 2020).

Discussants of the FGD 2 have also explained that the group membership enabled them to work collaboratively for sustaining their livelihoods. As to them, it was beyond a mere journey of revenue-making for their daily needs. It is helped them to get employed which most of them used to lack. At the time they sold the sheep for 62,000 ETB in the first year of their establishment, each member shared an income of 6000 ETB. They had to share the income at that time because they need money to buy agricultural inputs such as fertilizers and improved seeds. Even though they have been selling sheep from then on, 62,000 ETB was the highest income they have gained so far. Initially, they had 55 sheep, but they have got about 80 at the time of this study (FGD 2, personal communication, October 28, 2020).

The poultry production group has sold about 622 hens during their operation time and earned 39500 ETB, distributed 1975 ETB for each member. However, the income they got did not commensurate with the efforts they exert. It takes three months for small hens to be grown fully, incurring more expense than the income they generate from the production. Nonetheless, they have affirmed that regardless of the group’s malfunctioning as it is supposed to be; they reaped benefits in terms of motivation and experience. They also said they were able to create social capital and the interaction and communication they have in the group served them as a source of information for the comparable activities they are engaged in (FGD 3, personal communication, October 29, 2020).

The last group, the oxen fattening group, wanted to participate in the buying processes to buy more oxen with the money they pooled together and received from the AGP, but the woreda stakeholders didn’t allow them to participate. The woreda people bought them 9 oxen. The group later on divided into two acquiring 4 and 5 oxen. Some of the oxen got fatten and others did not. This created a conflict among the members. Yet, they have sold their products [the oxen] three times. During their first-round sale, each member of the group earned a profit of 700-1400 ETB; and 1200 ETB in the second round. Other times, they got a profit of 1100-1600 ETB. They bought 7 improved oxen breeds and sold them back within 2 months. The implementing stakeholders didn’t recommend the division of profit among the group members. But the group beneficiaries justified it as ‘we divided the group’s profits due to dearth of money they encountered including lack of inputs for fattening’. Lastly, they dissolved the group and share the oxen among themselves individually. Yet, some of them reported as they benefited even if their group has dissolved. For instance, the chairman of the group bought an ox for 16,000 ETB and it for 22,000 ETB within 6 months. Most of them, however, shifted to other off-farm and non-farm IGAs (FGD 4, personal communication, October 30, 2020).

### Strengths of the groups

FGD participants mentioned the creation of employment opportunities for themselves and their families as the first strength of the CIG. The other strength was attributed to its effect on social networking helping at times of misfortune and hardship. Being a member of the CIGs does not consume all of their time; it rather provides opportunities to generate income. Thus, an opportunity to participate in IGAs by itself is also mentioned as a strength (FGD 2, personal communication, October 28, 2020). Besides, as to the discussants of the FGD 3 working together and solidarity among the members is what they consider the strength of the group. AGP’s initiation and members’ willingness and ability to fulfill the requirements like saving were also considered the strength (FGD 3, personal communication, October 29, 2020). Participants of the FGD 4 added that CIG helped them secure and diversify their livelihoods as they did not have any source of livelihood before joining the CIG (FGD 4, personal communication, October 30, 2020).

The establishment of CIGs based on members' interest, formulation of the proposal upon their establishment, members' contribution for a startup capital, the presence of relevant stakeholders for the CIG activities implementation, the occurrence of support and monitoring offered [though minimal] are all mentioned as the strengths (M. Seyoum, personal communication, October 14, 2020). Zerihun added:

*“…the CIGs in a given kebele has created job opportunities for their members. For instance, the two CIGs each kebele having 20 members have created job opportunities for 40 people. In light of this, the money given by the AGP as a startup capital could be considered as one strength of this scheme as it enables members to engage in the business they need to have…”* (H. Zerihun, personal communication, October 15, 2020).

With regards to startup capital, Truneh also asserted that:

*“…it is the money collected from the CIG members and the money given by the AGP that let them start the business, but it could be said that the money they have given is not sufficient to strengthen and continue their business...”* (A. Truneh, personal communication, October 28, 2020).

An opportunity created to work together paving a road for additional income which otherwise cannot be accomplished individually, and access to the market which in turn help them access some agricultural inputs like herbicides and pesticide are also reported as the strengths of the CIG (K. Fasil, personal communication, October 19, 2020). Bikila also stated that the CIG helped farmers to come together and build a social capital which in turn enhanced their capability and togetherness (T. Bikila, personal communication, October 11, 2020).

### Weaknesses of the groups

With regards to the weaknesses of the CIG, discussants of the FGD 2 reported that they cannot endure protecting the health of the sheep due to the conventional feeding practice they have been practicing. They weren't able to build a well-arranged barn for the protection of their sheep. Such a failure negatively affects their effectiveness in terms of income and related benefits. Besides the treatment of the sheep is not scientific yet, and they did not monitor them carefully partially because of the number of sheep they own (FGD 2, personal communication, October 28, 2020). The prevailing pessimism among members and the inability to give a recovery time among the stakeholders were also reported as weaknesses (FGD 2, personal communication, October 28, 2020). Also, participants of the FGD 4 stated that members lacked all-rounded knowledge and proactive means of securing their business. As to them, members should have worked to build a reserve for the oxen rather than searching for excuses for their failure and dissolution. They also lacked cooperation and team orientation. Although the business they are in is perfect and timely, the support from the government side and other stakeholders was reported as discouraging. They did not get training on a routine basis. They assume there would have been more benefits had they got appropriate training (FGD 4, personal communication, October 30, 2020). Zerihun, on the other hand, mentioned the lack of participation of DAs and the CIGs members in buying the livestock, lack of sense of ownership and absence of coordination among the stakeholders, and lack of monitoring and evaluation as weaknesses. As to him, stakeholders of the CIGs meet up to do a follow-up merely two or three times per annum. In light of this, the CIG at Jemjem-Mela is reported as the most visited and monitored than others. Yet, the frequent visits conducted were not in a format of supportive supervision, rather it was an experience sharing for other CIGs. For the failure related to monitoring and supervision, the respondent has rationalized that their office has a lot of tasks that couldn’t allow them to engage in supportive supervision activities (H. Zerihun, personal communication, October 15, 2020).

Likewise, Bikila stated that the dissolution of the CIGs has resulted from a loss of interest in working together. As to him, there is a lack of regulation and strict laws to abide the members and it was only the money they have contributed that was used to seal them. Lack of monitoring and evaluation was also mentioned as another weakness of the CIGs. He further explained:

*“…even though DAs are in charge of facilitating the main activities of the CIGs, the processes of buying the cattle and oxen exclude them. It was rather conducted by me, and officers from our woreda’s cooperative development office and finance office. This in turn makes the DAs feel and consider the AGP activities as an extra-work not giving needed attention…”* (T. Bikila, personal communication, October 11, 2020).

Seyoum mentioned the absence of a guideline as to the weakness of the CIG. As to him, they don't have a guideline that did hold them together by indicating how to operate and what to do at times of dissolution. The CIG has failed to bring farmers who have common interests and objectives into one entity. Members are not equally responsible for the group’s activities. This in turn remains a fertile ground for members' dropouts. Inability to solve conflicts and failure to sustain the CIGs is also stated as a weakness. Additionally, he explained the CIG’s weakness as follows:

*“…the woreda's cooperative development office helps to save, enhance their income level and encourage them to join the local cooperatives including saving and credit cooperatives and multi-purpose cooperatives. However, many problems are encountered for its implementation in its fullest sense. This is mainly because members lack money. Being members of these cooperatives, the majority of the members cannot afford the required monthly payment. Only relatively better-off individuals who earn additional income from other businesses joined the cooperatives. In addition to this, they don't have a platform to solve conflicts and/or differences usually happens to them…”* (Seyoum, personal communication, October 14, 2020).

Consistent with Seyoum’s idea, Fitsum stated that the CIGs do not have a required mechanism of control. Members have failed to consistently save the required amount of money. To him, the presence of conflict among members, absenteeism from groups' activities, and negligence altogether ended most groups in dissolution. Furthermore, he asserted:

*“…lack of market linkage and lack of proper application of plans at the local level could also be mentioned as the weaknesses of the CIGs operating in our district. The absence of uniform knowledge among the stakeholders and the CIG members could also be mentioned as the significant weakness…”* (T. Fitsum, personal communication, October 13, 2020).

Fasil and Truneh have shared Seyoum's idea by emphasizing conflicts and quarrels as the main weaknesses of CIGs which remained challenges to their stability (K. Fasil, personal communication, October 19, 2020; A. Truneh, personal communication, October 28, 2020).

### Opportunities both for the members and local people

In addition to the benefits the CIGs render for the immediate beneficiaries (i.e. their members), the respondents stated the scheme's advantages and/or opportunities for the local people where they operate. Zerihun, for instance, stated that the CIG has remained proof for the area has untapped potential for cattle breeding and fattening. It also enables the youth to build on their potential and get employment opportunities. It also remains the confirmation that anyone who works can get benefits and means of livelihood. Basing these, he rated the implementation of CIG in their area at an average level (Zerihun, personal communication, October 15, 2020). Fasil has also reported:

*“…even though the CIG has somehow improved our farmers' livelihood by helping them tap their potential, it is hard to count a groundbreaking new development because of the CIG interventions…”* (Fasil, personal communication, October 19, 2020).

Congruent with Fasil’s opinion, participants of the FGD 2 stated that their job has more or less satisfied local people’s demand for sheep by enhancing its availability. Their activity has also served as a lesson for local people that sheep fattening and producing is a relevant and feasible business in the area. It is because of them that people in their locality nowadays form groups and do the same (FGD 2, October 28, 2020). Discussants of the FGD 3 have also stated that the nearby communities have acquired experience related to poultry production after the visit they had to their group's business. Most of these visitors have then established poultry production businesses in their yards (FGD 3, October 29, 2020). The same is reported by participants of the FGD 4 stated that other members of the community have learned a lot from them via the experience sharing platform facilitated by Das at their kebele. In response to this contribution, some villagers offered them grazing land as encouragement (FGD 4, personal communication, October 30, 2020).

### Threats the CIGs have encountered

With regards to the problems or threats the CIG has encountered, Bikila underscored the support to be given from the CIG’s lead implementers like the livestock office, youth and women affairs office, cooperative development office and DAs are not adequate. For instance, some DAs have assumed the AGP as NGO, and consider AGP introduced CIG activities as an extra-work falling outside the scope of their service. Lack of due evaluation was also mentioned as another threat (Bikila, personal communication, October 11, 2020). DAs reluctance to fully engage in the activities of the CIGs was also reported as the main threat by Fitsum (Fitsum, personal communication, October 13, 2020). Seyoum mentioned the non-existence of stakeholders’ participation as the threat, and explained their loose engagement as follows:

*“…there is loose coordination among the CIG stakeholders. This could perhaps be because the kebeles are geographically far from each other and some of them are bounded by large geological features prohibiting the frequent access of stakeholders. The absence of required dedication from the DAs has also contributed a lot. Most of the DAs don’t discharge their responsibilities on the ground but usually comes with monthly basis reports and annual report indicating their progress…”* (Seyoum, personal communication, October 14, 2020).

Lack of uniform knowledge among the stakeholders and the CIG members is also reported as a threat. The experts from lead stakeholders have not been providing the support they required to give to the desired level. Even most of the stakeholders are not in touch with the CIGs and don't know their progress (Fitsum, personal communication, October 13, 2020). Another threat is the lack of homogeneity among members of the CIGs. As an indicator, some CGIs embrace elementary-level farmers and graduate youths together as their members. Provided that they don’t have group norms and regulations, such a difference has created a conflict of interest at times these graduates secure jobs resulting in their dissolution (Bikila, personal communication, October 11, 2020). Lack of adequate startup capital is also mentioned as a problem. The budget allotted for the CIG is low. For instance, the program allocated a maximum of 100 thousand for about 20 individuals, by which they can only able to buy three oxen. This has also created a problem at the time they share profit (if there is any). Lack of budget has also hindered the stakeholders from conducting appropriate monitoring and evaluation (T. Bikila, personal communication, October 11, 2020; H. Zerihun, personal communication, October 15, 2020).

Participants of the FGDs have also reported lots of problems they have encountered in CIG businesses. For instance, FGD 1 mentioned the non-existence of transportation services in their kebele to be able to transport the milk to the nearby urban area. This in turn forced them to merely depend on butter production and its sale as the major business activity. One (F) of the participants affirmed:

*“…we don’t have power in the kebele to use a refrigerator for the milk. We also have been facing problems with the inputs for the cattle (cows and calves). We use the local grass as the cost of an improved fodder skyrocketing. Besides the money we received from AGP was quite small to unlock our potential in milk production. We had to buy low-quality cows. Had the budget not been low, we could have bought improved cow breeds…”* (FGD 1, October 27, 2020).

Participants of the FGD 2 have also reported that they had lost numbers of sheep at the very commencement of their business attributed to the then health problem affecting sheep. Their area for sheep farms was also not adequate. Although the government promised to provide them with a space, they were not given one and neither did materials for the construction of the sheep abode (FGD 2, October 28, 2020). Workspace problem was also raised by FGD 3 and 4. For instance, AGP has provided members of the FGD 3 with the poultry during the rainy season, unfortunately, the poultry requires warm places. Their poultry production was thus hampered by the then cold environment. The AGP didn’t provide them with materials to rescue their poultry though they had requested the same (FGD 3, October 29, 2020). Participants of the FGD 4 mentioned the absence of inappropriate training, inadequate services from the stakeholders, and lack of fattening inputs in addition to a lack of spaces where they can keep their oxen and do fattening. These all made them not work in a group (FGD 4, October 30, 2020).

## The way forward to get benefit from the CIGs

With regards to what needs to be done to enhance the performances of the CIGs, Bikila recommended:

*“… guidelines should be developed by the respective group members and they should feel a sense of group belongingness. And guidelines should be timely and needs to be updated and strictly enforced. When, why, and on what precondition shall members leave should be clearly stated. Whether and if the profit should be shared or not or its timings of share should also be taken into account…”* (Bikila, October 11, 2020).

Coordinators at the regional and zonal level and technical advisors of the program at the federal level have emphasized the importance of the roles to be played by the lead stakeholders (like the livestock agency, cooperative development office, youth and women affairs office, and agricultural office). As to them, roles to be played and responsibilities to be discharged by these offices should be clearly stated and informed. The mutual accountability they have in the implementation of the CIG should also be ensured (N. Ayelech, personal communication, October 17, 2020; M. Seid, personal communication, October 2, 2020).

It was also suggested that engagements of the woreda level CIG stakeholders, DAs, and kebele level administrators in the monitoring and supportive supervision activities should be strengthened; they have to participate in CIG activities starting from participation in livestock procurement (T. Bikila, personal communication, October 11, 2020; T. Fitsum, personal communication, October 13, 2020; M. Seyoum, personal communication, October 14, 2020; H. Zerihun, personal communication, October 15, 2020).

To enhance the productivity of CIGs, continuous support on the types of fodder the CIGs shall use was also suggested (T. Fitsum, personal communication, October 13, 2020). He further recommended:

*“…CIG should have a binding code of conduct, and rules and regulations shall be set as a mandatory and pre-requisite to their establishment and operation. Adequate budget allocation should be considered for DAs while conducting the CIGs activities in general and its monitoring and control activities in particular. Additionally, the issue of market linkage for the CIGs should be considered…”* (Fitsum, personal communication, October 13, 2020)*.*

Discussants of the FGDs 2 and 3 have suggested three schemes for the effective performance of CIGs. One is the workspaces should be first arranged. Second, their activities should be diversified. Third, a union shall be formed among the CIGs; and the last one, the market linage shall be established (FGD 2, October 28, 2020). And (FGD 3, October 29, 2020). Likewise, Truneh stated:

*“… the space where the livestock is to be kept, and their feeding system should be prepared before forming the CIG group..”* (Truneh, personal communication, October 28, 2020).

### Members' perspectives on the futurity of their groups

It is found that none of the studied CIGs want to dissolve their groups. Instead, it happened that those whose groups are yet functional are highly in need of perpetuation of their groups; and those whose groups dissolve consider it unfortunate and wanted to get it back. But both the functioning and the dissolved groups want the government and other stakeholders to re-consider all the bottlenecks they have stated above. Participants of the FGD 2, for instance, asserted that whatever would have happened to them, they are not going to dissolve their group. As to them, their group will exist regardless of the capital shortage or the risk they might face. But they underscored that they need assistance from the government and lead stakeholders of the AGP II for the better functioning of their group and to meet their very intent of establishment (FGD 2, October 28, 2020). FGD 3 participants, on the other hand, stated that most of their members benefitted at the time their CIG was dissolved since they shared the hens and their products among themselves and owned them privately. This made some members develop a negative attitude toward their membership as they face more risks and debts than benefits. After a heated argument the group concluded that since all their failures have merely emanated from the risky nature of their business (i.e. poultry production), and unlike other CIGs in their area quarrels and/or disagreements that obstruct their functioning have been non-existent, they want to continue with their group; but with different livestock related business other than a poultry production (FGD 3, October 29, 2020).

Likewise, two (M and M) of the discussants of the FGD 4 have stated that it would be better if they continue with their group but the number of members should be minimized and each of them has to get to know one another and work closely. The two discussants added that individuals who establish a particular group should be those who live in the same residential area and are well-known to each other. Another (M) respondent from the same FGD has said that:

*“…up on our establishment as a group, members shall be from the same kebele and each of the members’ effectiveness and background should be assessed. The government shall also monitor and follow our group per week or a month. The stakeholders including the government did not clearly and carefully control our group’s tasks. Follow-up should be followed by merits for accomplishments and punishments for wrongdoers. The government should not be reluctant in that regard…”* (FGD 4, personal communication, October 30, 2020).
