# Supplementary material for: Effects of common interest groups on rural women and youth livelihood: A qualitative study from Central Ethiopia
Source: PLoS One. 2023 Oct 20;18(10):e0283532. doi: 10.1371/journal.pone.0283532 (PMC10588890; doi:10.1371/journal.pone.0283532)
Supplement: S7 File — (PDF) [file pone.0283532.s017.pdf]

Addis Ababa University
አዲስ አበባ ዩኒቨርሲቲ
SEEK WISDOM, ELEVATE YOUR INTELLECT AND SERVE HUMANITY !

COLLEGE OF DEVELOPMENT STUDIES (CoDS)

Institutional Review Board (IRB)

No: 001/02/2023

Ph.D. Proposal Ethical Clearance Certificate

1. **Student's name:** Solomon Zewudu Leul **Gender:** Male **Birth Date:** April 19/1988
   Id.No: GSR/7653/10 **e-mail:** slmnzwd@gmail.com
2. **Home Center/Dep't: CoDS:** Center for Rural Development **Stream:** Rural Development
3. **PhD Dissertation Supervisors:**

   Alemu Azezew (PhD) Email: alemu.azmeraw@aau.edu.et
   Solomon Tsehay (PhD) Email: Tsehaysol2015@gmail.com
   Alemseged Gerezgiher (PhD) Email: abushalex2@gmail.com

4. **Title of the Proposal:** ATTAINING SECURE RURAL LIVELIHOOD: PRODUCTIVITY, INCOME, COMMERCIALIZATION, AND WELFARE IMPACTS OF THE AGRICULTURAL GROWTH PROGRAM II INTERVENTIONS ON SMALLHOLDER FARMERS OF NORTHWEST ETHIOPIA.
   a. **Proposal No:** N.A. **Date accepted:** February 17, 2023
   b. **Amendment No (if any):** N.A. **Date:** N.A.
5. **A clear statement of the decision:** This proposal was reviewed and approved by the Academic Commission of Center for Rural Development Studies some time before the approval of Standard operation procedure (SoP) of the College. After it is learnt from the statement of the applicant that, having an ethical clearance certificate is required for publication process, it is believed to review the content of the proposal, its associated research tools and informed consent of the respondents retrospectively. As a result the proposal found to be qualified for the ethical clearance.
6. **Decision:** This proposal fulfills the standard requirements described in IRB-CoDS Standard operating Procedure (SoP) and ethical clearance is hereby awarded.
7. **This certificate is issued upon the consent of:** IRB-CoDS.

IRB-CoDS

Name: Teshome Tafesse (Ph.D)
Designation: Chairperson of CoDS/IRB
E-mail: cods.irb@aau.edu.et

Approved

Signature:...............................
Date: February 27, 2023

Addis Ababa University
College of Development Studies Institutional Review Board (IRB)

This certificate is valid only sealed and signed
