# Supplementary material for: Effects of common interest groups on rural women and youth livelihood: A qualitative study from Central Ethiopia
Source: PLoS One. 2023 Oct 20;18(10):e0283532. doi: 10.1371/journal.pone.0283532 (PMC10588890; doi:10.1371/journal.pone.0283532)
Supplement: S8 File — (DOCX) [file pone.0283532.s018.docx]

**Checklist for the Focus Group Discussions**

1. Would you please tell me when do your Common Interest Groups (CIGs) established?
2. What are the activities you are conducting (or engaged in) via CIGs?
3. Why you joined your CIGs?
4. Do you think CIGs are helpful to improve farmers’ income and productivity? [Yes or no…explain how]
5. Did you managed generate additional income from being member of CIGs [Yes or no…explain how]
6. What is your perception on the performance CIGs in your locality in which you are a member for the last three (?) years?

Probe: Highly productive

Productive

Somewhat productive

Not productive with adequate explanations

1. How do you perceive the general performance of CIGs?

Probe: Highly productive

Productive

Not bad (fair)

Poor

Extremely poor with adequate explanations

1. Do you think there is visible difference between women CIGs and other CIGs in performance?
2. If yes, which one is more productive?

Probe: Women CIGs

Youth CIGs

Mixed CIGs [explain how]

1. Why do you think they performed better?

Probe: Commonness in interest among members

Commitment and seriousness of members

Smaller membership

Profitability of the line of business (activities) of the group

1. How much have you contributed for the establishment of the CIG (Birr)?
2. Do you think the individual members’ contribution is fair or expensive? [Explain]
3. Have you received market linkage support for selling your crops or livestock? (Yes or No) [Explain]
4. If yes, from which institution your group was linked with

Probe: Cooperatives

Unions

Private buyers/sellers including factories

Government enterprises

1. If yes to [any institution], what benefits you got at being in such cooperative?

Probe: Easy market access

Better price

Easier input access

Not benefited

1. What do you think are the major strengths of CIG in which you are member and CIGs in general?
2. What do you think are the major weaknesses of CIG in which you are member and CIGs in general?
3. What opportunities do you think that CIGs render for you and your locality’s people?
4. What threats are encountering the operations of CIGs in your locality?
5. What intervention strategies could be designed to enhance the operation of CIGs in your locality? [suggestions for future improvement]

**Question guide for the Key Informant Interviewees**

1. Who are target beneficiaries of the AGP II in general and crop technology utilization in particular? [Target group identification]
2. How these target farmers are reached?
3. Have you encountered any problem (challenges) related to the intended target group identification?
   1. For the whole packages of the program?
   2. For crop technologies utilization and practice uses aspect? (technologies and practices for Teff, Wheat and

Maize crops independently)

1. What is the AGP II based processes of crop technology demonstrations?
   1. In Farmers’ Training Center (FTCs)?
   2. At farmers plot?
      1. How effective they are?
      2. Which one is more effective? Why?
2. What looks smallholder farmers’ perception towards crop technologies introduced by the program?

Probe: Increases production and productivity (yield gains)

Improves food security and income

Helps to reduce pest and disease outbreak

Increase crop diversity

1. What looks the SWOT of the crop technologies?
   1. What do you think are the major strengths of crops [Teff, Wheat and Maize] technologies utilization in your

locality?

- 1. What do you think are the major weaknesses of crops [Teff, Wheat and Maize] technologies utilization in your

locality?

- 1. What opportunities do you think that utilization of AGP II introduced crop technologies render for you and your

locality’s people?

- 1. What threats are encountering the operations of crops [Teff, Wheat and Maize] technologies utilization in your

locality?

1. What intervention strategies could be designed to enhance the implementation of crops [Teff, Wheat and Maize] technologies utilization in your locality? [suggestions for future improvement]
2. What looks smallholder farmers’ perception towards the AGP II’s contribution on their crop productivity?

Probe: It increases production and productivity (yield gains)

It enhances their condition of food security and income

1. What are the specific interventions of the AGP II being implemented to enhance crops [Teff, Wheat and Maize] commercialization?
   1. Are they beneficial (important)? If yes, to what extent, particularly with regards to the mentioned crops?
2. Who are the major actors identified in crops [Teff, Wheat and Maize] commercialization?
   1. How AGP II has worked with these actors?
   2. What benefits it renders for crops [Teff, Wheat and Maize] producer smallholder farmers?
3. What market infrastructures were established by the program to support crops [Teff, Wheat and Maize] commercialization?
   1. In what ways they support the processes of commercialization?
4. What are the major challenges farmers faces in crops [Teff, Wheat and Maize] commercialization?
5. What do you think is the effect of crop technologies utilization on smallholder farmers’ consumption expenditure?
6. What do you think is the differential welfare effect of the program on male and female headed household beneficiaries?
7. What looks like the implementation of CIGs? Is it going in line with the program implementation manual? Or what?
   Probes: its strengths, weaknesses and suggestion for future improvement
8. Would you please tell us new thing that happened because of AGP II interventions/implementation in general?
