# Supplementary material for: Effects of common interest groups on rural women and youth livelihood: A qualitative study from Central Ethiopia
Source: PLoS One. 2023 Oct 20;18(10):e0283532. doi: 10.1371/journal.pone.0283532 (PMC10588890; doi:10.1371/journal.pone.0283532)
Supplement: S11 File — (DOCX) [file pone.0283532.s021.docx]

# Notes

1. Common Interest Group (CIG) is an informal group of 10 – 20 members from the same village who voluntarily agree to cooperate on a profit-oriented agricultural activity that focuses on value chain activities including the production of livestock and crops, post-harvest handling, and storage, processing, and marketing; active natural resource conservation or sustainable use of these natural resources.
2. ‘*Woreda*’ is an administration unit equivalent to a district.
3. ‘*Kebele*’ is equivalent to ‘village’ and refers to the lowest administrative unit in Ethiopia, except the capital, Addis Ababa*.*
4. ‘*Tef*’ is a staple food crop for millions of people in Ethiopia. It is the most important crop by area planted and value of production, and the second-most important crop in generating income.
5. The term stakeholder is frequently used in this study, and it refers to the distinct groups interested in the implementation of CIGs, either because they are directly affected by or involved in the group activities, or because they must make a decision about the group activities or about a similar activity at other locations or times (Gold, 1983; Guba and Lincoln, 1981).
6. The Ethiopian *Birr* (*ETB*) is the national currency of the Federal Democratic Republic of Ethiopia.
