# Supplementary figures and images for: Effects of common interest groups on rural women and youth livelihood: A qualitative study from Central Ethiopia
Source: PLoS One. 2023 Oct 20;18(10):e0283532. doi: 10.1371/journal.pone.0283532 (PMC10588890; doi:10.1371/journal.pone.0283532)

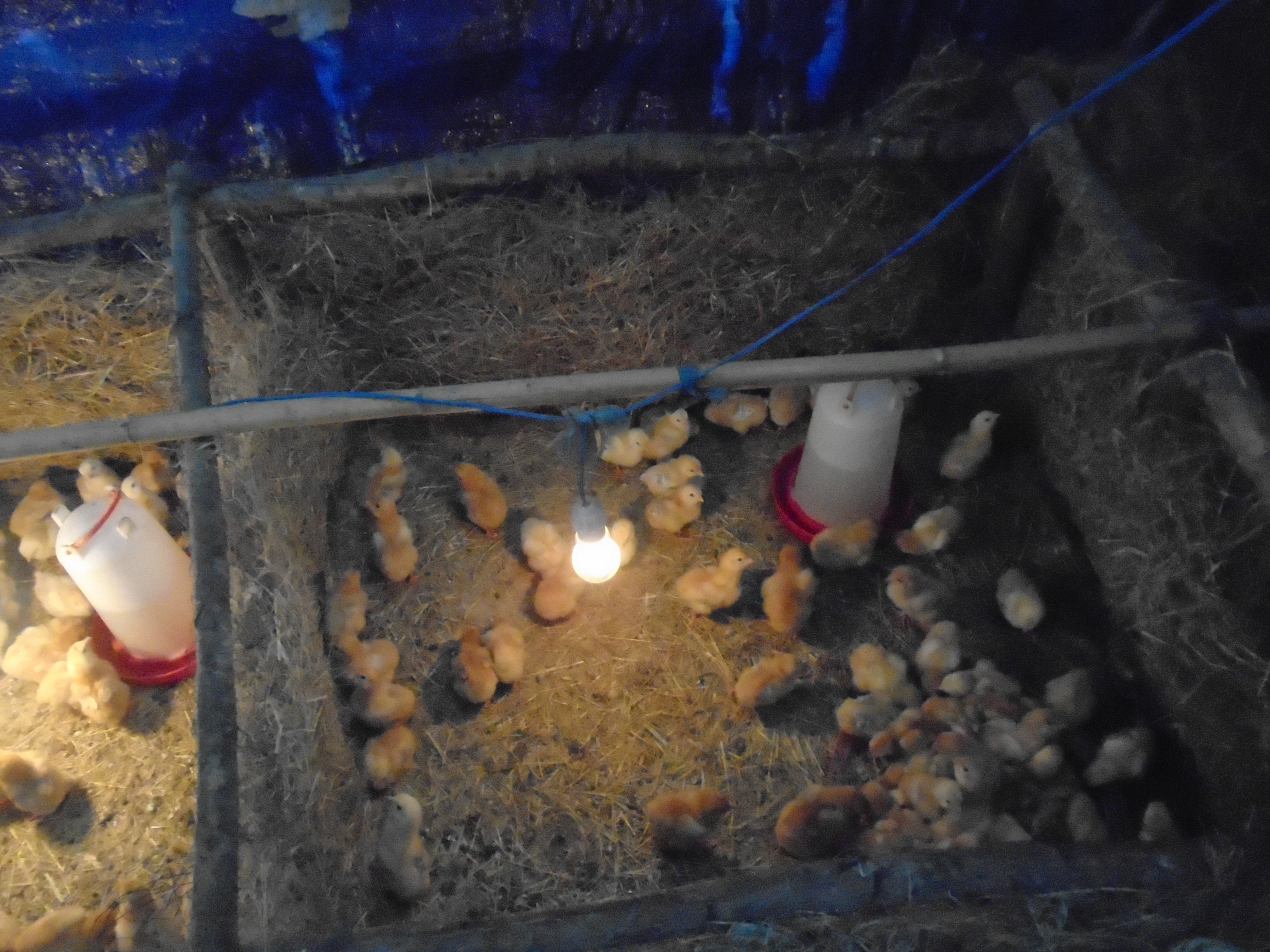

Supplement: S12 File — (JPG) [file pone.0283532.s022.JPG]

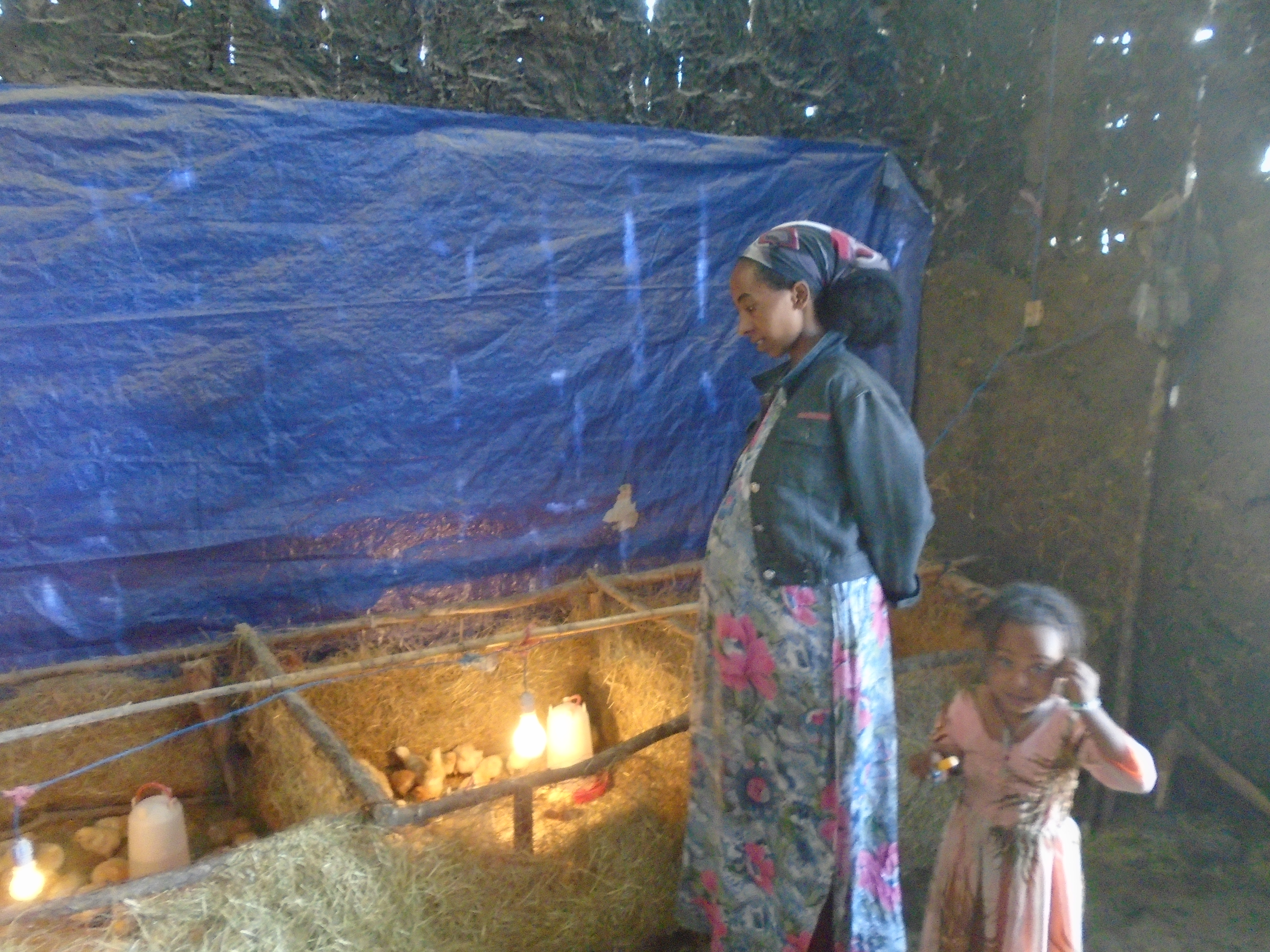

Supplement: S13 File — (JPG) [file pone.0283532.s023.JPG]

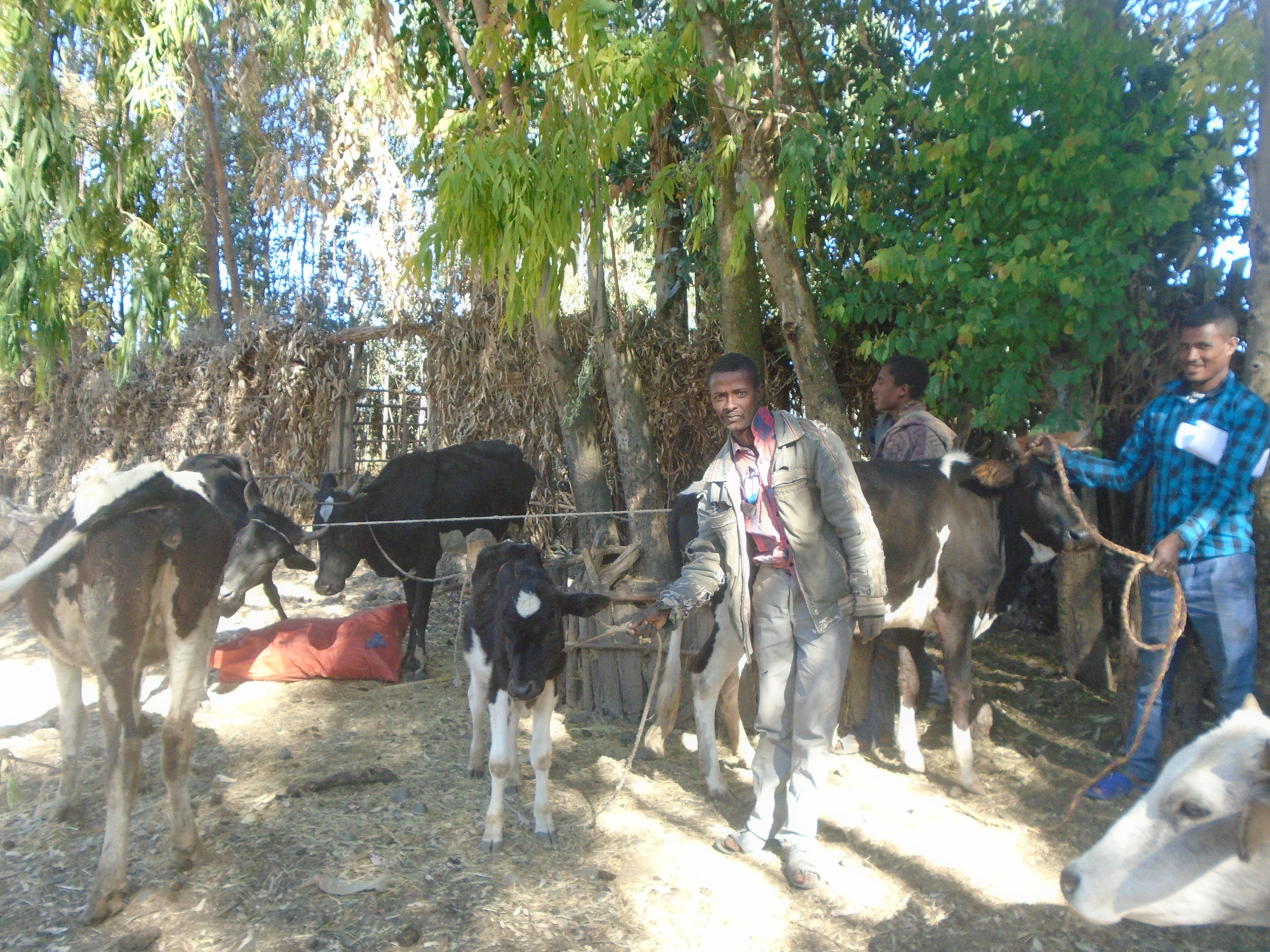

Supplement: S14 File — (JPG) [file pone.0283532.s024.JPG]

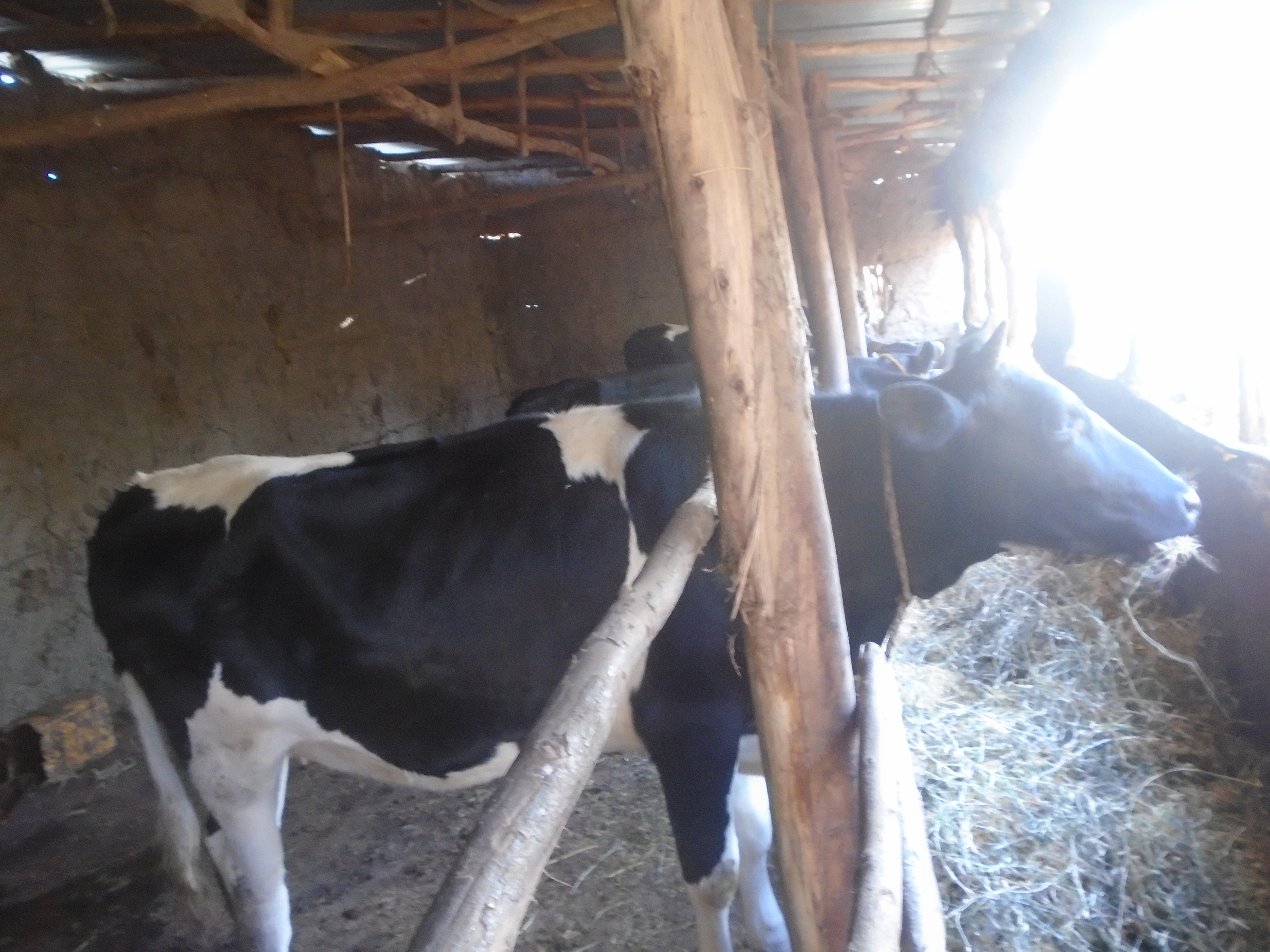

Supplement: S15 File — (JPG) [file pone.0283532.s025.JPG]

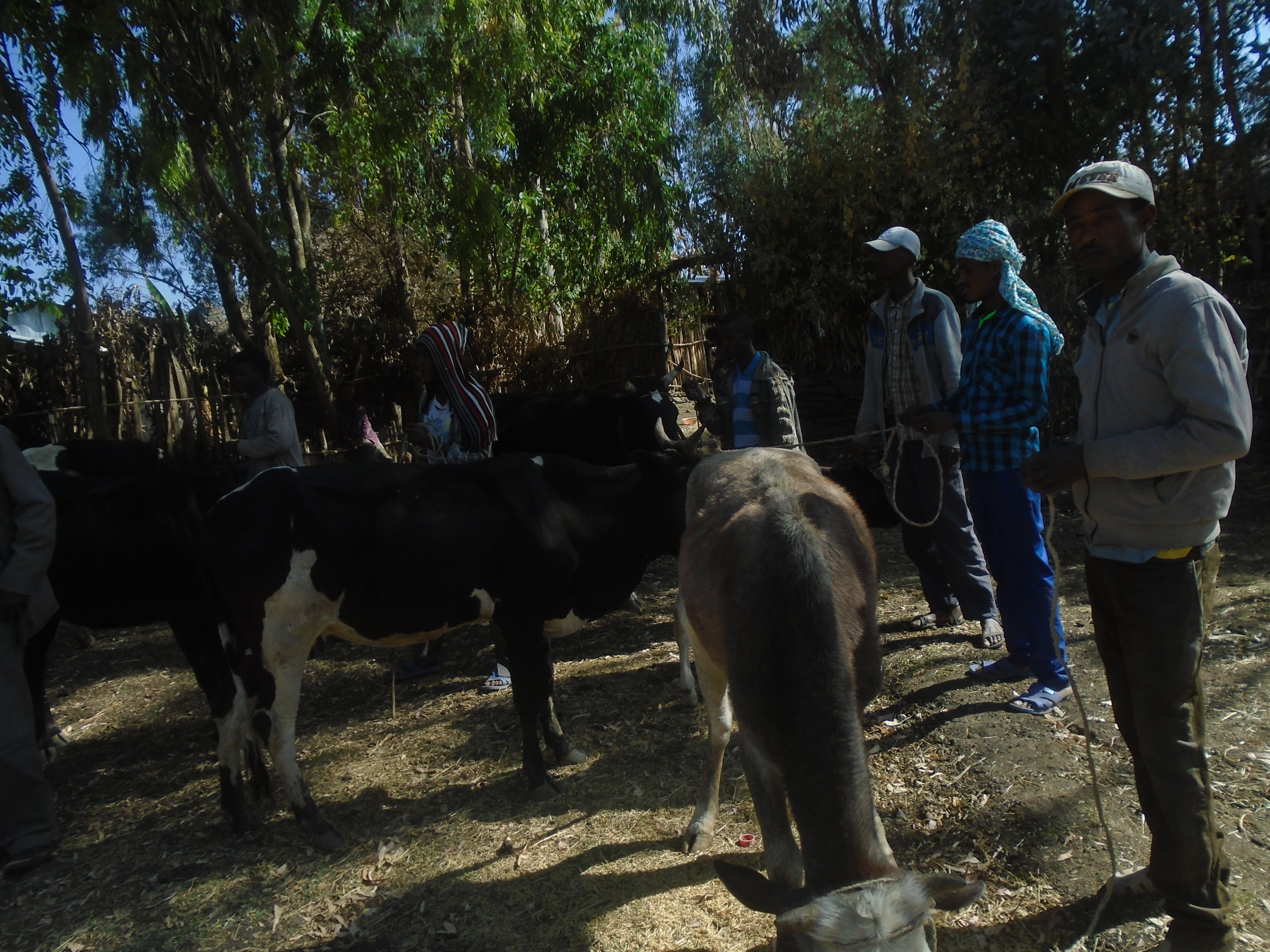

Supplement: S16 File — (JPG) [file pone.0283532.s026.JPG]

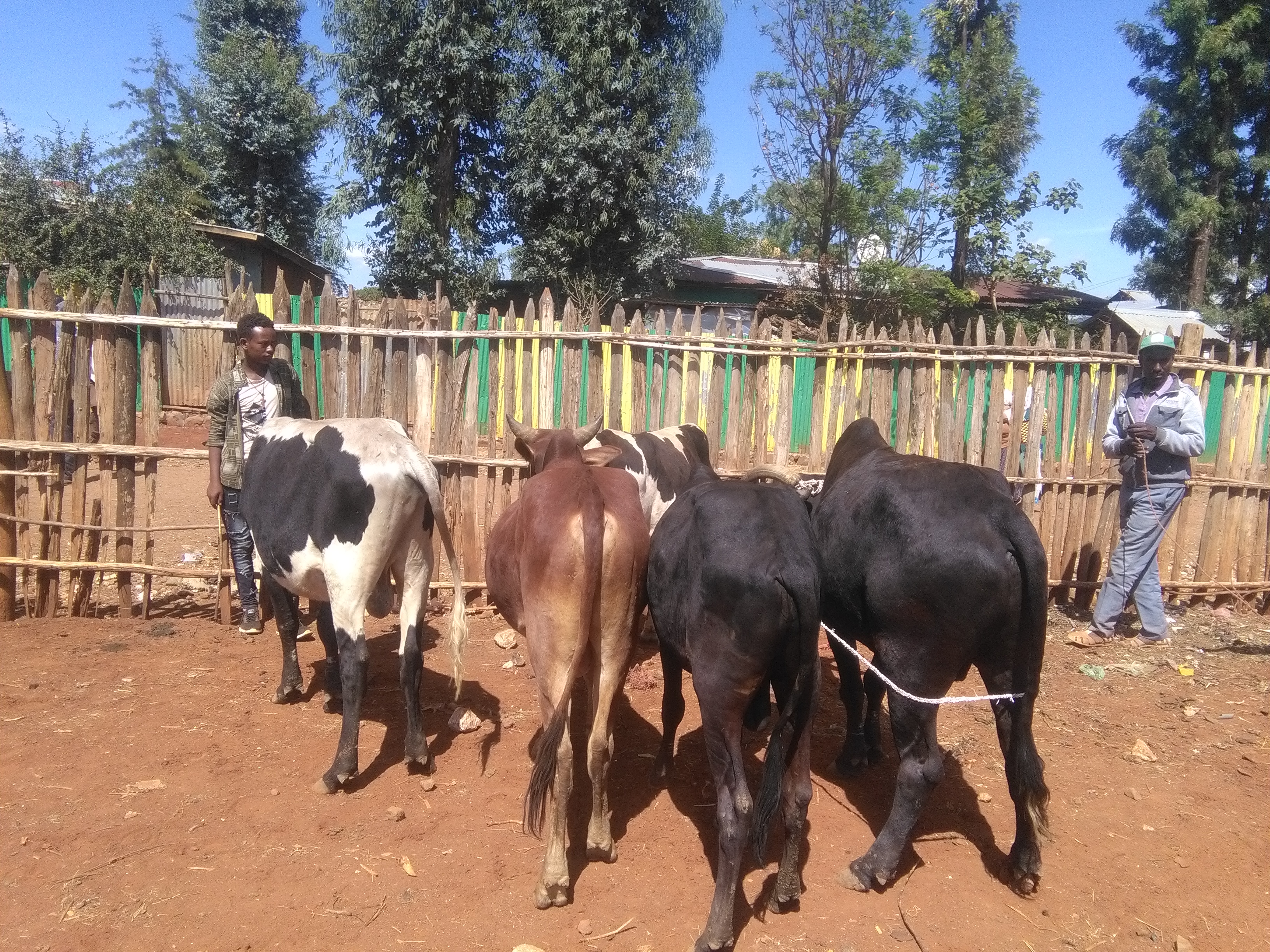

Supplement: S17 File — (JPG) [file pone.0283532.s027.JPG]

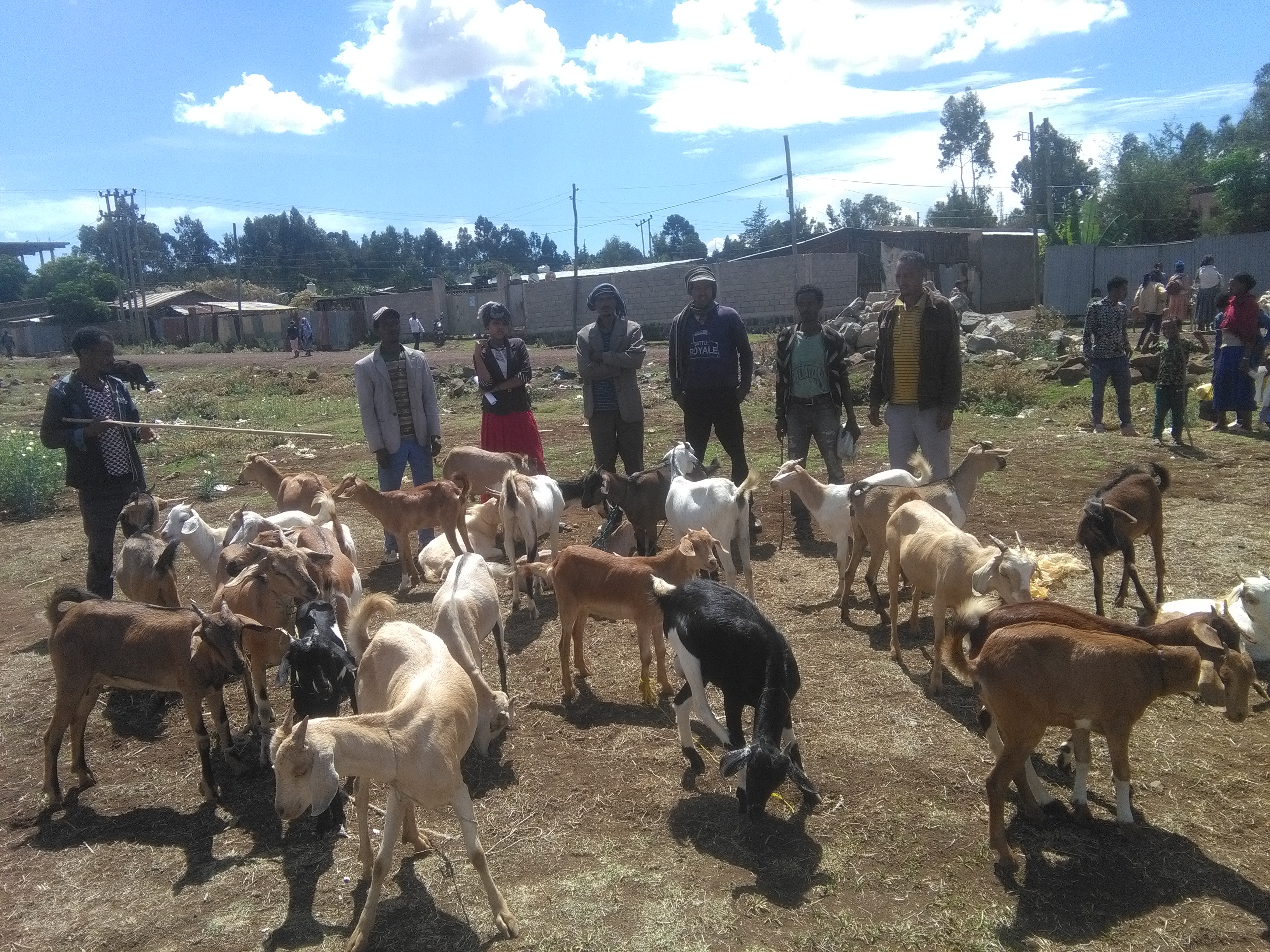

Supplement: S18 File — (JPG) [file pone.0283532.s028.jpg]
