# Supplementary material for: Effects of common interest groups on rural women and youth livelihood: A qualitative study from Central Ethiopia
Source: PLoS One. 2023 Oct 20;18(10):e0283532. doi: 10.1371/journal.pone.0283532 (PMC10588890; doi:10.1371/journal.pone.0283532)
Supplement: S19 File — (DOC) [file pone.0283532.s029.doc]

**With the woreda’s AGP II coordinator (Bikila Tolossa)**

**Introducing the AGP II:**

The AGP focuses on the high productive areas, highly commercialized cereals that could improve the livelihoods of farmers. The farmers should also be capable of adopting new technologies. Particularly, seed multiplication is the focus area of AGP. They work in collaboration with the plant researchers so that the people can adapt to their local situation. Such research sites as Bishoftu, Asella and etc are the major partners. The AGP provide the new and improved seeds for the local farmers by collaborating with the agricultural cooperatives, with the cooperatives organizations. The seeds undergo examination to analyze if it certified or not. If it is certified, the seed can be applied at the country level, if not it should be utilized at the local level among the farmers.

**The problems in reaching the beneficiaries:**

The respondent said, clustering which is one of the main objectives of AGP often fail because the land is not appropriate for it..The farmers sought different types of cereals and there is no similarity among them also. When they select the farmers, they have to face many challenges as the farmers are not often on the same level, while some needs more of fertilizer, some other not. Productivity, and there is no markets for their products and most often the market I of locally one giving less opportunities.

**What does the AGP targets, Individuals or villages?**

The village can be a target, but they focus more in the individual farmers through providing training, seed, etc.75% of village residents are assumed to be beneficiaries of the AGP II.

**The problems in identifying beneficiaries (selection criteria):**

The farmers should be interested in the activities. The capabilities of farmers should also be assessed. Formation of a group is based on their abilities to fit the requirements- ability to sow in rows and buy fertilizers. The agro ecology of the area need to be assessed to know exactly the cereal types in need, they provide a quota for each village. The Dega areas seeds like Teff and wheat are produced. At some desert areas, they focus on producing more of maize.

**Technological Demonstration:**

They conduct the technological demonstration both at the FTC level and farm level. They conduct these to assess the potentials, advantageous and disadvantageous side of production. The budgets are also allocated for this purpose. The FTC level of assessment should show a good result and should succeed after which the farmers adopt and apply at their farm lands. Maize and Teff are the two mostly used seed types in this regard. But lesser for the maize due to the infrastructural limitation to reach areas where these seeds ploughed in large amount which is as far as 34 km away from the woreda.

**Benefits of conducting FTC:**

The practical assessment is more trust-able among the farmers than the theoretical depiction. The practical results also foster the adoption of the technologies. The farmers see the demonstration and carry on their duties based on that.

**Perception of farmers towards the Technologies:**

The farmers are practicing the improved seeds from year to year, their number is rising. The farmers are using and highly satisfied with the highly improved technologies and improved seeds to be more specific. Prior times, only one village in the woreda used Korra teff but it is widely used and applied now all over the villages’ particularly in the Dega weather areas in the woreda.

The farmers adapted the technologies and 50% if not fully 100% of them are using the technologies more than the other times. They are also growing beans to enhance the soil productivity for the nitrogen fixation. To reduce the soil acidity in desert areas, the respondent said, they gave training for 62 farmers so that they can practice acid reduction method.

Row planting is practiced for the maize and wheat seeds. This seeds more of productive and the majority of farmers who produce them are food secure. Dendea and Korra types of improved wheat and Teff seeds are widely being used. Before AGP, these improved crops did not exist in the villages of the woreda. Particularly for the Dendea, the productivity is increased with the AGP and it can resist and endure different weather types. It bears products, and the need to have stable products throughout the year regardless of the rain amount. Although the productivity may get reduced, the seeds won’t end up out of use in comparison to other seed types.

**Row planting:**

The farmers’ attitude about row planting is changing through and it is of radical one. The DAs and experts show the practice and the farmers followed and applied those practices. It reduced the labor need on the agricultural farm. The expert shows the farmers on how to apply and the farmers followed them accordingly. The farmers used to disfavor row teff planting, but now, it’s been widely practiced and even without the support of the experts. The row teff planting used to be strange, but it is now found that, row planting enhanced the productivity, and the seed consumption increases.

There are also benefits related to labor cost when it comes to row planting. There is only two people need to work on when it comes to row planting. However, the traditional way of planting requires much more people than that. Everyone can practice the farming, girs, women, boy and elder and all community members.

The productivity is high when it comes to the row planting. Seed consumption on one hectare can be as small as 15 kg, but the other one requires 25-30kg for the teff seeds. The wheat seed does not have weight, and farmers can weed out unnecessary one. Row planting is also practiced on using the maize see of BH-540 in low land areas in recent years. Once they started row planting, weeding is simple, and inter-cropping, is also feasible.

**Clustering:**

The respondent has said that maize and teff is practiced using the clustering mechanism. It however depends on the agro-ecology and the farm land size. It is not practiced in lowland areas due to the weather condition and small farm land size. But in highland areas wheat clustering are practiced.

Clustering helps to work at the same time and they can halt problems that might happen due to ploughing at different times and disruptions by human and animals during the first sowing and product collection phases. However, the respondent has said, clustering may not have that effective impact on the commercialization process. As the productivity increases, there may be individual based commercialization, but AGP has rarely accomplished its plans in this regard. The respondent has reported that the market linkage enhancing meetings and programs are usually held and there is a believe that clustering can build the fertile ground for the commercialization. However, this development still at the idea phase and it did not get into practice yet.

**Improved technology and its effect on farmers:**

There is a wide belief that there is increasing productivity in the areas. For instance, the maize was not considered as one of the main crops in the area, but after the AGP it becomes mainstreamed and becomes the usual and people are traditionally practicing it on the regular basis and with that their income and food Security also increases.

These crops area also not susceptible to the different plant infections, and when there is a sort of problem, the AGP also work on the chemical support among the farmers and they also produce awareness raising activities.

**Strength of AGP II:**

1. Encouraging farmers, their family members and the society in general to be adopting the different technologies and form CIGs.
2. Increasing the productivity is also strength of the program.
3. Getting to be aware of the environment and increasing the soil productivity is also the other strength of the program through the technological adoption.

**Weakness:**

1. Inability to create a market linkage despite the effort of fostering the commercialization.
2. Inability of farmers to practice the farming when they want to. The failure of farmers to fit in the farming calendar.

**Opportunities:**

1. The existence of the AGP itself is an opportunity.
2. Researchers participating in the analysis of the problems and ways to enhance their productivity. And giving trainings for the local farmers.

**Threat:**

1. Lack of inter-cropping different seeds if the clustering teff and wheats fails to produce a good outcomes.

2. Lack of adequate market and market accessibility.

**The Applicability (what should be done to attain the goals of AGP):**

1. Increase the stakeholders’ awareness. These stakeholders including farmers, DAs, and etc should be aware of the technology and its application.
2. Commercialization should be focused more, particularly he said, they need to work with the factors and different institutions.

**The perceptions of farmers:**

Farmers assume the AGP increase their productivity and production.

**Commercialization:**

Teff was sold at one point, and they sold to institution and individuals who need it for its multiplication. .However, there should be access to market linkages in all its forms.

**The area:**

The place is feasible and easy for the commercialization of wheat as it is produced less but the demand for the seed is high. Teff is less produced but demand is high, they want to sell the teff at the local level. Wheat is surplus and demanded less which increases the opportunity to engage in commercialization and sell the produce by transporting it to other areas.

**The commercialization center:**

There is no commercialization center to facilitate the commercialization endeavor. AGP focuses on the dairy processing. The area is prioritized for the dairy production. And hence, the area does not have the commercialization for seeds but for the milk.

**Consumption level:**

As the productivity rises, the consumption level increases. The farmers can consume three meals per day. They can also enroll their children to schools.

**FHH vs MHH**

They work on both on the households without discriminating against the type of households. They encourage youths to engage in CIG, and they encourage women and men also. They do not exclusively support either of male headed households nor female headed households. They do not have a gender disaggregated also.

**CIG and its applicability:**

The 24 men youth CIG and 19 women total 43 CIG were supported at the woreda level. They are engaged in various agricultural related activities and they focus more on animal fattening. Dairy Farming, Oxen fattening, Sheep production, Goat Production, sheep and goat fattening, and poultry production are the main types of CIG working areas. However,

1. Some groups dissolved their associations.
2. Few of them are still unified and getting more benefits- dairy production is exemplary in this regard.

The dissolution of groups is resulted from loss of interest in working together. In principle, they need to have similar interests and the criterion is that a member of a family can join a CIG and should be credit-free individuals. The age also matters for forming the CIGs. The working age (18-55) are preferred to others. The AGP helps 75% of the initial capital while the members contribute the rest. The money given is‘seal’ money (Seal-may not be the appropriate spelling). If a member leaves a group, its only profits that he/she takes out while leaving.

**The problems in CIG:**

There is lack of regulation and strict laws to abide the members and it is only the ‘seal’ money that is used to control members. The other problem is that when the farmers and graduate class is assigned to the same group, graduated students can leave in cases when they secure job in another places, hence this create a sort of conflict. The capital is also a problem. For instance by about 20 individuals, if the AGP gives them 100k birr, they can only be able to buy 3 oxen and sharing the profit by about 20 is imminent and can also be a reason for the dissolution of the groups. The budget allotted for groups is low and cannot be satisfactory among all. The buying capacity of money is also changing through time and it cannot fit the needs of the members now in comparison to the earlier times.

**Stakeholders:**

The support comes from all stakeholders like the livestock office, youth and women affairs, and cooperatives agencies. All have stake in the CIG groups. DA also working in cooperatives with the members and they give routine assistance and supports. However, the support rendered is not enough and there are yet to be done in satisfying needs. DAs also support but the main activities of buying the cattle’s and oxen excludes them and accomplished by the AGP, Cooperatives organizations, and the financial agencies of the woreda. The DAs may also consider the AGP activities as extra-work and they may not give as much attention as the activities need.

The other problem is lack of evaluation. The activities the CIG undergo do not accompany with the evaluation and their progress is not clear.

**New Development:**

1. Farmers who multiply the improved seeds, they can share them among themselves.
2. Lime to enhance the soil productivity was enhanced by the AGP
3. Bio-fertilizer is also introduced and considered as a new development in the area.

**What needs to be done to enhance the CIG?**

1. Guideline should be owned and the members should feel they actually own the group.
2. The guide lines should be in line with time and needs to be updated, and strictly followed.
3. The guidelines should also be developed by the team members themselves. When, why, and on what precondition should the CIG group members’ leave should be clearly stated. Whether and if the profit should be shared or not also needs to considered.
4. Livestock agency, cooperatives organization, youth, women and children and agricultural office all are stakeholders that need to be taken in to account, but there is no accountability and evaluation of the progress of whatsoever. There is no budgetary limitation in this regard, if not the limitation in women, youth and children’s affairs as they depend on other organizations particularly the Cooperative agencies. The prime problem is lack of evaluation.
5. DAs are also the sixth stakeholders and they routinely engage with the local farmers.
6. The cooperatives, finance, and youth affairs buy the animals. The DA should be involved though since they follow up the activities and they know more about the needs of the local farmers.
7. The involvement of the local village level is not considered either ways.
