# Supplementary material for: Effects of common interest groups on rural women and youth livelihood: A qualitative study from Central Ethiopia
Source: PLoS One. 2023 Oct 20;18(10):e0283532. doi: 10.1371/journal.pone.0283532 (PMC10588890; doi:10.1371/journal.pone.0283532)
Supplement: S20 File — (DOC) [file pone.0283532.s030.doc]

**Interview with the Lencho-Borsu-kebele’s DA_(Admassu Kebede)**

**The eligibility criteria for AGP II:**

The respondent has said the interest of the farmers is the basic requirement for starting a common interest group as its name imply. Other than this the establishment of the interest group also depends on the existing potentials as in such cases of irrigation programs. The respondents has said the development agents provide trainings for the farmers about the irrigation, seed planting, and then, the interest and farmers’ potentials are selected accordingly.

**The problems encountered while selecting the farmers:**

The respondent has said the farmers most often object to be enrolled in AGPs and its service particularly in areas where the irrigation is needed to take place and the farmers do not allow for the canal to pass through their farm lands.

**Practicability of agricultural technology demonstration:**

The respondent has said the demonstration of newly developed technologies are taking place in agricultural land among farmers particularly on farmlands of those farmers who are considered to be productive and exemplary for others. By demonstrating, the DAs show the effectiveness of improved programs and their elements. Clustering of the agricultural products also take place after the improved seeds area believed to increase the agricultural production and productivity. Demonstrating the technology helps farmers to get well informed, and adopt them well to their context, he said.

**The perception of farmers towards the AGP II:**

The respondent said that the farmers of the area assume the seeds will be given to them annually and they want the AGP addresses their needs in this regard. He said, they assume the AGP should provide them [the AGP beneficiaries] inputs and mostly they fail to produce improved seeds on themselves and they do not practice the training they acquire at the grass root level. The respondent has also said the farmers wrongly believe that the improved seed named Korra Teff is not productive around the wet and cold areas which mostly wrong as it can also be produced in wet areas despite the quantity difference.

**Strength of using Korra:**

The respondent has said the product does not fade away anytime, and it is helpful as it increases the productivity and amount of products. He also added since the price of Korra is expensive at it seed level, it helps the farmers to get more income by selling it.

**Weakness:**

The respondent has said the improved Korra cannot be used to feed the animals as it is hard for the food consumption.

**Future recommendations:**

HE said, the improved technologies such as improved seed types should be used widely among the farmers and they need to apply row farming while doing so. He also said seeds and fertilizer should be applied simultaneously and there should be improvement of technologies since the existing ones demand more labor and energy.

**Commercialization:**

The respondent has said the market linkage is not that active in the area and this obligated farmers to sell their products in their own local market and it is totally believed that the market itself is the one that decides the price of the product and the farmers do not have that great say on it. He said there is no market linkage in the area that have even created and facilitated by the AGP. He said had there been such opportunities, farmers could have garnered more benefits including more incomes.

**MHH vs FHH**

In the study area, more of male headed households participate in agriculture in comparison to the female headed households.

**Common Interest Groups:**

The respondent has said the CIGs are productive in the area and particularly the improving milk and milk productivity has been enhanced the income of farmers. In the area, the CIGs are notable in milk production and the seed multiplication; seed multiplication including Korra and Dagim.

**Productivity and improved seeds**

According to the respondent, Korra and Dagim are the two improved teff seed produced in the area. On a hectare, Korra can produce 28 quinatls of teff, but Dagim can be about 27 quintals while the traditional giving a product of 21. The improved technology needs more of fertilizers.

**Strength of the CIG:**

The respondent has said the strength is that grouping itself helps the farmers creating an opportunity of working together and accessing the market. It helps them easily and proactively access inputs, herbicides and pesticide as well. It also helps them to get more income which they cannot accomplish individually.

**Weakness:**

The main weakness of CIGs is that they are exposed to the conflicts and quarrels; there can be disagreements and conflicts among the members.

**New Opportunities:**

The respondent has said the CIGs improved the area and helped to build the already existing potentials among farmer but it is hard to count of any ground breaking new development that emerged due to the CIG or AGP.
