# Supplementary material for: Effects of common interest groups on rural women and youth livelihood: A qualitative study from Central Ethiopia
Source: PLoS One. 2023 Oct 20;18(10):e0283532. doi: 10.1371/journal.pone.0283532 (PMC10588890; doi:10.1371/journal.pone.0283532)
Supplement: S21 File — (DOC) [file pone.0283532.s031.doc]

**With the officer from the woreda’s Livestock and Fishery Development Office_ (Zeleke Hailu)**

**Introduction:**

The respondent has been working with both phases of AGP.

**How did you select members for CIG?**

The respondent listed the following criteria of selection

1. Interest
2. Resident of the village under consideration
3. Non-member another groups
4. Do not involve in any crime
5. Give prime focus for the graduate individuals.
6. One who can abide the rules of the CIG and cooperatives
7. Age is also another factor and they prefer people who are capable of working

The WALQO works on the business plan and the members start saving which is about 25% and the rest will be paid of AGP. In the sector, members mostly take part in animal fattening. The sector checks the animal about their health and the possibility of fattening. It is after that they buying the animal takes place and cattle bought by the CIGs are followed up for their best. They work on fattening the cattle to be able to get the product needed with three months and mostly with the support of technologies.

When buying these animals, neither the villagers nor the local DAs take part in the buying process.

CIGs exist in all villages all over the 25 villages. And the respondent said there is 2 CIGs in each kebele one of which is youths’ and the other belongs to women.

There is difference in their performances among the CIGS. Jemjem-Mela is exemplary in this regard, a Dhaye-Tuti and Lencho-Borso village is considered as better performing CIGs. Conversely, there are villages which are low performing due to the dearth of follow up. For instance, Shenkora-Shesheng which participated in goat fattening and production, and Aware-Golje is the other CIG that failed; and Wale-Chilalo is also another example. Medium performing are Jamo-Berdada, Olantu-Largi, Dambaza-Wole. In areas where stakeholder and members work well, the CIG categorized as better performing ones, but the other way around happen when the stakeholders and the member could not perform their respective responsibilities.

**Strength:**

1. It created job opportunities. In a given CIG, there are 20 members. So, it can create job opportunities for 40 individuals at the kebele level.
2. The money given by AGP, the money is a support to set an initial capital as of seal money. When they get more money, they pay the money back.

**Weakness:**

1. Lack of participation of local DA and the people in buying the animals.
2. Lack of follow up and monitoring. Because the sector has a lot of tasks to do and lack of budge also rationalize the failure in this regard. In a year, two or three times, CIGs can be met and follow up. Jemjem-Mela is the most visited area and followed up most often than others. And it is also used to share experience for other farmers.
3. Lack of ownership and coordination among the stakeholders.

**Implementation according the plan:**

The budget is limited but each sector has different plans. But there is lack of follow up and monitoring.

**Opportunities:**

1. The area is potential for cattle breeding and fattening. The AGP has proved that.
2. It enables the youth to build on their potentials and get employment opportunities. And the confirmation that anyone who works can get benefit and means of livelihoods.

The CIG has a great potential to create more opportunities if implemented well. And the respondent labels them as generally be a medium.

**What needs to be improved for better success?**

1. The guidelines related to CIG like how to buy the cattle is limited. Various stakeholder need to be involved. After the cattle is bought, the way it is followed up and the perception of member not to sell ahead of achieving the objectives is not targeted.
2. During buying the animals’ scientists assess the type of oxen, sheep, and goats and their probability of success. The problem mostly, however, lies related to fodder which most of the CIGs fails to gather. For that reason, the fattening period can be extended to four or more months. Similarly, the poultry needs much more care.
3. The local level DAs and village administrators monitoring and follow up is needed to achieve the better and reduce the problem by high as 50%.
4. There is benefit with reducing the members’ size for the management purpose. The productivity also rises. When the number increases, the productivity and the profit also decline. The basic reason to have20 individual is to pool the 25% money which mostly lacked in the village. There is no intention of creating employment by increasing the number of members since there are other projects working on creating opportunities.
5. There is a need to review the CIG practice starting from the initial stage of selecting the beneficiaries to the profitability. Every stakeholder and the members should be followed up and their level of implementing the objectives should be assessed.
6. CIG should improve a clear working pattern, about who should be included. The roles and responsibility should be clearly put and addressed in that way.
7. AGP helped the supply of improved seeds and increased the productivity in that regard.
