# Supplementary material for: Effects of common interest groups on rural women and youth livelihood: A qualitative study from Central Ethiopia
Source: PLoS One. 2023 Oct 20;18(10):e0283532. doi: 10.1371/journal.pone.0283532 (PMC10588890; doi:10.1371/journal.pone.0283532)
Supplement: S22 File — (DOC) [file pone.0283532.s032.doc]

**With the officer from the woreda’s Women and Youth Office_(Teshome Tolossa)**

**Introduction:**

The respondent has said the CIG was formed because of the awareness raising conducted in the villages found in the woreda mostly by the stakeholders. 50 CIG was planned to be established which is two of them in a village.CIG is established in collaboration with the cooperative offices. The main activity of the respondent is creating the awareness about the CIGs and its benefits after which the youths and women learn of joining. However, the respondent has said, there is no uniform knowledge among the stakeholders and the CIG members.

**Monitoring and controlling:**

The respondent explained that when there is a problem among CIG members, the stakeholders’ team reforms the CIGs. The respondent said they particularly focused on reforming of the CIGs where they are performing better. The CIGs do not have any controlling and punishment means that affected the effectiveness of the CIGs. The DAs are also reluctant to follow and control since they have not participated while buying the animals. The team is formed and they buy the animals for the CIGs. The local administrators do not participate in buying the animals also. They cannot participate because of the principles that only look for the engagement of animal workers and financial sector.

**Implementation:**

The respondent said they plan what they need to do but the effectiveness is not that much and it might fail. It takes much time to effectively reach the CIG teams and effectively inculcate them on board.

Internally, CIG members fail to provide saving. There is a problem while initiating the business and providing the contributions. Frequently, conflicts rise among themselves also due to absenteeism and neglect which ended most of groups in dissolution. The experts are not well behaving also. They are reluctant to reach the CIG members and may not appear in the village and not know the members and the members may not know him also.

The respondent said the applications and implementation of the CIG can be considered as a medium and despite the failures that occur most often, the members are benefiting on one way or another. Once the group members have joined the CIGs, they can engage in other livelihood activities based on the income they garner from the membership.

**Ways to improve:**

The respondent argues that there is a need for continues support from the experts, particularly the fodder types that can enhance the productivity of the oxen. There is need to formulate regulations as to what to happen to individuals who happen to break rule and regulations. The existing the stakeholders are enough, but particularly the local DA needs to involve better. The rural DAs should consider the AGP as their responsibility also. They lack a separate budgetary allocation for the DAs but for the woreda level stakeholders, they are getting benefits and salary. There is a need to market linkage.

**Opportunities of CIGs:**

- Employment
- Productivity

**Weakness:**

- Lack of market linkage
- Lack of proper application of plans at the local level
