# Supplementary material for: Effects of common interest groups on rural women and youth livelihood: A qualitative study from Central Ethiopia
Source: PLoS One. 2023 Oct 20;18(10):e0283532. doi: 10.1371/journal.pone.0283532 (PMC10588890; doi:10.1371/journal.pone.0283532)
Supplement: S23 File — (DOC) [file pone.0283532.s033.doc]

**Interview with the Abo-Yayambana kebele’s DA (Tesfaye Tewabe)**

**Beneficiary groups selection:**

The respondent has said that they grouped farming households in to high income households, medium and low income groups. And the wheat seed multiplication program, which was aligned with training provision, cluster farming, was directed for the high income households. In addition, they focused more on the farmers who are well informed about the production and productivity, and those who used the CIG and member of any program that works on increasing the production and productivity is considered as well informed of the technology adoption and subjected for the benefit of AGP. Furthermore, he said, the farmers also need to have a minimum of half of hectare to participate in seed multiplication project.

The respondent also added that clustering often practiced during such seed multiplication activities and they also scale up the farming areas of the households who possess more than 2.5 hectares. He, furthermore, highlighted that although the clustering of the farming takes place among many farmers, however, scaling up is restricted to be used among farmers who are capable of sharing information and frequently happened in areas where the FTC is not available so as it can be used as alternative experience sharing platform.

The respondent generalized that they work with the kebele level stakeholders to select the direct beneficiary based on the farmers’ prior knowledge and experiences so that he/she can readily share them with others. While trying to form an ICG, they need to have similar interest with to-be-members and should convey high level of commitment and motivation.

**Problems encountered on the farmer selection:**

He said there are moment when the farmers quarrel with the stakeholders since most of the farmers wants to involve and participate in ICG and AGPP Program. Since the program also focused on on those who had information about the program and related know-how, most farmers may fail to be eligible to participate and they express their frustration with these requirements. The inputs are not arriving on time and their quantity has also been questioned by the farmers.

**Did the demonstration of agricultural technologies take place?**

The respondent said whenever new technologies is introduced in the area, demonstration of how to use and adapt it take place through FTC. The demonstration highlights how the improved teff types supposed to be applied in a way that gives maximum outputs and enhance the productivity. In those cases, the respondent, new findings can also came out inadvertently. For instance, while demonstrating the Korra teff, they came to know that soil soil type and its acidity values matters in the process. Nevertheless, they confirmed that the improved seed of teff produce maximum production in comparison to the traditional seeds,

In addition, he said they showed the way improved wheat named Dendea, Boset, and Digalu which took place on the farmer’s lands. In the previous times, the farmers used Digalu but because of the good result achieved through demonstrating the Dendea seed type, now days they inclined to use it instead of Digalu or Boset, he said. Moreover, the productivity of the former seed is also high in comparison to the later which add the rationale of using it.

In general, the respondent has said that they consider demonstrating new seed types as effective since it helps farmers to raise their productivity. This is exemplified as the respondent claimed that on a hectare, they farmers could mage to produce about 25-30 quintals of wheat, and they sell the product for 26-30 per a kilo. He also said the bi-products can also serve as a fodder for the cattle population. But the traditional seed of wheat used to give up to 15 quintals per a hectare and the farmers did not use technology, fertilizer, and they do=id not practice row cropping formerly since thse products are not adequate in the area, and those who used it depend and borrow these products from their family and friends. The other important side of the Dendea as its name is that it is not affected by the rust and can resist the infection emanated from the insects and plant worms.

**Perception of farmers towards the technology:**

He said the local farmers are keen to use the improved seeds to enhance their productivity. However, most of the inputs and technology used do not commensurate their interest of using them.

**Strength of Dendea improved seed:**

The respondent has said that the seeds improved the productivity among the farmers and it made the produces available incessantly. It can also be readily adopted by the farmers, and they also practice the row planting.

**Weakness:**

The respondent has said they cannot continuously use the improved seed types since it is limited in supply. Particularly, they often times problem when they need the input the most during the production season. There is also a sign that the productivity of the improved types depend on the agro ecology and it needs the farmers to keep the seeds produced in previous year since they are assumed that they already adapted to the ecology of the area.

**Commercialization opportunities:**

The respondent has said AGP planned to create market linkage with the potential consumers of the wheat, however, the there was no that effective commercialization endeavor to take place in the area. The AGP formed in for the improved seeds with the Biftu Selale Union, but that linkage was not as great as it supposed to be.

**Problems:**

He said they face problems in the production of the improved seeds and the utilization and production amount is not clear if they are mostly used for household consumption or available for the sell. The Wheat they sell at the local market is not paying them well and it all depend on the middle men capacity to negotiate the price and the farmers rarely have any says in that regard.

**Technology usage and Consumption:**

The respondent highlighted that:

1. As the productivity increases, the income increases and the household level expenditure also increase.
2. The more the productivity increases, the more they consume good and services for their households.

**FHH/MHHs**

The respondent has said the FHH exist in the village. And there are cases when they are effective in the business and can also be exemplary for many. The AGP however, serves both the FHH and MHH equally.

**CIG group:**

The respondent has said that one of the criteria for selecting the farmers for the CIG are that they had to have common interest and initiation to work together for change. In the village, two CIGs availed and one consisted of 12 men who fatten oxen and the other group which consisted both men and women produce sheep. However, as the time goes, their productivity declined and later the groups were dissolved. Nevertheless, these groups benefited from the CIGs as their income and livelihood increase even of the group was dissolved because they shared the cattle population at their final time.

**CIG strength:**

The respondent has said that the money collected from the members and the money given by the AGP is feasible enough to let them engage in the activities. He said that helps the coordination among the farmers to start the business the sooner.

**Weakness:**

He said the continuity is always a problem despite the fact that the livelihoods of the members in one way or another are always improved.

**New development in the village:**

- The training and inputs were provided for different groups of the society, and that improved their productivity. The clustering system was informed and effectively carried out.
- Media coverage and experience sharing took place.
- It motivated many farmers to get new services.
- The DA also get got income in addition to the regular salary.

**Future recommendation**:

He recommended that the place where the animal are about to be kept, their feeding system and labor forces should be adequately prepared before forming the CIG group.
