# Supplementary material for: Effects of common interest groups on rural women and youth livelihood: A qualitative study from Central Ethiopia
Source: PLoS One. 2023 Oct 20;18(10):e0283532. doi: 10.1371/journal.pone.0283532 (PMC10588890; doi:10.1371/journal.pone.0283532)
Supplement: S24 File — (DOC) [file pone.0283532.s034.doc]

**With the officer from the woreda’s cooperative development office (Debebe Zeleke) _0946361376**

**Introduction:**

The cooperative office of the woreda takes parts as one of the stakeholders in AGP. The cooperatives particularly want to encourage the CIG members to enhance their livelihoods and join the cooperatives-the multi-purpose cooperatives.

**Problems:**

The problem is particularly how to handle the members without the dissolution. There is no guidelines that can hold the members together and how to guide if they actually dissolved. The CIG has criteria to select the members, but the one who dissolved the group is not taken in to account for their actions.

**AGP 2: How many groups and members exist?**

There are 44-46 CIGs established during this AGP 2 period.

**Program Implementation: how it is progressing?**

The cooperatives help saving, enhance their income level, and encourage them to join the local cooperatives including the saving and credit cooperatives and multi-purpose cooperatives. However, many problems exist for this implementation. This is because the members are mostly of poor. The CIG is based on the monthly payment which they cannot and the cooperatives also require them buying sharing during the time of joining one them. However, those better off individuals are joining the cooperatives. The poor does not fit these expectations. In addition, the conflicts most often rise among the members and the way they are organized was a problem for the later conflict among the members.

There is a coordination lack among the stakeholders to better off CIGs. The rationale behind this is that the villages are huge and bulky which restrain stakeholder to meet all of these places. The workers at the village level are not considering the village level as their main activities and there is no evaluation in that regard that much and even if some motives exist, it is not effective as the local DAs are not showing that much dedication-they do not meet the CIG groups on the daily basis but they only report the progress merely for on the day of annual reporting and evaluation. This emanates from the fact that the local DAs mostly lack of owning the CIGs and they consider them as outsider and of NGO ownership. They assume they are working but it’s not the ultimate responsibility required from them, but there directions to work on that regard.

**Strength of CIGs:**

- The way CIGs are organized, based on their interest, formulating proposal, and giving the inputs away is the strength.
- The existing stakeholders who are supposed to help a given activities is also considered as the strength.
- The initiation to develop CIGs and more or less supporting and monitoring is also the strength.

**Weakness:**

- CIGs are not equally beneficiaries and the members face drop outs. The failure to enhance the members to join cooperatives.
- Inability to solve conflicts and failure to sustain the CIGs is also another weakness.

**How to solve:**

It needs the stakeholders to work together and they need to sustainably work with the CIGs to enhance their status and group performance.

**Do we need more stakeholders to solve the CIG problems?**

There is a need to involve the administrators at the woreda level. The local village administrative bodies are already doing their job but to include the local village level needs the job to be done at the woreda level. So mainstreaming the issue at the woreda level helps achieving a further development at the village level and involving the woreda administrative organs help to push the local administrations and normalize the trend of working with the local CIGs.

**Do we need commercialization agents?**

The respondent has said, yes, it is needed but the CIG are not developed enough to that level yet. As CIGs develop, (The office of market development) market linkage is a way important and they need market linking agents in this regard. The respondent said the dairy cooperatives may help, but there is no that great numbers of CIG working in dairy farming and there is limited number of them and they supply low. SO, there is no motive to link them with the organizations found in other areas. But in the future, it is likely that they can arrange in this regard.

**Performance of CIGs:**

There a high development of CIGs that sold 4 or more times for instance the Jemjem CIGs. The other few also sold their products once or twice and benefited. Others CIGs can be considered as low performing CIGs and such CIGs dissolved.

In light with the plans, the respondent considered the CIG performance is mostly of medium. The plan is how to raise the benefits but some are dissolved but they also benefited. So they consider the performance of CIGGs is medium performing in general.
