# Supplementary material for: Effects of common interest groups on rural women and youth livelihood: A qualitative study from Central Ethiopia
Source: PLoS One. 2023 Oct 20;18(10):e0283532. doi: 10.1371/journal.pone.0283532 (PMC10588890; doi:10.1371/journal.pone.0283532)
Supplement: S25 File — (DOC) [file pone.0283532.s035.doc]

In order to address these gaps, but as far as data availability allows, the main emphasis in this paper is on the implication of Common Interest Group Approach for rural livelihood change [It’s economic and social impacts]. This study was, hence, conducted the objective of examining the effects of AGP II on the operation of the Common Interest Group arrangements. With this it aims to investigate the implication of Common Interest Group Approach for rural livelihood change [It’s economic and social impacts]. In order to achieve the mentioned objective, the following research questions were identified.

- What are the activities performed by Common Interest Groups at the study area?
- How effective is the implementation of activities of Common Interest Groups at the study area?
- What is the SWOT for the Common Interest Groups implementation at the study area?
- What intervention strategies could be designed to enhance the operation of Common Interest Group in the study area?

**FGD_1**

**Introducing the group**

**Kebele:** Lencho-Borsu

**Business type:** Diary farm

**The name of members is:**

1. Dejene Tsegaye-Group Leader
2. Ketema Tolassa- Secretary
3. Wesene Kefale-Controller
4. Sheleme Mitku-Member
5. Teshome Ayalew-Member
6. Kefale Beyene-Member
7. Ketemash Ayele-Member
8. Tesfaye Tsegaye-Member
9. Jissa Negesse-Member
10. Kuba Shiferaw-Member
11. Kidanu Ayalew-Member
12. Geremew Tolassa-Member

The group was formed from15 men and 4 women totaling 19 members. When they commenced the business, they were given 90,000 ETB by the government and its program of AGP, and on that each of the members saved 1250 birr which totals about 27,800 birr. The saving from the members was used mainly to construct an abode, a place to keep the cattle (cows and calves). With the financial support from AGP, they bought 8 cattle mostly of which are cows which were meant for the diary production. The group had a total of 17 cattle, and sold two of them in the last three years.

**The Nature of their participation in buying cattle**

The discussants said they have participated in the process of buying the cattle particularly after they consulted with the AGP experts that they need to buy the cattle that better adapt to the local weather condition. The members and the technicians from AGP together bought the conducive cow type that can give a better product and adopt better to the local weather condition. From their participation, the discussants said, they benefited as most of the cows bought are mostly of productive if not one of the cows that fail to give good milk but a calf which was sold for about 7000 birr.

**Problems**

The discussants illustrated a lot of problems that the encountered in their business:

1. There is no transportation service in their village to be able to transport the milk from their village to the nearby urban place. They said, since there is no transportation service in the village, they cannot transport the milk and this triggered them to depend on only on butter production and its sale as the major business activity.
2. The discussants have also said there is no electricity in the village to use refrigerator and keep the milk healthy for a long time until they sell it.
3. Moreover, the discussant said they have been facing problems with the inputs for the cattle (Cow and calves).They said the forage for the cattle are from the local grass and farm bi-products who costs is increasing through time. There is this type of grass called ‘*Shakke*’, but since it needs fertilizer for its production, it incurs them more expenses.
4. Finally, they said the money they received from AGP was quite small to unlock their potential and benefit from the milk production. Since the money is small, they had to buy less quality of the cows just to satisfy their need to start the business. It did not find them well their potential. They said had it not been the case, they said, they could have bought a more productive cows.

**Roles and responsibilities**

Each members of the group is responsible to watch after the cattle and feeding them. They said, they allocate days and time for the members who would take care for the cattle once in 19 days since they are totally 19 members. A member on his/her duty day would watch the cattle, clean their abode, and feed them.

In addition, the discussant said, the members have obligation to abide the rules and regulation of the group and if there is a failure in that regard, they would face a punishment of 30 birr in the first instance, 50 birr for the second time. But the members shown increasingly important and they genuinely support and abide the rules and regulations.

The accountant and monitory body of the group are also obligated to save their financial resource to the local bank on time and withdraw when the group interested to do so, and mostly on time. The monitoring body also follows the members and their activities, the cattle and the financial budget.

**Expenditure**

The discussant explained that the big expenditure of the group is to buy a fodder for the cattle. They say this expenditure is a way great and increasing through time as an example if the grass was 2000, birr it is 50000 birr now. The by-product of a teff which was 200 birr two years ago costs as high as 5000birr now.

These expenditure does not commensurate with the income they garner which is from butter sale only thus far because of the problems mentioned above, particularly transportation, electricity, and fodder related problems. They said the income for the butter can be of 500 birr per month on average, but the expenditure can be 4000 birr per month. However, they said they also benefited from the group in that they were able to buy 7 more cows in the last three years. Nevertheless, the income they garner is lesser of their expectation.

**Market Linkage**

The discussants said there is no market linkage and they sell their product-butter, on the own. They sell 5 kilo of butter one in a week at the local market place.
