# Supplementary material for: Effects of common interest groups on rural women and youth livelihood: A qualitative study from Central Ethiopia
Source: PLoS One. 2023 Oct 20;18(10):e0283532. doi: 10.1371/journal.pone.0283532 (PMC10588890; doi:10.1371/journal.pone.0283532)
Supplement: S26 File — (DOC) [file pone.0283532.s036.doc]

**FGD_2**

**Introducing the group**

**Kebele:** Dhaaye-Tuti

**Business type:** Sheep fattening

**The name of members is:**

1. Fayye Warku-Group Leader
2. Tolli Aseffa-Secretary
3. Dajane Lamessa-Accountant
4. Solomon yazo-Member
5. Tadese Fayyisa-,member
6. Tadese Debele-Member
7. Lami seyyoume-Member
8. Habatamu Haile-Member
9. Tolessa Tamiru-member

**Tolli Aseffa, secretary of the group has introduced the group in the subsequent way:**

The respondent said the group he belongs to which has 10 members started to operate shortly after their formation in 2010 E.C. Though the group whom all are men has no name yet, it is located in Dhaaye Tuti village/kebele

The respondent stated that they came together and form their group basically based on their self-reported interest which of course supported by the awareness raising campaign held in their village and at the woreda level as well. The group also opted to fattening and producing sheep and he reiterated that choosing the sheep related to their interest but did not highlight the specific rationale of choosing the sheep production. They rented a place where they can keep the sheep. Since the sheep are many, they shared them among 5 of them also.

**The group and its effectiveness**

The respondents have said that when they commence the business, they were able to raise 1660 birr from each of the members and supported with 50,000 birr from AGP II with which they bought 55 sheep. They explained that the group membership was enabled them to work collaboratively, and it was not merely for the purpose of revenue making but meant for sustaining livelihoods and generating incomes for their daily needs. They also vowed that they are not going to dissolve the group or there is no such ambition of quitting the membership when and if people leave which did not happen yet. They also said the group will exist regardless of the capital or the risk they might face.

The business helped the members to get employed which most of them used to dearth and their productivity is increasing through time. They mentioned that when they sold the sheep for 62,000 birr in the first year of their formation and they shared the revenues among themselves and each got 6000 birr. They had to share the revenues at the time basically because, they said, they need money to buy agricultural inputs such as fertilizer. They also said, of course they are selling sheep then on but the 62,000 birr they once got is the highest income recorded. Moreover, although initially they had 55 sheep, at the time of this interview, they have about 80 and more sheep now. Emphasizing their effectiveness, they also added that despite the income sharing they have to conduct when they want, they said they also have unofficial amount of money with the group accountant. They also recall that during the beginning of the business they had lost numerous sheep for the dead.

**Perception of working in groups and relation with others**

Being in group entity itself has its own positive sides, say the respondents. Working in group gave them numerous benefits which each of them could not accomplish on their own and at ease. More importantly, it is providing a sustainable means of livelihoods and employment for the members.

Besides the benefits side, however, the respondents illustrate that it was difficult to get the enough farming place for their sheep. They stated although the government promised to provide such area, it did not live up to their promise neither did materials used for construction is provided by the governmental entities.

They also said although another group consisting of women exists in the village, but they were not that close to them despite rare information they had to share among themselves.

The other perception area examined by the interviewer is whether the members assume the initial money they had to contribute is expensive. They responded that the contribution was not that demanding for them but of course some members felt a bit of pressure to fulfill the expectations easily. They also said the group members knew that the contribution was meant to initiate the business and it will be saved for the future risk aversion.

**Sheep production and market linkage**

The respondents have said that they were told by the local government that some kind of market linkage would be created for them during their initial meeting. However, they criticized that there was no such kind that has been facilitated thus far and they depend on the local market for the marketing purpose. The market linkage facilitation didn’t happen yet. This happened, according to the respondent, that because the government entities, AGP II coordination office, and CIG groups by itself are all reluctant to do so regardless of the group’s attempts of producing more sheep as the years pass.

**Strengths of the group**

The respondents have said that the group membership is beneficial as it enabled them by creating job opportunities as stated above. They added, it created a sort of social networking among the youths which help them build social capital on which they can depend during the hardships. They also mentioned that membership in the group do not consume all of their time and rather it provides opportunities to generate income by participating in other income generating activities.

**Weakness of the group**

It was reported that the sheep did not get that much better satisfied and well arranged protection. They said they cannot endure protecting the health of the sheep due to the conventional feeding practice they have been at it, and they added that failure hurts their effectiveness in terms of income and related benefits. As to them the treatment of the sheep is not scientific yet, and they did not monitor them carefully which is because of the huge number of sheep.

**Opportunities for the local people**

The discussants said they increased the input for the local market so that the local people can satisfy their demand for sheep for various purposes. They also said the local people also learned that the fattening and producing the sheep is a relevant and feasible business in the area. Furthermore, the local people now a day are forming groups and doing the same after the group owned by the respondent and his team.

**Threats**

They said that during the first few months of the business commencement, they had to face problems like losing the sheep due to illness and related factor. And they stated that the disease is likely to even occur in the future unless some scientific interventions take place in the near future. This made them worried about their future and their business in the future.

**Strategies to benefit from the CIG groups (Recommendation)**

The respondents have said that in order to benefit more from such groups as CIG, they mentioned two strategies:

1. The CIG working areas and its components should be broadening.
2. Union should be formed from the groups and facilitate the marketing activities.
