# Supplementary material for: Effects of common interest groups on rural women and youth livelihood: A qualitative study from Central Ethiopia
Source: PLoS One. 2023 Oct 20;18(10):e0283532. doi: 10.1371/journal.pone.0283532 (PMC10588890; doi:10.1371/journal.pone.0283532)
Supplement: S27 File — (DOC) [file pone.0283532.s037.doc]

**FGD_3**

**Introducing the group**

**Kebele:** Wale-Chilalo

**Business type:** Poultry production

**Name of the discussants:**

1. Destaa Taraffa- leader-0976068813
2. Chikabe Dame..Member
3. Yeshi Seyoum-Member
4. Fikru (represented his wife who is secratory-Ejige Neggasa)…0935537533

**Destaa Taraffa, chairwomen of the group has introduced the group in the subsequent way:**

The discussants said they were 20 women when they start the business in 2010, and each of them contribute 1200 birr which they had to pay as part of the initial capital which accounts for 25% while the rest is contributed by AGP. They said the group was able to collect about 22,000 birr and they got 66000 birr from AGP. With that money, they said they constructed abode for the poultry. They also said AGP has given them 1200 small poultry but many of them dead with few surviving.

**The objectives of forming a CIG**

The discussants have said that their main motive of forming a CIG was for the purpose of upbringing the poultry and selling their products.

**Effectiveness**

The CIG has sold about 622 hens during their functioning time and able to garner 39500 birr in general and they shared 1975 from this output. However, the income they got did not commensurate the efforts they paid and the requirement for upbringing hens also. It takes three months for small hens to be grown fully, but the group could not feed poultry through these times because the feeding is expensive and can incur more expense than the income they generate from the production. The group does not have enough materials to locate and transport the poultry and their products also. AGP has also given them the poultry during the rainy season but the small poultry product require warm places which the group lacks. The discussant also said they requested the AGP coordinators to provide them the material supports on time, but there was no such assistance back then. The aggregate result of these problems is the dissolution of their group and membership.

The members said that they actually befitted when the CIG is dissolved since they shared the hens and their products among themselves and owned them privately. But they do not have a positive attitude for the group membership as they face more of risks and debts than benefits. They also reiterate that there was no quarrel and disagreement among the members and the malfunctioning of the group emanated only from the risky nature of the business.

**Any benefit from membership though**

The discussant said regardless of the malfunctioning of the group as it supposed to operate; they garnered benefits in terms of motivation and experience. They also said they were able to create social capital and the group served as a source of information sharing.

**Strengths**

The discussants said working together and solidarity among the members is what they consider as the strength of the group. AGP’s initiation and the members’ willingness and ability to fill the requirements on times such as availing the saving was also considered as the other strength of the members.

**Weakness**

They said the quick nature of pessimism and inability to give a recovery time was considered as a problem. They assume they should have waited for the intervention form the stakeholder before stopping the group.

**Opportunities for the society**

They said the nearby communities have learned the benefit poultry production can have since they used to visit the group; and they claimed that most of these visitors also established their own comparable poultry production businesses.

**Recommendation for the future**

The discussants have said they wanted to commence the business even now but they need support from the concerned bodies. Hence, there is a need for the prompt intervention from the government side and they said there is a need for the continuous follow up. They think had there been market linkage, they would benefit from the service at least their transportation cost can be reduced. They also underlined that the service related to poultry should be available on time during the dry season.
