# Supplementary material for: Effects of common interest groups on rural women and youth livelihood: A qualitative study from Central Ethiopia
Source: PLoS One. 2023 Oct 20;18(10):e0283532. doi: 10.1371/journal.pone.0283532 (PMC10588890; doi:10.1371/journal.pone.0283532)
Supplement: S28 File — (DOC) [file pone.0283532.s038.doc]

**FGD_4**

**Introducing the group**

**Kebele:** Abo-Yayambana

**Business type:** Oxen fattening

**The name of members is:**

1. Dereje Kebede: Chairperson_0922596825

2. Mesay Ayele: Secretary_0912026813

3. Tadelu Keffalew: Accountant

4. Aster Ababu: Member

5. Kaba Werku : Member

6. Boki Kebede: Monitoring and controlling

7. Chala Kabe: Member

8. Jare Teferi: Member

9. Inie Zergu: Member

10. Dare Alemu: Member

11. Asrat Molla: Member

12. Aster Tola: Member

Previously the group consists of 20 members but through times they become 12. The group established in 2010.

**Evolution of the CIG:**

The group was established in 2010. First, the stakeholders from the woreda came with the village leader and talked to them. The AGP II organized individuals who happen to have similar interest and residing in the same area. The village leader gave them the list and all of the listed individuals attended the meetings, and 20 individuals came together and form the group. They also took part in the woreda level training. They were given 70,000 birr.

The task of oxen fattening was chosen by the interest of the group members and they took training two times with. The members also saved about 24% and 14k in total amount. The members care for the oxen on routine. Some group members are, however, reluctant. The quarrel happened and they informed the woreda level stakeholders and they gave 8 individuals their saving and the group members were minimized to 12.

The oxen were bought from the town of Goha-Tsion/Qarre-Goha. However, the group wanted to buy but they can’t, and the woreda level individuals did not allow them to do so. 84000 birr in total bought about 7 but 14000 birr was confiscated at the woreda level.

They were told they would be given the place where they can keep their animal. And also materials used for constructing but they failed to do that. Then, the members decide to rent a place recommended by the village leader, and could not build a house for the rest.

The 12 members divided in to two having 6 members each. And later they divided in to three individuals and they kept those cattle at the side. They sold three times to the market thus far. They bought 7 more oxen which were of the best quality type, and they sold them within 2 months. In each of them, they got 700-1400birr revenue. The members wanted to buy more of oxen but they faced obstacles from the woreda stakeholders. Nevertheless, they later on bought 9 oxen and each group has 4 and 5 oxen. Although some fatten, others did not. This also created a conflict within the members.

In general, they sold the oxen three times and in the first round, they got a profit of 700-1400 birr from each, and the second round they got 1200 birr of profit from each. Other time, they got 110-1600 profit. They divided the profit among themselves, and but the stakeholders did not recommend that.

Due to various problems they had to face including the lack of inputs for fattening purposes, they dissolved the group and share the oxen among themselves. Nevertheless, they were beneficiaries even if the group is dissolved. For instance, the leader bought an ox for 16000 birr and later on sold it for 22,000 within 6 months. Most of them, however, shifted the oxen fattening for another activities like shopping, buying inputs and ploughed lands and etc.

**Perception:**

Although the business is perfect, the support from the government side was discouraging. They could not get service from the stakeholders; they lacked inputs and reserves where they can keep the oxen. They did not get training on the routine basis also. They assume there would have been more benefits had they followed the training appropriately.

**Market linkage:**

Although during the training, they were told they would get more access to market, that promise never existed. On the second term, none of the members got service form any of the stakeholders and they were discouraged in their business, they say.

**Strength:**

The members did not have any source of living before joining the CIG. It helped them secure some livelihoods. It also enabled them diversify their means of living.

**Weakness:**

The team lacked all rounded knowledge and proactive means of securing their business. They agreed that they should have worked to build reserving place for the oxen rather than excusing it for their failure and dissolution. They also lacked that cooperation and team-orientation.

**Opportunities for the local people:**

Other members of the community learned a lot form the team. The villagers asked for the experience sharing platform as well.

**Social capital of the team:**

The group is supposed to have the same interest and resides in the same area. However, as the number of members is increased, the effectiveness reduced because there were many ideas. Had the group build from only 3-5, they could have the same idea and become more effective. But as the number increased, there would be an increasing interest among the members.

**Problems from the local people:**

They were supported by then local people, but they did not negatively affected. Some of the members even gave the group a grazing place for the member and encouragement.

**Recommendations (what should be done):**

The respondents said, the number of group should be reduced and each of the group has to get to know one another and work as such. The people who builds a group should be those who live in the same residence area, know one another and their effectiveness and all of that and they need to select who should join and not. So, he said, when they are formed they should be from the same village and each of the members’ effectiveness and background should be assessed.

The government should also monitor and follow the group per week or a month. As of the respondent, there was no such monitoring activity from the government side. The governmental stakeholder did not clearly and carefully control the group’s works. Follow up should be aligned with controlling behaviors and punishments as well. The government should not be reluctant in that regard.
