# Supplementary material for: Effects of common interest groups on rural women and youth livelihood: A qualitative study from Central Ethiopia
Source: PLoS One. 2023 Oct 20;18(10):e0283532. doi: 10.1371/journal.pone.0283532 (PMC10588890; doi:10.1371/journal.pone.0283532)
Supplement: S10 File — (DOCX) [file pone.0283532.s020.docx]

Date: March 02, 2023

Ref: PONE-D-22-20401

Common Interest Group and its effects on rural women and youth livelihood: A qualitative study from Northwestern Ethiopia

PLOS ONE

Dear Dr. Rana Muhammad Ammar Zahid,

Academic Editor
PLOS ONE

**Subject:** ***Regarding the review and approval of the study by IRB (Ethics committee)***

The study under consideration is one part of the PhD dissertation entitled ‘Attaining Secure Rural Livelihood: Productivity, Income, Commercialization, and Welfare Impacts of the Agricultural Growth Program II Interventions on Smallholder Farmers of Northwest Ethiopia’. The Dissertation encompasses five objectives, and this specific study under consideration is one of the objectives of the broader dissertation. The editors indicated that PLOS ONE requires us to confirm that this specific study was reviewed and approved by an institutional review board (ethics committee) before the study began. However, the concerned IRB (i.e. the Institutional Review Board (IRB) for the College of Development Studies, Addis Ababa University) has begun the revision and approval of research proposal after we successfully defended our dissertation proposal. It only started a retrospective revision and approval for such a case. Before the IRB came into force, the Collage followed the following steps to make sure that studies adhere to the University’s scientific research methods and ethical data collection procedures:

1) The Center for Rural Development Studies review authors suggested alternative titles and has selected and approved the title that has national and international relevance along with the assignment of academic advisors and supervisors..

2. The author then wrote a dissertation proposal with the sagacious guidance and strict supervision of the major and co-advisors. The proposal was then submitted to the College of Development Studies of the Addis Ababa University after signed by the major and co – advisors. The College has then assigned internal and external examiners that assess the relevance, originality, and impact of the suggested study. The proposal is valid only when it satisfies both the internal and external examiners and is endorsed by both of them.

2) The tools for data collection were commented by two supervisors as part of the proposal.

3) We presented the proposal in an open defense session where the assigned examiners, read the proposal, ask questions about it, and gave us several comments for improvement.

4) The examiners looked at the research proposal's ethical and scientific components (in this way they checked for its feasibility and completeness).

5) The center made sure that our plan complies with the requirements of the institution and the research ethics before the data was collected.

5) Finally, one of the supervisors closely collaborated with the principal investigator during the data collection process and physically checked on the field to oversee the procedures for gathering the data, address any technical or logistical concerns, and ensure that the activities outlined in the proposal were carried out as intended.

As per the IRB’s procedure of retrospective review and approval, we have received a retrospective ethical clearance. Accordingly, we have attached Certificate of Ethical Clearance from the IRB. We also attached our dissertation proposal as a confirmation that the current study is part of the dissertation for which we have given an ethical clearance.

The specific name of the ethics committee/IRB that approved our study is indicated on the attached Certificate of Ethical Clearance.

With kind regards,

Solomon Zewdu Leul

Corresponding author, Doctoral candidate in Development Studies at Addis Ababa University, Ethiopia

[slmnzwd@gmail.com](mailto:slmnzwd@gmail.com)
