# Supplementary material for: Effects of common interest groups on rural women and youth livelihood: A qualitative study from Central Ethiopia
Source: PLoS One. 2023 Oct 20;18(10):e0283532. doi: 10.1371/journal.pone.0283532 (PMC10588890; doi:10.1371/journal.pone.0283532)
Supplement: Attachment — Submitted filename: Reviewer overall Assessment.docx [file pone.0283532.s040.docx]

**Reviewer Assessment Against the Criteria Set by the Publisher**

- **Criteria 1: The Study presents the results of the original research**

The reviewer has checked if the work is duplicated or not by going deep into multiple search engines and confirmed that this is an original effort by the authors. It is confirmed that the issue of establishing formal CIG is a recent effort pursued by certain NGOs in the country (Ethiopia). This makes this research a novel effort

- **Criteria 2: Results reported have not been published elsewhere**

As mentioned above the reviewer has gone through multiple search engines and did not find any results on any other published articles/manuscripts anywhere.

- **Criteria 3: Experiments, statistics, and other analyses are performed to a high technical standard and are described in sufficient detail.**

This article is fully a qualitative study. The authors applied extensive desk review, case studies (Collective Case Study), FGDs, and KIIs. The choice of collective case study by the authors is commendable as CIGs involve collective engagement and decisions. They have also applied the best and recent qualitative data analysis software (MAXQDA 2020). I believe this has helped them to clearly capture important thematic areas pertinent to the issues raised. The authors choice of plural data analysis methods (including thematic analysis, relational analysis, and content analysis methods) are highly commendable. What is more, the authors are guided by multiple theories including ABCD model. The conceptual framework is self-constructed which is hoped to reflect the existing situations of the CIGs and their performance in the study locations.

- **Criteria 4: Conclusions are presented in an appropriate fashion and are supported by the data.**

The authors rightly draw conclusions based on findings of the study that the reviewer did not see the need for revision except minor editorial (grammar, punctuations, and spelling) issues as indicated in the document. The conclusions are supported by the evidences/data collected for the study.

- **Criteria 5: The article is presented in an intelligible fashion and is written in standard English.**

The contents and the flow of the manuscript is superb with sufficient details and meet all the requirements of the publisher. Yet, the authors are expected to make certain editorial works in terms of meeting the standard English language requirements. I encourage the authors to go for an automatic standard spelling and grammar checks available in the Microsoft word.

- **Criteria 6: The research meets all applicable standards for the ethics of experimentation and research integrity.**

This research is not experimental that it does not require high level ethical approval. Yet, the authors made clear that they have obtained a letter of ethical clearance from Addis Ababa University. The researchers did not generally use procedures that could harm the subjects of the study. The authors also indicated that they have received the consent of the study participants. Thus, the research meets all applicable ethical standards

**Criteria 7: The article adheres to appropriate reporting guidelines and community standards for data availability.**

The authors have used appropriate reporting guidelines and community standards for data availability. They have indicated that all relevant data and Supporting Information files are within the manuscript
