# Supplementary material for: Effects of common interest groups on rural women and youth livelihood: A qualitative study from Central Ethiopia
Source: PLoS One. 2023 Oct 20;18(10):e0283532. doi: 10.1371/journal.pone.0283532 (PMC10588890; doi:10.1371/journal.pone.0283532)
Supplement: Attachment — Submitted filename: Response to Reviewers.docx [file pone.0283532.s041.docx]

Date: March 02, 2023

Ref: PONE-D-22-20401

Common Interest Group and its effects on rural women and youth livelihood: A qualitative study from Northwestern Ethiopia

PLOS ONE

Dear Dr. Rana Muhammad Ammar Zahid,

Academic Editor
PLOS ONE

We are very grateful to you for allowing our submission to enter into review process of your esteemed Journal. We are also thankful to you and to the reviewers for the critical comments given. We found all the comments given worthwhile and feel that they helped clarify and improve our manuscript. On behalf of all my co-authors, I would like to put on record their appreciation and valuable suggestions. Thus, we have revised the entire manuscript accordingly, as specified in the attached revised version of the manuscript with the highlights (yellow color) of the changes made to it. Please find the below attached thorough responses to the comments and concerns raised by both the editors and reviewers. Please find the below indicated thorough responses to the comments given and concerns raised by the editors and reviewers. Table 1 shows the responses given for the comments directly sent via email, and Table 2 indicates a one by one response to each concern and/or comment raised by the academic editor alongside their location in the revised manuscript.

Table 1: The responses given for the comments directly sent via the email

| **Commented by** | **S.No** | **Areas/issues commented** | **The comments** | **Responses** |
| --- | --- | --- | --- | --- |
| Editors | 1 | Style requirement | Please ensure that your manuscript meets PLOS ONE's style requirements, including those for file naming. | 1. Thank you! 2. We have ensured that our manuscript meets PLOS ONE’s style requirement. 3. |
|  | 2 | Ethics statement | Thank you for including your ethics statement.  For studies reporting research involving human participants, PLOS ONE requires authors to confirm that this specific study was reviewed and approved by an institutional review board (ethics committee) before the study began. Please provide the specific name of the ethics committee/IRB that approved your study, or explain why you did not seek approval in this case.  Once you have amended this/these statement(s) in the Methods section of the manuscript, please add the same text to the “Ethics Statement” field of the submission form (via “Edit Submission”). | The authors are thankful to the editors for appreciating our inclusion of ethics statement.  As per the request made, we have attached Certificate of Ethical Clearance from the IRB, and the letter that indicates the review and approval processes we went through in securing the Certificate of Ethical Clearance from the concerned IRB. Name of the ethics committee/IRB that approved our study is also indicated in the mentioned Certificate.  Additionally, as per the comment given, we have amended the statements about the ethical issues and inserted as an independent paragraph in the Methods section of the revised manuscript for further clarification, first by modifying the previous title of ‘Research design’ in to the new one as ‘Research design and ethical procedures’. |
|  | 3 | Caption insertion | Please include a caption for figure 2 | Thank you!  Caption for Figure 2 is modified, and it read as:  S2Fig: Analytical framework for the study on the CIGs effects on rural women and youth livelihood in Northwestern Ethiopia.  Source: authors’ construction. |
|  | 4 | Figure 1 (Illustration of the study area) | Provided that Figure 1 in our submission contain [map/satellite] images which may be copyrighted, it was recommended to present written permission from the copyright holder to publish these figures specifically under the CC BY 4.0 license, or (2) remove the figures from your submission. | Thank you!  We agreed with the recommendation given. Figure 1 in our submission contains the squeezed map/satellite images which made it impossible to label and describe, in addition to a copy right issue as commented. Thus, we have decided to remove the previous map of the study area and replaced it with another self-explanatory which is developed for this particular study. In light of this, the new map doesn’t have a copy right issue and is not squished and stretched. We also indicated its sources, and labeled and described the contents and specific study kebeles. Encompassing these features, we believe that the new Figure is now flawless, and incorporated all the comments given in this regard. |
|  | 5 | Captions for your Supporting Information files | Please include captions for your Supporting Information files at the end of your manuscript, and update any in-text citations to match accordingly. Please see our Supporting Information guidelines for more information: http://journals.plos.org/plosone/s/supporting-information. | Thank you!  We have included captions for our Supporting Information files located at the end of our manuscript in accordance with PLOSONE Supporting Information guideline. |
|  | 6 | Reference list | Please review your reference list to ensure that it is complete and correct. If you have cited papers that have been retracted, please include the rationale for doing so in the manuscript text, or remove these references and replace them with relevant current references. Any changes to the reference list should be mentioned in the rebuttal letter that accompanies your revised manuscript. If you need to cite a retracted article, indicate the article’s retracted status in the References list and also include a citation and full reference for the retraction notice. | Our reference list has been revised, and we made sure it is complete and accurate. And no retracted reference or paper is found. |
| Reviewers' | 1 | Is the manuscript technically sound, and do the data support the conclusions? | Reviewer #1: Yes  Reviewer #2: Yes | We are grateful to the reviewers for appreciating our manuscript’s technical soundness, and for confirming that the data support the conclusions. |
|  | 2 | Has the statistical analysis been performed appropriately and rigorously? | Reviewer #1: Yes  Reviewer #2: N/A |  |
|  | 3 | Have the authors made all data underlying the findings in their manuscript fully available? | Reviewer #1: Yes  Reviewer #2: Yes | We thank the reviewers for noting that we made our qualitative data underlying the findings in our manuscript fully available. |
|  | 4 | Is the manuscript presented in an intelligible fashion and written in standard English? | Reviewer #1: Yes  Reviewer #2: Yes | We are very thankful to the reviewers for valuing our Writing that it is presented in an intelligible fashion and written in standard English. |
|  | 5 | Review Comments to the Author | Reviewer #1: The manuscript technically sound and statistical analysis been performed appropriately and rigorously. The authors have discussed the results efficiently. Moreover, the manuscript has been written in appropriate way.  Reviewer #2: The comments are made on the manuscript and can be communicated to the authors. The reviewer has checked if the work is duplicated or not by going deep into multiple search engines and confirmed that this is an original effort by the authors. It is confirmed that the issue of establishing formal CIG is a recent effort pursued by certain NGOs in the country (Ethiopia). This makes this research a novel effort. This article is fully a qualitative study. The authors applied extensive desk review, case studies (Collective Case Study), FGDs, and KIIs. The choice of collective case study by the authors is commendable as CIGs involve collective engagement and decisions. They have also applied the best and recent qualitative data analysis software (MAXQDA 2020). I believe this has helped them to clearly capture important thematic areas pertinent to the issues raised. The authors choice of plural data analysis methods (including thematic analysis, relational analysis, and content analysis methods) are highly commendable. What is more, the authors are guided by multiple theories including ABCD model. The conceptual framework is self-constructed which is hoped to reflect the existing situations of the CIGs and their performance in the study locations. | We are grateful to both reviewers for generally appreciating our research work, and for the comments given. |
|  | 6 | PLOS authors have the option to publish the peer review history of their article ([what does this mean?](https://journals.plos.org/plosone/s/editorial-and-peer-review-process#loc-peer-review-history)). If published, this will include your full peer review and any attached files. | Do you want your identity to be public for this peer review?  Reviewer #1: **Yes:** Dr. Muzammil Khurshid  Reviewer #2: **Yes:** Yirgalem Eshete Fithanegest | NA |

Table 2: A one by one response to each concern and/or comment by the academic editor via the revised manuscript.

| **S.N^o^** | **Comments**  **[NB:** ***The texts on which the comments are given and the comments themselves are sequentially stated in this section, but only the comments given are indicated where such placement is impossible*]** | **Responses** | **Location in the revised manuscript** |
| --- | --- | --- | --- |
| 1 | Title  Groups  The reviewer inserted ‘s’ on the previously stated word of ‘Group’ in the title | Thank you!  The word Group was changed into Groups based on reviewer’s comment. | At the very first page and on page 1 of the revised manuscript, both located within the title. |
| 2 | ‘The Abstract located under the section of Manuscript Draft’.  “Please delete texts restated in green by deleting those written in red.”  What alternative tef types were adopted? | Thank you!  The texts restated in green were accepted as the substitute for the texts written in red, and all other editorial suggestions forwarded by the reviewer in the Abstract section were accepted. | Abstract, very first page. |
| 3 | Title  The previously stated title of the manuscript (i.e. Common Interest Group and its effects on rural women and youth livelihood: A qualitative study from Northwestern Ethiopia) is suggested to be restated in the following way:  “Effects of Common Interest Groups on rural women and youth livelihood: A qualitative study from Northwestern Ethiopia” | Thank you!  The suggestion is accepted. | Title, Page 1. |
| 4 | ‘Abstract’  Would you please refer to the comments made on the abstract above? | Thank you!  All the comments given on ‘Abstract’ at the very first page of the revised manuscript is considered, and Abstract in this section is rewritten accordingly. | Abstract, Page 2. |
| 5 | ‘Introduction’ | Thank you!  All the editorial, indentation and line spacing concerns suggested by the reviewer both in this Introduction section and throughout the manuscript are happily accepted with a due gratitude. These all are indicated in the revised manuscript with track change. | Introduction, Pages 4-8; and others in their respective sections. |
| 6 | Introduction  “Some words are very frequently appeared in the manuscript – one of these words is “remain” – I count about 15 words. Yet, there is no any grammatical problem” | Thank you! We have substituted some of these words with other appropriate words. | For instance in:  Results and Discussion, Page 15, Paragraph 1, Line 4. |
| 7 | Introduction  ‘…Rim & Nsanganira, 2019 pp. 3…  “Other references are numbered. Please apply uniform citation and referencing techniques throughout the manuscript” | We are thankful for the reviewer’s concern, and have replaced the authors name by number ‘14’ referring to the place in the reference section where the authors name is located. | Introduction, Page 6, Paragraph 1, Lines 6-7. |
| 8 | Introduction  ‘…Eneyew & Bekele, 2012; Schmidt &Weldeyes, 2019; Tedla, 2019; Worku &Woldetsadik, 2013.’  “Ditto” | We are thankful for the reviewer’s concern, and again like above the wrongly stated names of the authors are replaced by numbers (i.e. 17, 16, 18 and 19) numbers referring to the place in the reference section where the authors name is located. | Introduction, Page 6, Paragraph 1, Line 12. |
| 9 | ‘Research methods: Physical description of the study area’  “Please define local languages as a foot note” | Thank you!  ‘Woreda’ and all other local terms/words used throughout the manuscript are defined at their respective foot notes and at their very first use. | Physical description of the study area, Page 9, Paragraph 1, Lines 1, 3 and 5. |
| 10 | Research design  “What is this number?” | Thank you!  We inserted number 23 in the main text where the comment is provided to indicate the reference for the types of case study. But after the comment given, we decided to omit the inserted number since the reference indicated in number 24 by itself suffice to explain our intention. | Research design, Page 10, Paragraph 1, Line 2. |
| 11 | ‘Research design’  “Please include page numbers in all references or avoid page numbers from all references. Be uniform in terms of your citation style.” | Thank you!  Page number is inserted for the fact that we have used the direct citation here. As per the citation style we used, mentioning the page number when we used the direct quotation is mandatory. | Research design, Page 10, Paragraph 1, Line 5. |
| 12 | ‘Research design’  …kebele…  “Define local/unknown words in the foot note” | Thank you!  Defined. | Research design, Page 11, Paragraph 2, Line 5. |
| 13 | Theoretical underpinnings  ‘… (Kretzmann & Green, 1998)…’  “Ditto” | Thank you!  The reference mentioned as Kretzmann & Green, 1998) is substituted by number ‘36’ to indicate its reference number. By this, we made the in text reference consistent throughout the manuscript by substituting them, by their respective numbers they labeled in the reference section. | Theoretical underpinnings, Page 14, Paragraph 2, Line 3. |
| 14 | Theoretical underpinnings  “These are important principles against which the CIGs you have studied should be characterized. Are the studied CIGs citizen – led? Relationship – oriented? Asset-based? Place based? And inclusion focused? Which principles are adhered? Which are not?  Based on the answer for these questions, you can craft recommendations” | We appreciate the reviewer’s comment. Based on the reviewer’s comment, we have rewritten the last two sentences where this comment is given to indicate the ACBD model’s core principle we largely adhered to for the analysis part. Besides, based on the suggestion given, the first two recommendations were paraphrased to explicitly show the contribution and relevance of model for the wayouts forwarded. | Theoretical underpinnings, Page 16, Paragraph 1, Lines 2-5. |
| 15 | An overview of the activities performed by the CIGs  ‘Other stakeholders (women and youth affairs and cooperative development offices) criticize the procurement procedures.’  “Here you can also include the issue of transparency and trust.” | We appreciate the reviewer’s comment.  Based on the reviewer’s comment, to include the issue of transparency and trust we have modified the sentence which states as: “Other stakeholders (women and youth affairs and cooperative development offices) criticize the procurement procedures.” in to  “On the other side, other stakeholders (such as women's, youth, and cooperative development offices) lament the lack of transparent procurement system. For them, the lack of such a system negatively affects their initiation and causes them to doubt their convictions in this regard.” | An overview of the activities performed by the CIGs, Page 18, Paragraph 3, Lines 8-11. |
| 16 | An overview of the activities performed by the CIGs  ‘Being together without role specification could also result in the possibility to see group mates from the negative side than enjoying their different skills and experiences.’  “This could be better described as follows:  Lack of role specification among members could result in role confusion/ambiguity. It also reduces the benefits that could be exploited through specialization and experience.” | We are thankful for the reviewer’s comment, and we accepted the stated suggestion by only omitting the word confusion from the recommended statement since the word ambiguity better describes the issue under consideration. | An overview of the activities performed by the CIGs, Page 21, Paragraph 8, Lines 3and 4. |
| 17 | SWOTs of the CIGs  ‘…The SWOT analysis…youth empowerment system…’  “All these are not findings. Just move them to the methodology part. Here you need to present the finding about the CIG’s SWOT.” | We appreciate the reviewer’s comment.  As per the comment given, we moved many of the texts from the area where this comment is given to the appropriate place in the methods part (i.e. to the last paragraph of the Theoretical underpinning section); and rewritten the left statements there for more clarification as an introduction to the findings and discussion for the SWOT analysis section. | FROM the section of SWOTs of the CIGs, Page X, paragraph 1, Lines X to X TO the Theoretical underpinning section, Page X, Paragraph X, Lines X to X.  The modified/rewritten statements in the SWOT analysis section, Page X, Paragraph X, Lines XX to XX. |
| 18 | SWOT Assessment Matrix  “Instead of just listing down the Strength, weakness, opportunity, and threats of the CIGs, it would be great to present them in terms of their importance. For instance, out of the listed 7 Weaknesses, which one is most important (first) in terms of affecting their performance (efficiency and effectiveness)? The same for others.” | We are grateful to the reviewer for reminding us this.  The findings in all aspects of the SWOT were listed based on their importance (from most important to least important in terms of affecting their performance indicated by efficiency and effectiveness, as suggested). However, we didn’t state that they were listed by considering their importance). Thus, basing the remark made here, we have inserted the following statement in the introduction part of the SWOT (immediately before Table 4) to make it clear.  ‘Accordingly, the lists of major findings generated from the SWOT analysis tool and indicated in Table 4 are presented in terms of their importance (from most important to least important in terms of affecting their performance).’ | SWOT Assessment Matrix, Page, 30, Paragraph 1, Lines 4-6. |
| 19 | SWOT Assessment Matrix  ‘…and livestock’s irregular treatment were also found…’  “Do you mean that veterinary services are not regular?” | Thank you!  ‘Irregular treatment’ in this case is meant to describe the lack of knowledge on modern management of livestock including the provision of protection and rearing as indicated in the SWOT Assessment Matrix of Table 4. Hence, to clarify this, we modified the statement as follows:  ‘The non-existence of normative standards and legislation, lack of access to modern fodder, conventional feeding practices, and deficiency in modern livestock management practices were also found as weaknesses contributing to the CIGs' malfunctioning and dissolution.’ | SWOT Assessment Matrix, Page, 34, Paragraph 4, Lines 1-3. |
| 20 | SWOT Assessment Matrix  ‘…Since the activities…to group ethics...’  “This shall be rephrased to give sense. I suggest if it is restated as follows:  “CIGs are expected to operate as formal business enterprises. Thus, it is quintessential for CIGs to have a binding standard and legislation that would enhance their performance by creating an incentive to abide for common and group ethics.”” | Thank you!  We agree with the reviewer and based on the suggestion provided we have substituted the sentence which states as: ‘Since the activities, they expected to undertake are comparable with business enterprises, having business alike normative standards and legislation would enhance members’ performance by engaging them and reinforcing their commitment to group ethics’ BY: ‘CIGs are expected to operate as formal business enterprises. Thus, it is quite essential for CIGs to have a binding standard and legislation that would enhance their performance by creating an incentive to abide for common and group ethics.’ | SWOT Assessment Matrix, Page, 34, Paragraph 4, Lines 7-10. |
| 21 | ‘…FGD 3, October 29, 2020)…’  “Ditto” | Thank you! We appreciate the reviewer’s comment. Yet, we have inserted in text citation for the secondary sources or the materials we reviewed (i.e. author/s name) as per the requirements of the PLOSONE. For primary qualitative data, putting the right sources in the text is highly recommended, and listing these sources in the reference section is not recommended. Hence, we preferred to leave this and other comparable citation from both the FGDs and KIIs as they were. | SWOT Assessment Matrix, Page, 34, Paragraph 4, Lines 7-10; and all other citations from the FGDs and KIIs throughout the manuscript. |
| 22 | Recommendations  ‘…Since Micro and Small…countries like Ethiopia (96)…’  “This looks a literature review. There is no need for citation in recommendation. Just make your recommendation based on the findings of the study.” | Thank you!  We agree with the comment and modified the paragraph. | Recommendations, Page 40, Paragraph 1, Lines 1-5. |
| 23 | Recommendations    ‘…Since Micro and Small…many group members…’  “This could be summarized in less than 5 lines.” | Thank you!  We agreed to summarize the long and expanded paragraph. Accordingly, the texts stated in Paragraph 1, Lines 1-24 are  entirely paraphrased/rewritten and condensed in the subsequent way:  ‘While designing the intervention methods for CIGs, it is important to consider the value of knowledge and skill-based training both upon their establishment and while they are in operation (on-the-job training). Members need to have a very common objective and interest which needs to be established through a thorough training process. The ABCD model suggests the need for assessments before any intervention and explicates procedures for the assessments to be done.’ | Recommendations, Pages 40-41, Lines 1-24. |
| 24 | Recommendations    ‘It would have been better if the preparation …’  “Recommendations shall be strong.” | Thank you!  We accept the comment and the recommendation is made strong. | Recommendations, Page 41, Paragraph 2, Lines 1-5. |
| 25 | Recommendations  ‘The AGP II Program Design Document…’  “Please include this document as a reference” | Thank you!  The document was already included in the reference section. | Reference number 101. |

We sincerely hope that the corrections will meet with approval. If you have any questions about this manuscript, please feel free to contact us. Once again, thank you very much for your comments and suggestions.

With kind regards,

Solomon Zewdu Leul

Corresponding author, Doctoral candidate in Development Studies at Addis Ababa University, Ethiopia

[slmnzwd@gmail.com](mailto:slmnzwd@gmail.com)

[NOTE: If reviewer comments were submitted as an attachment file, they will be attached to this email and accessible via the submission site. Please log into your account, locate the manuscript record, and check for the action link "View Attachments". If this link does not appear, there are no attachment files.]

While revising your submission, please upload your figure files to the Preflight Analysis and Conversion Engine (PACE) digital diagnostic tool, <https://pacev2.apexcovantage.com/>. PACE helps ensure that figures meet PLOS requirements. To use PACE, you must first register as a user. Registration is free. Then, login and navigate to the UPLOAD tab, where you will find detailed instructions on how to use the tool. If you encounter any issues or have any questions when using PACE, please email PLOS at [figures@plos.org](mailto:figures@plos.org). Please note that Supporting Information files do not need this step.
